# Deciphering the Evolution Pattern of Structural Variations Overlapped With Repetitive Sequence During Cattle Evolution

**DOI:** 10.1002/advs.202523333

**Published:** 2026-03-09

**Authors:** Zhifan Guo, Jinxiu Li, Adeniyi C. Adeola, Xueyan Jiang, Juntao Ma, Jian Xiao, Dexiang Hu, Kaixing Qu, Haihong Wu, Junren Chen, Zhanxing He, Tingting Yin, Ali Esmailizadeh, Jing Luo, Olivier Hanotte, Ya‐Ping Zhang, Yan Li

**Affiliations:** ^1^ Bio‐X Center for Interdisciplinary Innovation and School of Life Science & School of Ecology and Environmental Science Yunnan University Kunming China; ^2^ State Key Laboratory of Genetic Evolution & Animal Models Yunnan Laboratory of Molecular Biology of Domestic Animals Kunming Institute of Zoology Chinese Academy of Sciences Kunming Yunnan China; ^3^ Sino‐Africa Joint Research Center Chinese Academy of Sciences Kunming Yunnan China; ^4^ Chuxiong Normal University Chuxiong Yunnan China; ^5^ Yunnan Academy of Grassland and Animal Science Kunming Yunnan China; ^6^ Department of Animal Science, Faculty of Agriculture Shahid Bahonar University of Kerman Kerman Iran; ^7^ International Livestock Research Institute (ILRI) Addis Ababa Ethiopia; ^8^ School of Life Sciences University of Nottingham Nottingham UK

**Keywords:** community ecology, repetitive sequences, structural variations

## Abstract

Repetitive sequences (REPs) are crucial for understanding the evolutionary trajectory of eukaryotic genomes, yet their evolutionary dynamics in domesticated animals have not been as extensively studied as in plants. Here, we integrated eighty‐three long‐read individuals from globally distributed cattle to provide a detailed landscape of structural variations (SVs) overlapping with REPs (hereafter referred to as rep‐SVs) in ecological contexts. Overall, rep‐SVs are unevenly distributed between the X chromosome and autosomes. Large rep‐SVs accumulated on the X chromosome, whereas certain types of rep‐SVs were depleted therein. These distribution patterns coincided with the subspeciation process between taurine and indicine cattle. A specific expansion involving several types of rep‐SVs occurred in indicine during this subspeciation process. Notably, this expansion highlighted the role of *PDGFD* (mediated by bovine‐specific Bov‐A2 REP), a growth factor that exhibits higher expression in the larger hump, a typical trait distinguishing indicine from taurine. Guided by compositional analysis, a rep‐SV in the *ROR2* gene (mediated by a satellite copy loss) was found to influence zebrafish growth, thereby potentially contributing to the large body size of the beef cattle breeds. Collectively, these findings clarify the patterns shaping REP dynamics in cattle and provide candidate markers for molecular breeding and evolutionary genetics research.

## Graphical abstract

The authors complement bovine pan‐SV with massive novel structural variations (SVs) identified through long‐read sequencing of 83 globally distributed cattle breeds. Repetitive sequence‐mediated SVs (rep‐SV) exhibit distinct dynamic patterns throughout cattle sub‐speciation and/or domestication processes, including uneven distribution between chr‐X and autosomes, specific accumulation in indicine breeds, and profound changes in differentiation. A satellite‐mediated rep‐SV was finally evidenced to be associated with large body size.

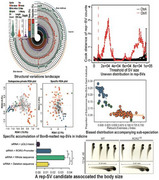

## 1 Introduction

Repetitive sequences (REPs) make up a substantial fraction of nearly all eukaryote nuclear genomes [1] and are pivotal drivers of genome evolution [2]. Homologous REPs give rise to the formation of structural variations (SVs) through mechanisms known as polymerase slippage, non‐allelic homologous recombination, non‐homologous end joining, microhomology‐mediated break‐induced replication, mobile element insertion, etc [3, 4, 5, 6]. Consistently, SVs are far more prevalent in repetitive regions than in non‐repetitive regions [7].

SVs exert larger biological impacts than previously characterized single‐nucleotide polymorphisms (SNPs), owing to their alteration of far more base pairs [8, 9, 10]. Mounting evidence underscores REPs’ multifaceted roles in cellular and organismal functions [11, 12, 13]. They are associated with 3D genome organization [2], chromatin modification [14], DNA methylation [15], and the modulation of regulatory gene expression networks [16]. Yet, despite this functional relevance, the genome‐wide evolutionary dynamics of rep‐SVs (SVs overlapping REPs) have remained largely unexplored in animals until recently [17].

From an evolutionary perspective, most rep‐SVs are likely “dead‐on‐arrival,” via pseudogenization or gene loss [18]. Yet, retrotransposons linked to genetic diseases often remain polymorphic in primate lineage or human populations [11, 19, 20, 21]. Domestication‐associated shifts such as relaxed natural selection on host fitness, strengthened positive selection toward specific phenotypes, and demographic bottleneck would also shape the fixation of newly emerged rep‐SVs. For example, transposons (TEs) have expanded and became fixed in the domesticated silkworms but not in wild silkworms, likely due to the genetic drift during domestication [22]. Similarly, a sudden and massive TE amplification was reported across different rice strains [23]. To date, rep‐SVs have been associated with economic traits and environmental adaptations in crops species, yet their roles in domesticated animals remain understudied [24]. For the latter, most genomic data stem from short‐read whole‐genome sequencing, which are unable to comprehensively identify rep‐SVs and resolve their genomic integration. Long‐read sequencing, however, offers a way to address these issues [25].

The domesticated cattle represent an ideal model for investigating rep‐SV dynamics across different evolutionary timescales, as they comprise two subspecies: humpless taurine (*Bos taurus taurus*) and humped indicine (*Bos taurus indicus*) [26]. They originated from two distinct ancestral aurochs: the Eurasian auroch (*Bos primigenius primigenius*) and the Indian auroch (*B. p. namadicus*), with possibly subsequent introgression from the North African auroch (*B. p. mauretanicus*) on the African continent [27]. Subsequent breeding process may have further shaped the rep‐SV distribution. Cattle also harbor bovine‐specific TEs, such as the long interspersed nuclear element (LINE) retrotranspon BovB and its derived short interspersed nuclear element (SINEs): Bov‐A2 and Bov‐tA [28]. Critically, the evolutionary history of the aurochs and the independent domestication trajectories of cattle collectively offer an advantage for dissecting the roles of rep‐SVs across diverse evolutionary timescales.

Therefore, we compiled a comprehensive SV landscape in cattle using long‐read sequencing data from 83 globally distributed breeds, with the aim of dissecting how rep‐SVs shape the bovine genome across different evolutionary stages and lineages. Notably, we identified a broad set of novel SVs that markedly expand bovine pangenome diversity. Furthermore, we characterized rep‐SVs’ dynamics over varied evolutionary timescales. Intriguingly, novel rep‐SVs were found to potentially influence phenotypic evolution during subspeciation and breeding processes. These findings not only advance our understanding of REP dynamics in cattle domestication but also offer valuable insights to improve cattle germplasm.

## 2 Results

### 2.1 Construction of a Comprehensive Cattle SV Landscape From Long‐Read Sequencing Data

We applied Oxford Nanopore long‐read sequencing technology to sequence the genomes of eight globally distributed breeds (Table S1): five Eurasian taurine breeds [Angus (AGS), a beef breed characterized by muscle traits; Simmental (SIM), a beef breed famous for its big frame; Yanbian cattle (YBC), a Chinese indigenous breed adapted to cold; Iran cattle (IRC), an indigenous breed living near the centre of domestication in the Fertile Crescent (a domestication center); and Muturu (MUT), an African indigenous breed with trypanosomosis resistance]; two indicine breeds [Brahman (BRA), a beef breed sampled from Australia; and Iran zebu (IRZ), an indigenous Fertile Crescent breed]; and Kuri (KUR), a taurine‐indicine hybrid from Africa. For each individual, over 120 gigabases (Gb) of ONT long‐reads were generated, yielding ∼45× coverage of the estimated 2.7 Gb taurine haploid genome, with an average read length N50 of 25 357 bp (Table S2).

**FIGURE 1:**
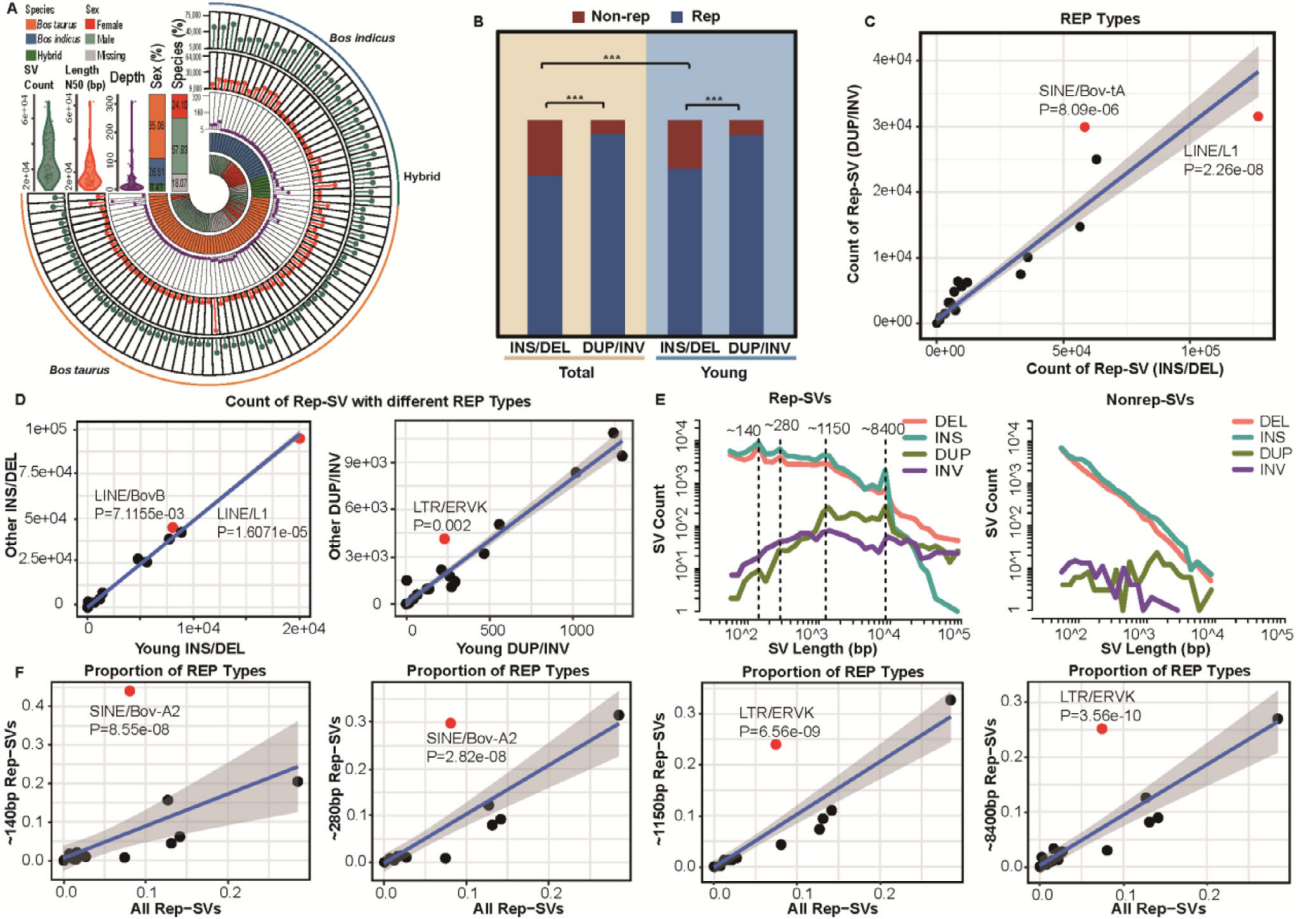
Summary description of panSV. (A), Demographics of the 83 individuals included in this study. (B), Proportion of rep‐SV and nonrep‐SV among total SVs and young SVs (including INS/DEL and DUP/INV). (C), Linear regression analysis of rep‐SV counts across different REP types, comparing INS/DEL and DUP/INV groups. Red dots indicated REP types that significantly deviated from the overall regression trend. (D), Linear regression analysis of young rep‐SV counts versus remaining rep‐SV counts for each REP type, stratified by INS/DEL (left) and for DUP/INV (right). Red dots indicated REP types that significantly deviated from the overall regression trend. (E), Size distribution of rep‐SVs (left) and nonrep‐SVs (right), stratified by INS, DEL, DUP, and INV. Vertical lines marked peak lengths in the four size ranges. (F), Linear regression analysis of the proportion of each REP type between overall rep‐SVs and rep‐SVs of specific sizes (from left to right: ∼140, ∼280, ∼1150, and ∼8400 bp). Red dots indicated REP types that significantly deviated from the overall regression trend.

To comprehensively characterize SV in domestic cattle, we further integrated long‐read sequencing data of 75 individuals retrieved from the NCBI Sequence Read Archive, resulting in a total of 6.50 Tb of long‐reads data with an average read length N50 of 19 958 bp (Table S3). This sequencing quality ensured robust SV detection via generally aligning these long‐reads from all 83 individuals to the bovine reference genome (ARS‐UCD1.2), with minimal influence of coverage variance indicated by SV detection rates on chr‐X between sexes (Figure S1). We then filtered and merged SV calls across all 83 samples, yielding 209 032 non‐redundant SVs (>50 bp in length) — one of the most comprehensive sequence‐resolved panSV genomes for domestic cattle to date (Figure 1A) [29]. In silico simulations suggested that the pangenomic SV distribution approached saturation (Figure S2). By comparing our dataset to previously published SV datasets (Zhou et al. [30], Gao et al. [31], Talenti et al. (a) [32], Xia et al. [33], Crysnanto et al. [34], Talenti et al. (b) [35], Dai et al. (a) [36], and Dai et al. (b) [37], with an almost exhaustive sample size), we identified 69 295 novel SVs (33.15% of total; Figure S3). These novel SVs markedly enriched the diversity of the bovine panSV genome, underscoring the unique utility of long‐read sequencing for capturing previously uncharacterized SVs.

Of all SVs, 51.25% were small (50–200 bp), 18.70% medium (200–500 bp), and 30.05% large (>500 bp). Most identified SVs (over 200 000) were insertions (INSs) and deletions (DELs); each individual also harbored dozens to hundreds of inversions (INVs) and duplications (DUPs) (Table S4). For genomic localization, approximately 67.58% of the ∼147k SVs occurred in intergenic regions, 28.44% in introns, and 0.85% in exons. This distribution aligns with prior bovine studies, which reported over two‐thirds of SVs localizing to intergenic regions [38].

Phylogenetic analyses based on either SVs or SNPs consistently resolved two distinct clusters: humped indicine and humpless taurine (Figure S4). African cattle KUR clustered with indicine individuals, likely reflecting indicine introgression into African longhorn taurine [27]. SV detection rates varied across cattle breeds, with generally higher rates in indicine breeds—likely due to the taurine origin of the reference genome (Figure S5).

### 2.2 Overall Characteristic Profile of rep‐SVs

SVs were classified as repetitive sequence‐mediated (rep‐SVs) if they (1) overlapped with at least one annotated repetitive element by ≥1 bp [39] and (2) had a maximal size of <100 kbp [40]. Given that repetitive fragments compose nearly half the bovine genome and repeat‐induced mutagenesis frequently causes SV, it was unsurprising that nearly three‐quarters of DELs/INSs (∼152k) were rep‐SVs. Notably, rep‐SV proportion was even higher in DUPs/INVs (Figure 1B; chi‐square test, *p* < 2.2e‐16). Bov‐tA occurred more frequently in DUPs/INVs, whereas LINE/L1 occurred more likely in DELs/INSs (Figure 1C; linear‐regression model, Bonferroni *p* = 8.09e–06 and 2.26e–08, respectively).

Examination of young SVs — those appearing as individual‐specific singleton SVs (Table S5) — provided an opposite pattern between INSs/DELs and DUPs/INVs. Young INSs/DELs had significantly higher rep‐SV proportions than total INSs/DELs (chi‐squre test, *p* < 2.22e‐16), whereas the ratio of rep‐SV to nonrep‐SV remained identical between young DUPs/INVs and total DUPs/INVs (chi‐squre test, *p* = 0.872; Figure 1B). For REP type associations, LINE/L1 occurred more frequently in young INSs/DELs (linear regression, *p* = 1.61e–05), while LINE/BovB dominated other (non‐young) INSs/DELs (*p* = 7.12e–03; Figure 1D). In contrast, no REP type preferentially occurred in young DUPs/INVs, whereas LTR/ERVK tended to mediate DUPs/INVs in non‐young rep‐SVs (linear regression, *p* = 0.002).

The frequency of INSs/DELs decreased sharply with increasing length, whereas DUPs/INVs were less sensitive to length changes (Figure 1E). Compared to INSs/DELs without REP, those with REP exhibited distinct size peaks (approximately 140, 280, 1150, and 8400 bp), which were highly overrepresented by certain REP types. Specifically, the former two size peaks (corresponding to length ranges of 125–158 and 252–316 bp) were dominated by Bov‐A2 (linear regression, Bonferroni‐corrected *p* = 8.55e–08 and 2.82e–08, respectively). The ∼1150 bp peak (1018–1263 bp) was enriched for LTR/ERVK (Bonferroni‐corrected *p* = 6.56e–09), while the ∼8400 bp peak (7686–9689 bp) was also LTR/ERVK‐dominated (Bonferroni‐corrected *p* = 3.56e–10) (Figure 1F).

### 2.3 Uneven Distribution of rep‐SVs Between the X Chromosomes and Autosomes

Sex chromosomes behave allocyclically (characterized by altered condensation cycles) during spermatogenesis [41], which shall influence the transposition success between sex chromosomes and autosomes. Additionally, differences in recombination rates between sex chromosomes and autosomes may further influence the retention of newly occurred SVs, particularly the larger ones [1]. We thus investigated potential distributional discrepancies in rep‐SVs and nonrep‐SVs between the X chromosome and autosomes. The Y chromosome was excluded due to uncertainties stemming from incomplete assembly and low assembly quality.

**FIGURE 2:**
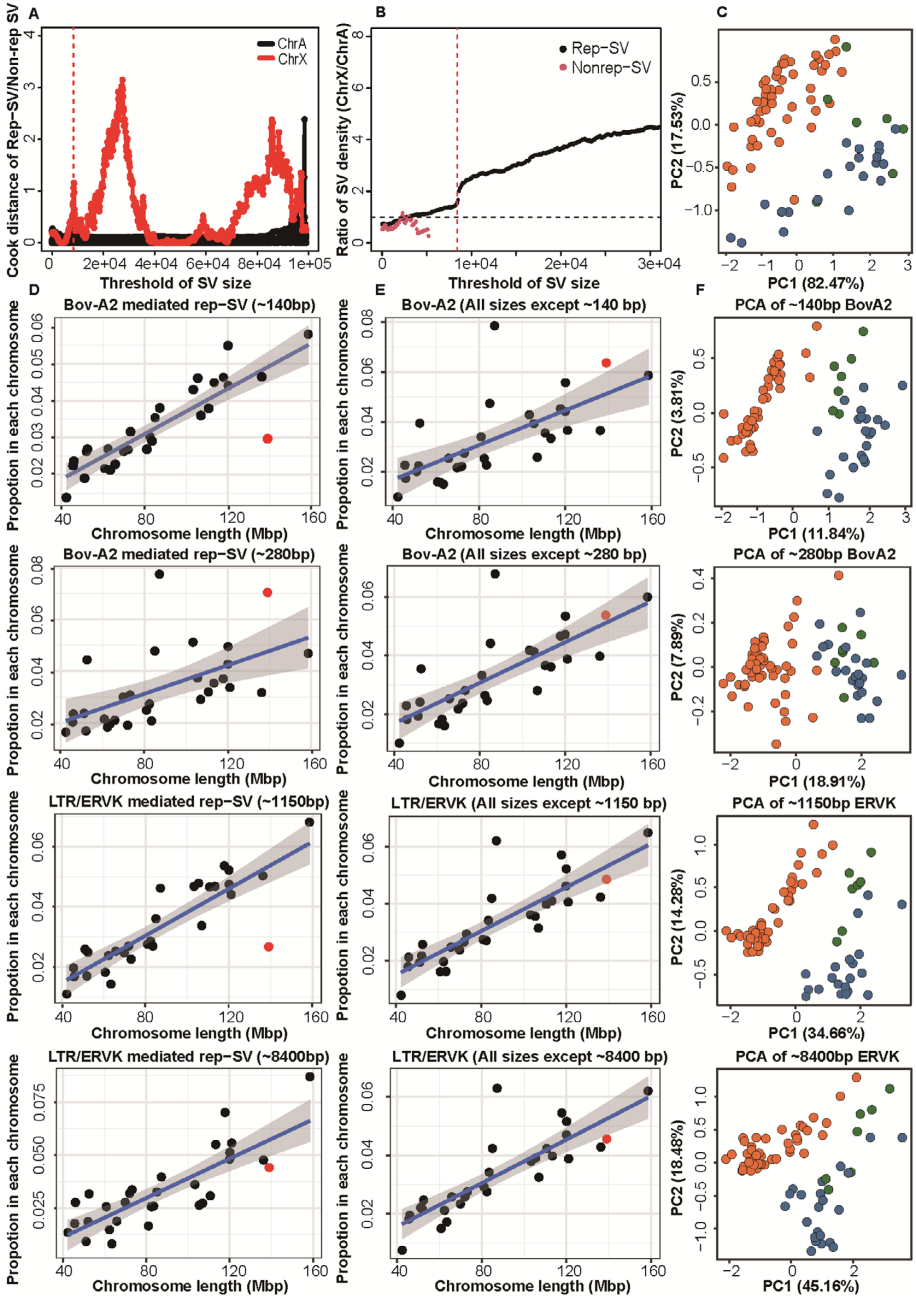
Uneven distribution of rep‐SVs between the X chromosome and autosomes. (A), Cook distance for the count ratio of rep‐SV to nonrep‐SV across chromosomes, calculated under varying SV size thresholds. Red dots represented the X chromosome, and black dots represented autosomes. (B), Ratio of SV density on the X chromosome to that on autosomes across different SV size thresholds. Black dots denoted rep‐SVs, and red dots denoted nonrep‐SVs. The black dashed line (ratio = 1) indicated equal density between the X chromosome and autosomes; the red dashed line marked the 8000 bp SV size threshold. (C), PCA analysis of individual‐specific density ratios of X‐linked rep‐SVs, stratified by size (>8000 vs <8000 bp). (D), Linear regression of chromosome length against the proportion of SVs (with defined sizes) mediated by specific REP types across chromosomes (top to bottom: ∼140 bp Bov‐A2, ∼280 bp Bov‐A2, ∼1150 bp LTR/ERVK, and ∼8400 bp LTR/ERVK). Black dots represented autosomes, and red dots denoted the X chromosome. (E), Linear regression of chromosome length against the proportion of SVs mediated by specific REP types (excluding those in panel D) across chromosomes. Black dots represented autosomes, and red dots denoted the X chromosome. (F), PCA of individual‐specific frequencies of specific REP types with defined sizes versus other sizes (top to bottom: ∼140 bp Bov‐A2, ∼280 bp Bov‐A2, ∼1150 bp LTR/ERVK, and ∼8400 bp LTR/ERVK).

Overall, the counts of rep‐SVs and nonrep‐SVs per chromosome were linearly correlated (linear model, R^2^ = 0.8697, *p* = 6.456e–14). As SV size increased, the per‐chromosome count ratio of rep‐SVs to nonrep‐SVs on the X chromosome deviated increasingly from that on autosomes (Figure 2A). This deviation arose from drastically higher relative densities of large rep‐SVs on the X chromosome versus autosomes (density‐X/density‐A), with a sharp increase in rep‐SVs over 8000 bp (Figure 2B).

This X‐biased distribution of large rep‐SVs was consistent across individuals, though not statistically significant in 9 samples — 7 of which were from Hainan Island, China (Grubbs test, *p* = 5.16e–08∼0.498; Figure S6). Notably, young rep‐SVs >8000 bp did not exhibit this X bias (Figure S7), suggesting the X chromosome preferentially retains large rep‐SVs over long evolutionary timescales. We further analyzed the ratio of X‐chromosome rep‐SVs longer than 8000 bp to shorter than 8000 bp via principal component analysis (PCA), and observed clear separation between humpless taurine and humped indicine (Figure 2C).

Since large rep‐SVs displayed unequal chromosomal distribution, we further explored whether specific REP types with distinct SV sizes show biased chromosomal distribution. Notably, Bov‐A2‐mediated rep‐SVs of ∼140‐bp size were significantly depleted on the X chromosome compared to Bov‐A2‐mediated rep‐SVs with other sizes (linear model, Bonferroni *p* = 0.029; Figure 2D,E). This X chromosome deficiency was also observed in LTR/ERVK‐mediated rep‐SVs of ∼1150‐bp size relative to other sizes (linear model, Bonferroni *p* = 0.0018; Figure 2D,E). In contrast, Bov‐A2‐mediated rep‐SVs of ∼280‐bp size and LTR/ERVK‐mediated rep‐SVs of ∼8400‐bp size showed no chromosomal distribution bias (Figure 2D,E).

This X‐chromosome depletion may be linked to evolutionary history. For the two size peaks with X bias, the first principal component (PC1) of individual proportions of the Bov‐A2‐mediated rep‐SVs peak with ∼140‐bp and other sizes clearly separated taurine from indicine (Figure 2F), which was consistently observed for the ∼1150‐bp LTR/ERVK peak. Conversely, PC1 for the two unbiased peaks (∼280‐bp Bov‐A2 and ∼8400‐bp LTR/ERVK) failed to distinguish taurine from indicine (Figure 2F).

### 2.4 Distinct Community Ecology of rep‐SVs Across Cattle Evolutionary History

Measuring rep‐SV dynamics in terms of community ecology is becoming an increasingly useful avenue of inquiry [42]. Building on this, we evaluated rep‐SV diversity across individuals using two kinds of indices: the alpha diversity (including Shannon diversity index and Pielou's J evenness) and the beta diversity. To refine our analysis, we categorized rep‐SVs into three evolutionary states: subspecies‑shared rep‐SVs that were shared by at least one taurine and one indicine individual; subspecies‑private rep‐SVs that were shared by at least two individuals within one subspecies but absent in the other; and specific rep‐SVs that appeared only in a single individual.

**FIGURE 3:**
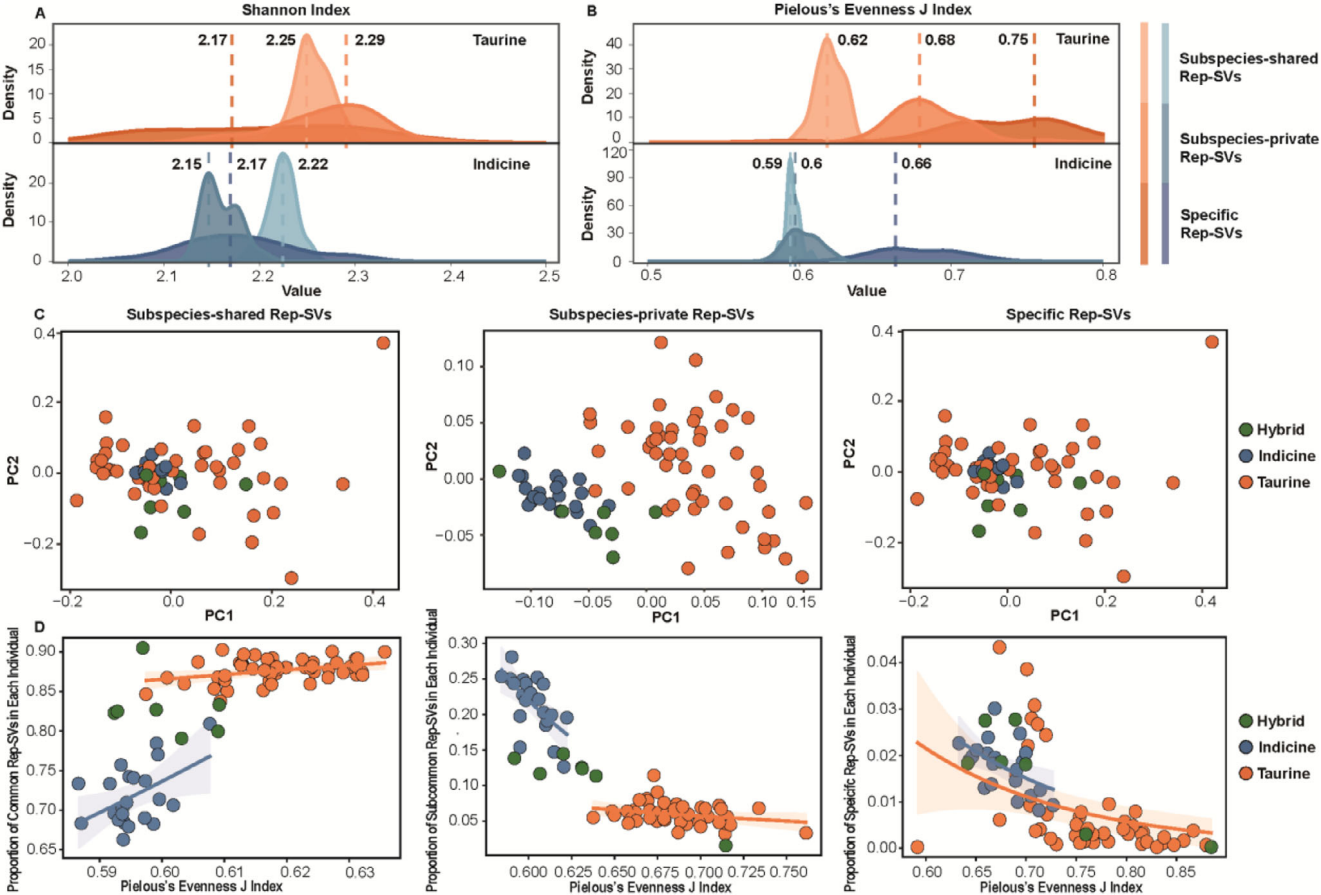
Ecological characteristics of rep‐SVs during cattle evolutionary history. (A), Density distribution of Shannon diversity indices for subspecies‐shared rep‐SVs, subspecies‐private rep‐SVs, and specific rep‐SVs (with intensified color) across all 83 individuals. (B), Density distribution of Pielou's J Evenness indicies for subspecies‐shared rep‐SVs, subspecies‐private rep‐SVs, and specific rep‐SVs (with intensified color) across all 83 individuals. (C), Beta diversity of the 83 individuals based on subspecies‐shared rep‐SVs, subspecies‐private rep‐SVs, and specific rep‐SVs, visualized via PCA with Bray–Curti distance. (D), Relationship between REP content and Pielou's J Evenness, stratified by subspecies. The solid lines represented the linear regression fits, with shaded areas indicating 95% confidence intervals. Orange dots denoted taurine, Blue dots denoted indicine, and green dots denoted hybrid.

Shannon diversity, which quantifies overall REP type diversity per assembly, increased progressively from ancient (subspecies‑shared) to recent (specific) rep‐SVs (Figure 3A). This pattern was consistently observed for Pielou's J (a measure of evenness, accounting for REP type relative abundance; Figure 3B). Noticeably, in indicine, Shannon diversity of subspecies‑private rep‐SVs was apparently lower than that of subspecies‑shared and specific rep‐SVs (wilcox test, *p* = 4.77e–07 and 0.1, respectively), a pattern not seen in taurine. This reduction coincided with a sharp decrease in the evenness of subspecies‑private repSVs in indicine, whereas evenness remained stable in taurine. These results indicated that during subspeciation, indicine accumulated a narrow range of unique REP types, distinct from broader accumulation in taurine.

We next assessed beta diversity via PCA with Bray–Curtis distance, a metric that weights the most abundant REP types and quantifies inter‐assembly diversity. Beta diversity based solely on subspecies‑private rep‐SVs clearly separated humped indicine from humpless taurine; this separation was not achieved with subspecies‑shared or specific rep‐SVs (Figure 3C). For subspecies‑private rep‐SVs, indicine also exhibited a clustered distribution compared to the dispersed distribution of taurine, further confirming more homogeneous dynamics of rep‐SVs in indicine during subspeciation.

A downward trend of evenness in relation to recent TE content among mammalian species indicated the accumulation pattern of individual TE types rather than multiple TE types at any given period [42]. We extended this analysis to rep‐SVs representing distinct evolutionary timescales. In indicine, Pielou's J showed a significantly negative correlation with increasing subspecies‑private rep‐SV content (Figure 3D). However, this trend was weaker/absent for subspecies‑shared rep‐SVs and subspecies‑private rep‐SVs in taurine, indicating an accumulation bias in indicine genomes: preferential retention of fewer REP types during subspeciation. Notably, this negative correlation also appeared for specific rep‐SVs in both subspecies (Figure 3D), suggesting biased accumulation of individual REP type might shape cattle breed formation.

### 2.5 Composition of rep‐SVs Following Cattle Evolutionary History

**FIGURE 4:**
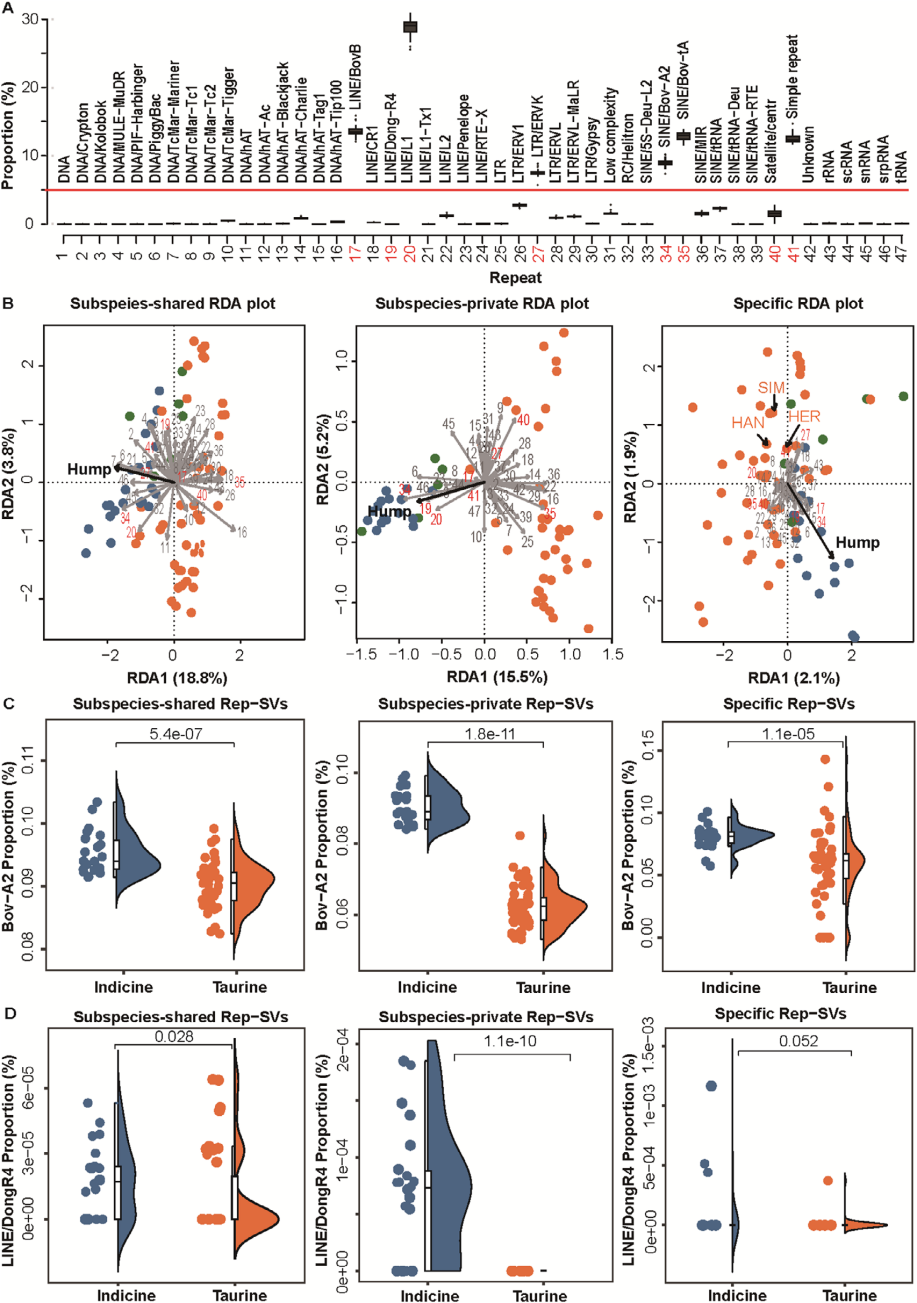
Composition of rep‐SVs and their contributions to cattle evolution. (A), Genome‐wide proportion of each rep‐SV type across the 83 individuals. (B), RDA showing major axes of variation in rep‐SV accumulation related to subspecies or breed affiliation, based on subspecies‐shared rep‐SVs, subspecies‐private rep‐SVs, and specific rep‐SVs (left to right). Percentages accounted for the variation of rep‐SV accumulation in each of the first two axes. Arrow length reflected the correlation strength of rep‐SV types with the RDA axes, where each axis represented changes in rep‐SV composition related to breed or subspecies affiliation. Orange, blue, and green points denoted taurine, indicince, and hybrid breeds, respectively. Numbers corresponded to the same rep‐SV types as in (A). Red numbers highlighted two categories of rep‐SVs: those with proportion >5%, and additional key types (LINE/Dong‐R4 and satellite/centr). SIM: Simmental cattle; HAN: Hanwoo cattle; HER: Hereford cattle. (C), Proportion of Bov‐A2‐mediated rep‐SVs in each category (subspecies‐shared, subspecies‐private, specific). (D), Proportion of satellite‐mediated rep‐SVs in each category (subspecies‐shared, subspecies‐private, specific).

Since the characterization of rep‐SV dynamics indicated rapid expansion of individual REP types rather than multiple REP types in indicine during subspeciation, we further investigated which REP types underwent intense accumulation across distinct evolutionary timescales. Overall, the proportions of SVs mediated by each REP type were consistent across the 83 individuals, whereas six REP types were pervasive (accounting for >5% of total rep‐SVs) and exhibited marked variance among individuals, including BovB, Bov‐A2, Bov‐tA, LINE/L1, LTR/ERVK, and simple repeat (Figure 4A). Among these, L1 showed the most pronounced inter‐individual differences in proportion.

To dissect the variance of REP composition more closely, we conducted a redundancy analysis (RDA) separately for the subspecies‐shared, subspecies‐private, and specific rep‐SV categories. This analysis aimed to identify the major axes of variation in REP composition related to aurochs polymorphism, subspecies divergence, and breed affiliation (Figure 4B). Consistent with the negative correlation between evenness and rep‐SV content, RDA revealed a strong phylogenetic component to variation in REP composition separately at the auroch population, subspecies, and breed levels, suggesting a potential difference in REP accumulation since the ancestral auroch state. In general, the pervasive REP types (>5% proportion) displayed stage‐specific relationships with certain subspecies or breeds, except Bov‐A2 [43], a REP type derived from BovB [26], which maintained a consistent association with the hump trait across hierarchical evolutionary scales. Beyond these pervasive REP types, LINE/Dong‐R4 was mostly related to the indicine during the sub‐speciation process (Figure 4B).

To assess whether the frequency of subspecies‐private rep‐SVs mediated by Bov‐A2 and Dong/R4 differed significantly between taurine and indicine, we retrieved 397 available genomes with short‐read sequencing data, yielding 206 336 SVs. These genomes encompassed 175 taurine individuals from 18 breeds, 67 individuals from 8 hybrid breeds, and 155 indicine individuals from 16 breeds. While Bov‐A2‐mediated rep‐SVs were pervasive in both subspecies across the subspecies‐shared, subspecies‐private, and specific groups, it differed mostly in the subspecies‐private group, with a pronounced higher proportion in indicine (wilcox test, *p* = 1.6e–11; Figure 4C). Parallely, LINE/Dong‐R4 also exhibited a significantly higher proportion in indicine during the subspeciation process (wilcox test, *p* = 1.1e–10; Figure 4D).

Among these Bov‐A2‐mediated rep‐SVs, one located within the *Platelet‐Derived Growth Factor D* (*PDGFD*) gene exhibited an extreme frequency difference between taurine and indicine (*F*_ST_ = 0.56). Overexpression of *PDGFD* has been reported to potentially affect fat tail development in sheep, as it is involved in fat deposition [43]. Given the distinct difference in hump trait between taurine and indicine, along with higher intermuscular fat content in the hump compared to other skeletal muscles, we further investigated *PDGFD* expression profiles in nine tissues of the Bactrian camel (PRJNA857334). The gene showed the highest expression level in the hump (Figure S8), suggesting *PDGFD* might be associated with the hump trait in indicine. To validate this, we examined *PDGFD* expression in hump tissues of Chinese yellow cattle (a Yunnan indicine breed), comparing four castrate males with smaller humps and three normal males with larger humps. *PDGFD* tended to be expressed much higher in larger humps (Figure S9A). Notably, hump size in indicine cattle is strongly influenced by castration, indicating a close association with endocrine regulation. Given that gonadal tissues are central to hormone production and that reproductive isolation represents a key driver of speciation, we further examined *PDGFD* expression in testis tissues. *PDGFD* was highly expressed in castrated testis compared with normal testis from the same individuals for hump comparison (student *t*‐test, *p* = 0.04; Figure S9B) and showed marginally higher expression in pure taurine than in pure indicine cattle (student *t*‐test, *p* = 0.061; Figure S9C). Taken together, the concordantly diverged expression patterns of *PDGFD* observed in both hump and testis tissues suggest that *PDGFD* may be responsive to endocrine status and may participate in regulatory pathways linking reproductive physiology with subspecies‐defining morphological traits such as hump size. Nevertheless, additional samples and in‐depth functional experiments will be required to validate this trend.

### 2.6 A Satellite‐Mediated rep‐SV With High Divergence in Beef Breeds

**FIGURE 5:**
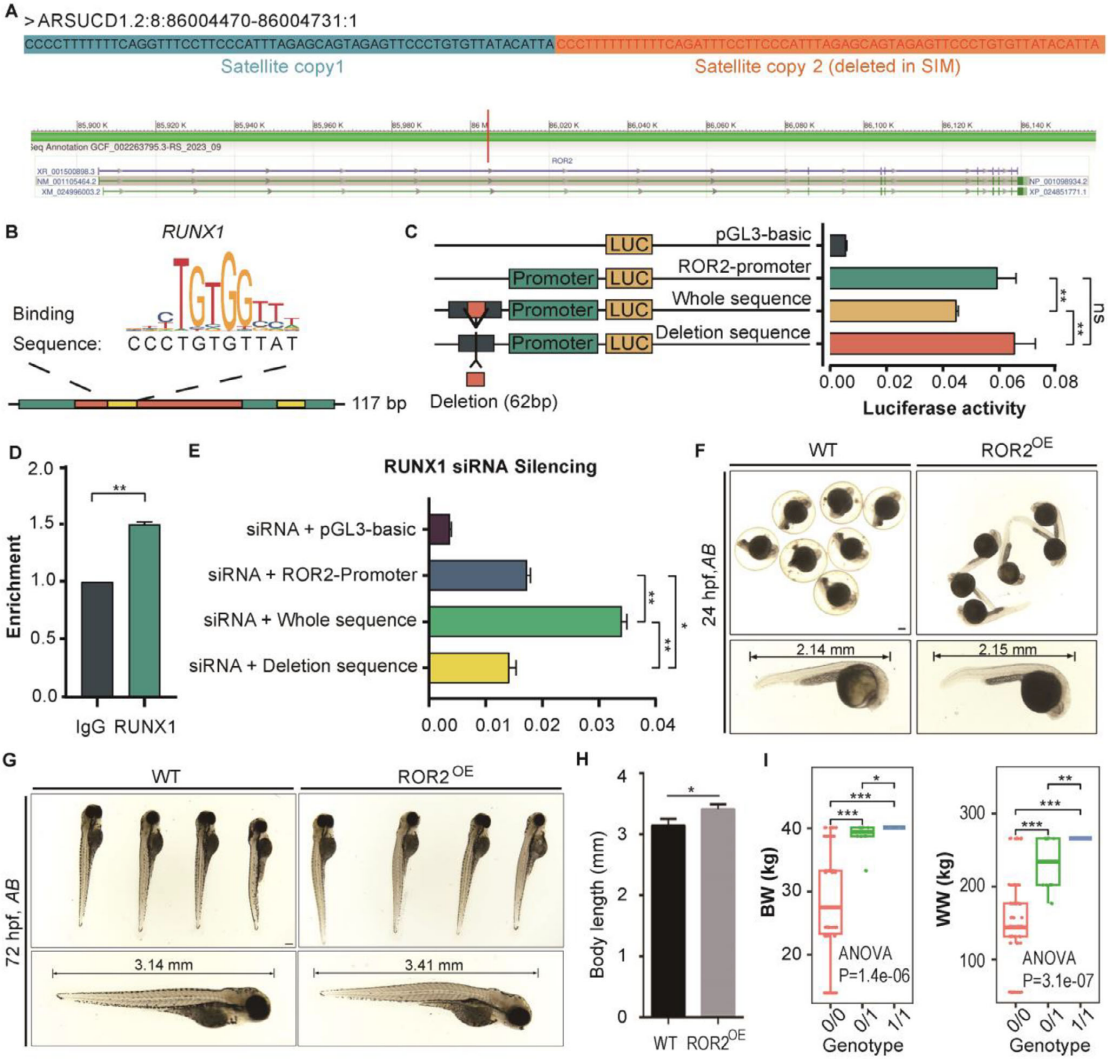
Functional validation of a candidate rep‐SV in *ROR2*. (A), The rep‐SV structure within intron1‐2 of *ROR2*. (B), Position weight matrix (PWM) of the RUNX1 transcription factor binding site within the 62‐bp deletion region, retrieved from the JASPAR database. Green band: predicted core transcription factor binding region (117 bp); Orange band: 62‐bp deletion fragment; Yellow band: transcription factor binding sequences. (C), Dual‐luciferase reporter assay in transiently transfected MDBK cells. Ordinate: relative luciferase activity (firefly luciferase/Renilla luciferase ratio) of four plasmid vectors: pGL3‐basic (blank control); ROR2‐Promoter (pGL3‐basic vector with insertion of the *ROR2* promoter region, indicated by the green band); Whole sequence (vector containing the full reference sequence); Deletion sequence (vector harboring the 62‐bp deletion fragment, indicated by the orange band). Data represented six biological replicates. (D), RUNX1 binding preference to the 62‐bp deletion fragment in MDBK cells, detected by chromatin immunoprecipitation (ChIP). Abscissa: target protein (RUNX1 or nonimmune IgG as negative control); Ordinate: relative enrichment of target DNA fragments. Data represented three biological replicates. Error bars denoted standard deviation (SD); *p* values were calculated using a two‐tailed *t*‐test (∗∗*p* < 0.01). (E), Quantitative regulation of *ROR2* expression via RUNX1 binding efficiency. Dual‐luciferase reporter assay in MDBK cells co‐transfected with the four plasmids (from panel C) and RUNX1‐targeting siRNA. Data represented six biological replicates. (F), Zebrafish phenotypes at 24 h post‐fertilization (hpf). (G), Zebrafish phenotypes at 72 hpf. (H) Statistical analysis of zebrafish body length (*n* = 10). Error bars denoted SD; *p* values were calculated with a two‐tailed *t*‐test (^*^ *p* < 0.05, ^**^ *p* < 0.01). (I) Association between the satellite‐mediated rep‐SV genotype and phenotypic traits across taurine breeds. Taurine breeds included Angus, Hanwoo, Holstein, Hereford, Jersey, Simmental, Luxi, Nanyang, and Tibetan cattle. Phenotypic traits: BW (birth weight), WW (weaning weight). Significance between genotypes was determined by a two‐tailed *t*‐test; significance between genotype and phenotype was determined by one‐way ANOVA.

We next focused on the potential role of rep‐SVs highly diverged among breeds. Notably, satellite sequences exhibit a clear association with several beef cattle breeds, as illustrated by representative intensively selected beef breeds such as Simmental, Hereford, and Hanwoo in our analysis (Figure 4B). To explore the potential contribution of satellites to beef breed formation, we focused on a DEL in the *Receptor Tyrosine Kinase Like Orphan Receptor* (*ROR2*) gene intron 1/2, mediated by a ∼62‐bp satellite unit (Figure 5A). This derived rep‐SV was most frequent in SIM (beyond Top‐1% *F*_ST_), and moderately frequent in other taurine beef breeds (e.g., Holstein, Hereford, Manie Anjou, and Gelbvieh), all of which exhibit a large body frame and excellent muscular development.

*ROR2* is a receptor for Wnt5a, a signaling molecule essential for embryonic development [44]. Disruption of *ROR2* induces foreshortened or malformed bones in mouse embryos [45]. We found that this satellite‐mediated‐rep‐SV reduced the number of binding sites for the transcription factor RUNX1 from two to one (Figure 5B). RUNX1 is known to regulate skeletal phenotype [46], prompting further interrogation of this DEL using the bovine MDBK cell line. Dual‐luciferase reporter assay clearly identified that this DEL significantly increased luciferase activity relative to the wild type (Fold change = 1.44, *p* = 0.008) (Figure 5C), indicating that the DEL allele is associated with enhanced *ROR2* expression. We then addressed whether this upregulation was due to a reduced binding efficiency of RUNX1 within this DEL region. ChIP‐qPCR assay first validated significant enrichment of RUNX1 at the 62‐bp fragment, compared to the IgG negative control (*p* < 0.01; Figure 5D), confirming direct binding and implying that the DEL would diminish RUNX1 occupancy at this locus. We next tested whether RUNX1 negatively regulates *ROR2* using siRNA‐mediated RUNX1 knockdown. This siRNA achieved ∼70.27% knockdown efficacy specific to RUNX1 (*p* < 0.001; Figure S10). Subsequent expression analysis showed that cells carrying the wild‐type allele (two RUNX1 binding sites) had significantly higher *ROR2* expression than those with the DEL allele (one binding site) (Figure 5E), directly validating RUNX1's negative regulatory role on *ROR2*.

To investigate the potential phenotypic effects of *ROR2* upregulation, we microinjected bovine *ROR2* mRNA into one‐cell‐stage fertilized zebrafish (*Danio rerio*) embryos (*ROR2* ^OE^). Embryos in the *ROR2* ^OE^ group typically dechorionated much earlier (∼24 h post‐fertilization) than wild‐type embryos (∼48 h post‐fertilization) (Figure 5F), though no differences of body length were observed at this stage. However, at 72 h post‐fertilization, the average body length of the *ROR2* ^OE^ embryos was significantly longer than that of wild‐type embryos (Figure 5G,H).

Given that taurine beef breeds can be fattened intensively to high weights [47, 48], we further examined whether body size and growth traits varied with genotypes of this *ROR2* rep‐SV among taurine individuals, via univariate analysis of variance (ANOVA). Consistent with the zebrafish experiments, birth weight and weaning weight were significantly associated with the rep‐SV genotypes (*p* = 1.41e–6, 3.05e–7, and 5.01e–7, respectively; Figure 5I). These results should be interpreted cautiously, however, as individual variation, climate, husbandry practices, and feeding conditions may also influence these traits. Collectively, these findings suggested that the 62‐bp rep‐SV in *ROR2* may contribute to the large body size of taurine beef breeds and holds potential for application in future molecular breeding programs.

## 3 Discussion

REPs likely play a pivotal role in animal domestication and breed formation. For example, a 2,809‐bp LINE‐1 insertion in *ASIP* has been associated with the white coat phenotype in swamp buffalo [49], and a SINE insertion in the 3′‐UTR of *MYO5A* in European pigs has been shown to influence alternative splicing and coat color variation [50]. Despite these examples, the overall evolutionary dynamics of REPs in domestication‐related processes remain underexplored [51, 52, 53]. To address this gap, we conducted a systematic assessment of REP content across 83 long‐read sequencing individuals from domesticated cattle, encompassing both taurine and indicine subspecies. A key advantage of our approach is the use of long‐read data to construct a sequence‐resolved bovine panSV: we identified ∼33.15% novel SVs compared to a previous cattle SV catalog derived mostly from short‐read sequencing data, with a collected exhaustive sample size—highlighting the critical role of long‐read data in capturing SV diversity.

Our research explored the evolutionary characteristics of various types of rep‐SVs. Instead of focusing solely on sequence variation, this investigation considered genomic distribution, community dynamics, and ecological composition. Our data revealed that different rep‐SVs evolve in distinct ways, leaving lasting legacies over evolutionary timescales. We explored these patterns from multiple perspectives.

First, different rep‐SVs exhibited striking divergence in distribution between the X chromosome and autosomes, accompanying with varied rep‐SV sizes. Large rep‐SVs (>8000 bp) were proportionally enriched on the X chromosome (Figure 2B). One plausible explanation is X‐inactivation, which preferentially silences abnormal X chromosomes [54]. As a result, large SVs have a greater chance to persist as polymorphisms without severe fitness costs. However, this mechanism cannot fully explain the pattern, as a parallel enrichment of chromosomal rearrangements on the X chromosome has been observed in Drosophila, a species lacking X‐inactivation [55]. An alternative, more generalizable driver is ectopic recombination, which predominantly affects TE fixation in various species [56]. Longer TEs could be strongly selected against due to their higher likelihood of initiating recombination. Consequently, large SVs tend to accumulate in genomic regions with low recombination rates, e.g., the X chromosome. This recombination‐dependent elimination would require long evolutionary timescales, as supported by the absence of X‐chromosome bias in young large rep‐SVs (>8000 bp).

In contrast to large rep‐SVs, two kinds of short rep‐SVs—Bov‐A2‐mediated ∼140‐bp rep‐SVs and LTR/ERVK‐mediated ∼1150‐bp rep‐SVs—were depleted on the X chromosome. Bov‐A2‐mediated ∼280‐bp rep‐SVs were accumulated on the X chromosome, while simple repeat‐mediated ∼8400‐bp rep‐SVs showed balanced distribution between the autosome and the X chromosome. Notably, the abundance of rep‐SVs with pronounced distribution bias often exhibited divergence between the two subspecies, suggesting that taurine and indicine ancestors evolved distinct strategies to suppress REP mobility, with lineage‐specific differences in how short rep‐SVs are fixed on the X chromosome.

Second, beyond size and chromosomal distribution, we leveraged ecological communities (e.g., Shannon diversity, Pielou's J evenness, and beta diversity) to characterize rep‐SV dynamics across varied evolutionary timescales, particularly the subspeciation period. Indicine exhibited a subspecies‐specific reduction in Shannon diversity and Pielou's J evenness for subspecies‐private rep‐SVs (Figure 3A,B), indicating that indicine other than taurine accumulated a small subset instead of a broad range of REPs during the subspeciation process. This signature was further supported by two lines of evidence: (1) centered beta diversity in indicine revealed distinct rep‐SV community structures between subspecies (Figure 3C); (2) indicine had higher genomic content of subspecies‐private rep‐SVs with lower Pielou's evenness (Figure 3D). Since this peculiar community pattern was absent in taurine, it is reasonable to hypothesize that subspecies divergence in cattle might be driven by asymmetric rep‐SV dynamics, where rapid expansion of individual REP types in indicine under lineage‐specific homogeneous selection.

Third, ecological composition analysis via RDA identified Bov‐A2 and LINE/Dong‐R4 as the primary drivers of rep‐SV expansion in indicine during the subspeciation process (Figure 4). Most other REP types showed variable associations with different individuals, suggesting that evolutionary pressures on REP expansion and host defenses against REPs, have diverged across populations over time. This pattern was parallely observed in a previous study [17]. Notably, Bov‐A2 and LINE/Dong‐R4 might contribute to the subspeciation process as their frequencies in subspecies‐private rep‐SVs were highly differentiated between indicine and taurine. Notably, a Bov‐A2‐mediated rep‐SV mapped to the *PDGFD* gene, which is involved in adipose metabolism [43]. This gene's expression in camel humps (Figure S8) and its higher expression in larger humps of Yunnan indicine (Figure S9) link it to the hump trait—an iconic adaptation of indicine cattle.

Following this avenue of investigating the functional changes of rep‐SVs according to their evolutionary dynamics, we further identified a satellite‐mediated 62‐bp deletion in *ROR2* that is highly frequent in beef cattle. This deletion reduces binding sites for the transcript factor RUNX1 (a regulator of skeletal development [46]), leading to upregulated *ROR2* expression (Figure 5E). Functional validation in zebrafish (longer body length in *ROR2*‐overexpressing embryos) and cattle population analysis (associations with birth weight and weaning weight) confirmed a link between this rep‐SV and large body size—an economically critical trait in beef cattle. These results position such highly diverged rep‐SVs as promising candidates for breed certification markers and molecular breeding applications.

Despite the strengths of our study, several limitations should be considered. First, while our cross‐platform comparison indicated a limited influence of sequencing platform on cattle SV detection, we acknowledge that platform‐specific technical biases may still exist. Future studies incorporating multi‐platform sequencing or standardized assembly quality assessments could further validate and generalize these findings. Moreover, employing the taurine reference genome ARS‐UCD1.2 can introduce bias in SV detection between indicine and taurine, likely contributing to the higher number of SVs identified in indicine individuals (Table S5). Although this bias does not substantially affect our primary conclusions regarding chromosomal distribution and differences among REP types, future studies using indicine‐specific reference genomes or pangenome‐based approaches will help minimize this bias, refine SV detection, and provide a more precise landscape of REP dynamics in bovines. Second, we used a 1‐bp overlap criterion to define rep‐SVs, aligning with previous work [39, 40]. However, this definition may introduce false positives for large SVs, which are more likely to overlap REPs by chance. To mitigate this, we tested an alternative, mechanism‐based definition (SVs with REPs overlapping within a 10‐bp window of breakpoints); consistent evolutionary patterns across both definitions confirm the robustness of our conclusions (data not shown here). Further refinement of rep‐SV definitions (e.g., incorporating REP‐mediated breakage signatures) is needed. Third, the unbalanced number of samples across breeds may have biased the RDA‐based association between rep‐SVs and breed identity. Future studies with balanced, larger cohorts will improve the reliability of such an association. Fourth, while we linked *PDGFD* and *ROR2* to phenotypic traits, in vivo functional validation for cattle (e.g., CRISPR‐Cas9‐mediated knockout/knock‐in) is required to confirm their causal roles in subspecies divergence and trait evolution. Finally, for *ROR2*, detailed data on individual cattle genotypes and corresponding body size measurements (e.g., height, weight at multiple time points) would clarify the exact contribution of this rep‐SV to growth traits.

## 4 Conclusion

Examining long‐read sequencing data of 83 globally distributed cattle identified 69 295 novel SVs, a critical resource for resolving cattle pan‐genome diversity. Community dynamics of rep‐SVs clearly revealed that specific expansions in indicine might contribute to the subspeciation process. Collectively, rep‐SVs underlie cattle subspecies divergence and breed trait evolution, providing candidate markers for molecular breeding and evolutionary genetics research.

## 5 Experimental Section

### 5.1 Sample Collection and Sequencing

We sampled blood from Angus, Simmental, Yanbian cattle, Muturu, Kuri, Iran cattle, Brahman, and Iran zebu for Oxford Nanopore sequencing, and the detailed information of the samples is in Table S1.

Genomic DNA was extracted from the tissues using the phenol/chloroform method. The Nanodrop, Qubit, and agarose gel electrophoresis were used for quality assessment of DNA. The fragments were size‐selected with Blue Pippin. Nanopore libraries were constructed using the Ligation Sequencing Kit (SQK‐LSK109) and sequenced on Nanopore PromethION48 flow cells. Base calling was performed using Guppy (v.5.1.13).

### 5.2 Small Variants Calling and Population Structure Analyses

Raw Illumina reads were processed using Trimmomatic v0.39 [57] to remove adapters and low‐quality sequences. All clean reads were mapped to the ARS‐UCD1.2 reference genome using BWA‐MEM 0.7.12‐r1039 [58] with default parameters. Multi‐mapped and unmapped reads were discarded using SAMtools v1.3.1 [59]. The Picard package (http://broadinstitute.github.io/picard) was used for duplicate filtration. SNPs were called from bam files using the command HaplotypeCaller and genotyped using the command GenotypeGVCFs implemented in Genome Analysis Toolkit v4.2.2.0 (GATK v4.2.2.0). SNPs and InDels were extracted using SelectVariants. The VariantFiltration method was used for hard‐filtering with the parameters ‘QD < 2.0 || FS > 60.0 || MQ < 40.0 || SOR > 3.0 || MQRankSum < −12.5 || ReadPosRankSum < −8.0’ for SNPs and ‘QD < 2.0 || FS > 200.0 || SOR > 10.0 || MQRankSum < −12.5 || ReadPosRankSum < −20.0’ for InDels.

For further analysis, we removed those with >10% missing data, <0.05 minor allele frequencies, or that failed the Hardy–Weinberg equilibrium (*p* <1.0–6). For phylogenetic analysis, we constructed a Neighbor–Joining tree using rapidNJ [60] and visualized it using FigTree v1.4.4 (http://tree.bio.ed.ac.uk/software/figtree/). For PCA, we used ‘‐pca’ option in PLINK v1.9 [61]. For admixture analysis, we pruned the genotype data based on linkage disequilibrium with ‘‐indep‐pairwise 50 10 0.1’ option as recommended in PLINK v1.9 [61]. Admixture v1.3.0 [62] was conducted with K ranging from 2 to 3, where K is the assumed number of ancestral populations.

### 5.3 SV Detection and Annotation

Our integrated long‐read sequencing data were derived from two sequencing platforms: Nanopore and Pacbio (Table S3). We aligned long‐read sequencing data to the reference genome ARS‐UCD1.2 for each sample, using Winnowmap v2.03 [63] with the option ‘‐ax map‐ont’ for ONT reads, and the option ‚map‐hifi‘ for HIFI reads. Alignments were sorted and indexed using SAMtools v1.3.1 [59]. SVs were detected for each individual using cuteSV v1.0.11 [64], SVIM v2.0.0, and Sniffles2 v2.0.7. For ONT reads, we set the following parameters for cuteSV:“ –max_cluster_bias_INS 100 –diff_ratio_merging_INS 0.3 –max_cluster_bias_DEL 100 –diff_ratio_merging_DEL 0.3 –min_size 50 –max_size 500000 –genotype –report_readid sample”. For HIFI reads, we set the following parameters for cuteSV:“ –max_cluster_bias_INS 1000 –diff_ratio_merging_INS 0.9 –max_cluster_bias_DEL 1000 –diff_ratio_merging_DEL 0.5 –min_size 50 –max_size 500000 –genotype –report_readid sample”. We set the following parameters for SVIM:“—min_sv_size 50 –insertion_sequence –symbolic_alleles –read‐names –sample”. We set the following parameters for Sniffles:“—minsupport 5 –minsvlen 50 –sample‐id”. Then we removed positions marked as IMPRECISE for INFO and as q5 for QUAL and excluded sites of missing data. We merged all VCF files to yield a total SV dataset using SURVIVOR v1.0.3 [65] with parameters ‘1000 2 1 ‐1 ‐1 50’ to merge the SVs (maximum allowed pairwise distance of 1000 bp between breakpoints, two or three calling methods support, types must match, a minimum SV length of 50 bp). We removed SVs larger than 100 kbp and filtered out unreliable genotypes (all 0/0), yielding a total of 209 032 nonredundant panSV datasets. No statistically significant differences in the overall SV detection efficiency were observed between Nanopore and Pacbio platforms (Figure. S11). Key quality metrics, including N50 contig length for all 83 datasets, were detailed in Tables S2 and S3. The N50 contig lengths of the 83 datasets ranged from 11 197 to 63 993 bp, ensuring sufficient sequence continuity for direct SV calling. To assess potential miss calling influenced by sex (e.g., twice the X‐chromosomes coverage in females), we assessed the correlation between sequencing depth and the number of detected SVs on the X chromosome for each sample (Figure S1).

We extracted sequences of insertion and deletion from the panSVs dataset, and all SV sequences were aligned against Bos taurus’ consensus repeat sequences by RepeatMasker v4.1.5 (http://www.repeatmasker.org) to determine if they are transposable elements. We used ANNOVAR [66] to annotate SVs by their distance with protein‐coding genes. We used a subcommand of BEDtools with “intersect ‐a panSV.bed –b published_SV.bed –wa –wb –r –f 0.5” to detect novel SVs, which were deemed to have never been reported if the SV in panSV had less than 50% reciprocal overlap with published SV sets. For comparison of INS, the starting position of INS plus the insertion length was designated as the terminating coordinate of the INS. To investigate whether the types of repetitive sequences mediating SV differed across distinct evolutionary scales, we isolated SVs that occur in only one individual and defined them as young SVs. To assess sample size saturation, we performed in silico simulations to determine whether increasing the number of samples would no longer substantially alter the total number of SVs or the count of core SVs.

These 209 032 nonredundant SVs were genotyped in 397 cattle and zebu that had been sequenced with short‐read sequencing data with a minimum depth of 8x (Table S4). We mapped the short‐read sequencing data to the reference genome ARS‐UCD1.2, using BWA‐MEM [58] with default parameters. Then, SVs were genotyped for all samples using Paragraph [67] and integrated via BCFtools v1.10.2 [68]. SVs with a >50% missing rate among all individuals were excluded for further analysis, and we obtained a total of 206 336 SVs.

We categorized rep‐SVs into three evolutionary states: subspecies‐shared rep‐SVs that were shared by at least one taurine and one indicine individual; subspecies‐private rep‐SVs that were shared by at least two individuals within one subspecies but absent in the other; and specific rep‐SVs that appeared only in a single individual, which also referred to young SVs.

### 5.4 Chromosomal Distribution of rep‐SVs and Nonrep‐SVs

The characteristics of chromosomal distribution of rep‐SVs and nonrep‐SVs were analyzed in R (https://www.r‐project.org/). To detect outliers deviating from the linear regression model, the ‘outlierTest’ function from the “car” package [69] was used. We then assessed the extent of deviation of these ratios using the local outlier factors for SVs over certain size thresholds, with the ‘cooked.distance’ function [70]. To assess whether the X chromosome possessed an abnormally high proportion of large rep‐SVs (>8000 bp), we first ran the Shapiro‐Wilk test to ensure the ratios of rep‐SVs over 8000 bp to those under 8000 bp within a chromosome were normally distributed. Then we used the ‘Grubbs.test’ function [71] from the “outliers” package to identify a single outlier among all chromosomes. Principal components analysis was performed using the ‘prcomp’ function.

### 5.5 Data Fitting Methodology

The data fitting workflow was implemented in Python v 3.13.0, leveraging the pandas v2.2.3 for data ingestion, NumPy v2.1.3 [72] for numerical computations, StatsModels v0.14.4 for fitting linear regression models, SciPy v1.14.1 [73] for nonlinear model fitting, and Matplotlib v3.9.2 for plotting. Nine distinct models, classified as either linear or nonlinear, were defined. The linear models comprised: Linear, Inverse, Logarithmic, Quadratic, Inverse and Linear, Constant and Logarithmic, and Inverse and Constant. The nonlinear models included the Exponential and Power models. Their respective functional forms are summarized in Table S6.

During model fitting, linear models were constructed using ordinary least squares (OLS) regression via statsmodels.formula.api.ols, which generates models directly from formula specifications. For models necessitating variable transformations (e.g., reciprocal, logarithm), the input data were preprocessed using a dedicated transform_data function. Parameter estimation for nonlinear models was performed using scipy.optimize.curve_fit, which identifies optimal parameters (e.g., ^*^a^*^ and ^*^b^*^ for the Exponential and Power models) by minimizing the residual sum of squares (RSS). Initial parameter guesses were provided to facilitate convergence.

Model performance was assessed using the Akaike Information Criterion (AIC), the Bayesian Information Criterion (BIC) [15], and the coefficient of determination (R^2^) to identify the optimal model. AIC evaluates the trade‐off between model complexity and goodness‐of‐fit, with lower values indicating superior models; consequently, it was the primary criterion for model selection. BIC follows a similar principle but imposes a stricter penalty on model complexity. R^2^ quantifies the proportion of variance in the dependent variable explained by the model, ranging from 0 to 1, where values closer to 1 denote a better fit. For linear models, AIC, BIC, and R^2^ were computed directly by the StatsModels package. For nonlinear models, these metrics were calculated manually based on the RSS and the number of parameters to ensure consistent comparability across all model types. The specific calculation procedures for nonlinear models are detailed below:

1) R^2^: This was computed by comparing the deviation of the model predictions from the observed values to the total deviation of the observations from their mean, as defined in Equation (1):

(1) $$R^{2}=1-\frac{SS_{res}}{SS_{tot}}$$

Here, *SS_res_* represents the residual sum of squares, i.e., the total squared difference between the observed (*y_i_*) and predicted (${\tilde{y}}_{i}$) values: $SS_{res}=\sum_{i=1}^{n}{\left(y_{i}-{\tilde{y}}_{i}\right)}^{2}$, where *n* denotes the sample size. *SS_tot_* is the total sum of squares, representing the total squared deviation of the observed values (*y_i_*) from their mean (${\tilde{y}}_{i}$): $SS_{tot}=\sum_{i=1}^{n}{\left(y_{i}-\bar{y}\right)}^{2}$.

2) AIC: The core formula, applicable to both linear and nonlinear models, is given by Equation (2):

(2) $$\mathrm{AIC}\;=2k-2\ln\left(L\right)$$

where *k* is the number of estimated parameters and *L* is the maximized value of the likelihood function. Under the assumption of normally distributed errors, AIC can be approximated using the RSS as shown in Equation (3):

(3) $$\mathrm{AIC}\;=n\cdot \ln\left(\frac{ss_{\mathrm{res}}}{n}\right)+2k$$

3) BIC: The core formula is provided in Equation (4):

(4) $$\mathrm{BIC}=-2\ln\left(L\right)+k\cdot \ln\left(n\right)$$

Assuming normally distributed residuals, BIC was approximated using the RSS as defined in Equation (5):

(5) $$\mathrm{BIC}=n\cdot \ln\left(\frac{ss_{\mathrm{res}}}{n}\right)+k\cdot \ln\left(n\right)$$

For each dataset, all candidate models were fitted, their respective performance metrics were computed, and the model with the smallest AIC value was selected as the best.

For model visualization, the fitted curve of the best model and its associated 95% confidence interval were plotted. The curve was restricted to the range of the observed independent variable data to prevent extrapolation. Confidence intervals for linear models were derived directly from the StatsModels output. For nonlinear models, confidence intervals were calculated manually using the Jacobian matrix and the t‐distribution. Specifically, the scipy.optimize.curve_fit function returns the optimal parameter estimates (popt) and their covariance matrix (pcov). The Jacobian matrix, which characterizes the sensitivity of model predictions to parameter variations, was used in conjunction with pcov via error propagation principles to estimate the standard error for each prediction point. The confidence intervals were then computed using the appropriate quantiles of the t‐distribution.

Regarding the dataset, records were classified into three subspecies categories: Taurine, Indicine, and Hybrid. The Taurine and Indicine data subsets were used for model fitting, whereas the Hybrid subset was reserved for annotating the plots.

### 5.6 Composition and Community Ecology Analysis

The characteristics of the composition and community ecology of rep‐SVs were analyzed in R (https://www.r‐project.org/). We first performed detrended correspondence analysis (DCA) to test whether the REP type compositions were suitable for redundancy analysis (RDA) or canonical correspondence analysis (CCA), using the ‘decorana’ function from the “easyCODA” package [74]. Since the axis length of DCA1 was less than 3, we chose RDA to examine the amount of variation and its statistical significance in the dependent matrix that can be accounted for by the independent matrix, using the ‘rda’ function from the “easyCODA” package [74]. The Shannon index was calculated using the ‘diversity’ function from the “vegan” package. The number of REP types (S) was calculated using the ‘specnumber’ function. Then, Pielou's J was estimated as Shannon/log(S). Beta diversity was calculated using the ‘vegdist’ function based on “bray” method.

### 5.7 Population Differentiation Analysis

397 available genomes with short‐read sequencing data were retrieved for populational genotyping frequency analyses. These genomes encompassed 175 taurine individuals (e.g., Angus, Hanwoo, Holstein, Hereford, Jersey, Simmental, Mongolian, Charolais, Finncattle, Gelbvieh, Iran taurus, Manie Anjou, Mishima, Muturu, Ndama, Tibetan, Yakutian, Yanbian), 67 hybrid individuals (e.g., Afar, Ankole, Dengchuan, Fogera, Horro, Luxi, Nanyang, Sheko), and 155 indicine individuals (e.g., Arsi, Barka, Boran, Butana, Goffa, Kenana, Lincang, Mursi, Ogaden, Red Sindhi, Thawalam, Wenshan, Hainan, Brahman, Nelore, Gir). Population differentiation (*F*_ST_) values were calculated with VCFtools v0.1.13 [75] for two datasets. Dataset 1 consisted of ten taurine populations (Angus, Apeijiaza, Hanwoo, Holstein, Hereford, Jersey, Luxi, Nanyang, Simmental, and Mongolian) and four indicine populations (Brahman, Nelore, Gir, and Yunnan yellow cattle of China). This dataset was used to estimate *F*_ST_ values between taurine and indicine populations. African populations were excluded because of their pervasive introgression between native taurine and induced indicine cattle. Dataset 2 included only taurine populations (Angus, Hanwoo, Holstein, Hereford, Jersey, Luxi, Nanyang, Simmental, and Tibetan cattle) to estimate *F*_ST_ values between individual breeds and the rest.

### 5.8 Functional Verification of Candidate rep‐SV in ROR2

According to Zhang et al. [76], bovine kidney cells (MDBK) were purchased from the Animal Germplasm Bank of the Southwest China Wild Animal Germplasm Resource Bank (Catalog ID: KCB 90031YJ). Quality control testing confirmed the absence of mycoplasma, bacterial, and fungal contamination. The cells were incubated at 37°C with 5% CO2 and grown in DMEM (Thermo Fisher Scientific, Waltham, MA, USA) supplemented with 10% FBS, 100 U/mL penicillin, and 10 mg/mL streptomycin.

Transcription factors near the rep‐SV sequence were predicted using the JASPAR database (http://jaspar.binf.ku.dk/). According to the EZ ChIP Chromatin Immunoprecipitation Kit (Millipore, Billerica, Massachusetts, USA), the enrichment of RUNX1 near the rep‐SV sequence in MDBK cells was verified by ChIP‐qPCR with a RUNX1‐specific antibody (Santa Cruz Biotechnology, Dallas, TX, USA).

For the dual‐luciferase reporter assay, the *ROR2* promoter region was cloned into the pGL3 basic vector (Promega, Madison, WI, USA). The whole sequence, including two RUNX1 binding sites (wild type) or the deletion sequence, including one RUNX1 binding site (deletion type), was assembled into the pGL3‐ROR2 promoter. RUNX1 siRNA (Santa Cruz Biotechnology, Dallas, TX, USA) was also transferred into cell to detect the interaction between RUNX1 and this rep‐SV in MDBK cell lines. The sequence of RUNX1 siRNA was “LDDQTKPGSLSFSERLSELEQLRRTAMRVSPHHPAPTPNPRASLNHSTAFNPQPQSQMQDTRQI.”

### 5.9 Zebrafish Care and Maintenance

Adult wild‐type AB strain zebrafish were maintained at 28.5°Con a 14 h light/10 h dark cycle [77]. Five to six pairs of zebrafish were set up for nature mating every time. On average, 200–300 embryos were generated. Embryos were maintained at 28.5°C in fish water (0.2% Instant Ocean Salt in deionized water). The embryos were washed and staged according to previous research [78]. The zebrafish facility at SMOC (Shanghai Model Organisms Center, Inc.) is accredited by the Association for Assessment and Accreditation of Laboratory Animal Care (AAALAC) International.

### 5.10 Zebrafish Microinjections

*ROR2* (cattle) mRNA were microinjected into fertilized one‐cell stage embryos. The concentration of the mRNA used for injection was 150 ng/µL. Embryos were anesthetized with 0.016% MS‐222 (tricaine methanesulfonate, Sigma–Aldrich, St. Louis, MO). Zebrafish were then oriented on the lateral side (anterior, left; posterior, right; dorsal, top), and mounted with 3% methylcellulose in a depression slide for observation by fluorescence microscopy. The phenotype of whole body length was analyzed.

### 5.11 Image Acquisition

Embryos and larvae were analyzed with a Nikon SMZ 18 Fluorescence microscope and subsequently photographed with digital cameras. A subset of images was adjusted for levels, brightness, contrast, hue, and saturation with Adobe Photoshop 7.0 software (Adobe, San Jose, California) to optimally visualize the expression patterns. Quantitative image analyses processed using image‐based morphometric analysis (NIS‐Elements D4.6, Japan) and ImageJ software (U.S. National Institutes of Health, Bethesda, MD, USA; http://rsbweb.nih.gov/ij/). Inverted fluorescent images were used for processing. Positive signals were defined by particle number using ImageJ. 10 animals for each treatment were quantified, and the total signal per animal was averaged.

### 5.12 Statistical Analysis

All data are presented as mean ± SD. Statistical analysis and graphical representation of the data were performed using GraphPad Prism 6.0 (GraphPad Software, San Diego, CA). Statistical significance was performed using a Student's *t*‐test. Statistical significance is indicated by ^*^, where *p* < 0.05.

### 5.13 ANOVA Analysis

We retrieved phenotypic data on birth weight, weaning weight, and average daily gain to weaning weight (manually calculated based on the former two) from previous studies for Angus [79], Holstein [80], Hereford [81], Jersey [82], Hanwoo [83], Simmental [84], Luxi, Nanyang, and Tibetan cattle in China [85]. Since individual data were unavailable, the average values were considered the breed trait for each individual within this breed. The correlation between the genotype of the candidate variant and breed phenotypic data was statistically estimated using the univariate ANOVA test in R, with the ‘aov’ function. A *p*‐value ≤0.05 was used as the criterion for statistical significance.

### 5.14 RNA‐seq Analysis

We collected hump and testis tissues from four castrated indicine with low hump and three normal indicine with normal hump (Table S1). We used the HISAT2 v2.2.1 [86], StringTie v2.2.1 [87], and DESeq2 v1.48.1 [88] packages in R to map the paired‐end reads to the ARSUCD1.2 reference, assembly the reads, and estimate the gene expression levels, respectively. Parallel analyses of transcriptomic comparisons were conducted in the testis between taurine and indicine, which were downloaded through the accession numbers: PRJNA832033, PRJEB46995, and PRJNA417062 from NCBI.

## Author Contributions

Z.G., J.L., A.C.A., X.J., J.M., J X., and Y.L. contributed equally to this work. Z.G. and Y.L. performed the analysis. J.L., X.J., J.X., J.M., H.W., and J.C. designed and conducted the experiments. A.C.A., K.Q., Z.H., T.Y., and A.E. prepared the DNA samples. Y.L., J. L., O.H., and Y.‐P.Z. conceived the project and designed the research. Z.F.G., A.C.A., O.H., and Y.L. drafted and revised the manuscript. All authors read and helped to improve the manuscript.

## Ethics Statement

This study was approved by the Yunnan University Laboratory Animal Center Protocol no. YNU20210114.

## Conflicts of Interest

The authors declare no conflicts of interest.

## Supporting information




**Supporting File**: advs74719‐sup‐0001‐SuppMat.docx.

## Data Availability

The data that support the findings of this study are openly available in NGDC at https://ngdc.cncb.ac.cn/gsub/submit/gsa/subCRA056069/finishedOverview, reference number PRJCA052166.
