# Supplementary material for: Deciphering the Evolution Pattern of Structural Variations Overlapped With Repetitive Sequence During Cattle Evolution
Source: Adv Sci (Weinh). 2026 Mar 9;13(28):e23333. doi: 10.1002/advs.202523333 (PMC13185825; doi:10.1002/advs.202523333)
Supplement: Supplementary file 1 — Supporting File: advs74719‐sup‐0001‐SuppMat.docx. [file ADVS-13-e23333-s001.docx]

Supporting Information

Deciphering the Evolution Pattern of Structural Variations Overlapped with Repetitive Sequence during Cattle Evolution

Zhifan Guo, Jinxiu Li, Adeniyi C Adeola, Xueyan Jiang, Juntao Ma, Jian Xiao, Dexiang Hu, Kaixing Qu, Haihong Wu, Junren Chen, Zhanxing He, Tingting Yin, Ali Esmailizadeh, Jing Luo*, Olivier Hanotte*, Ya-Ping Zhang*, Yan Li*


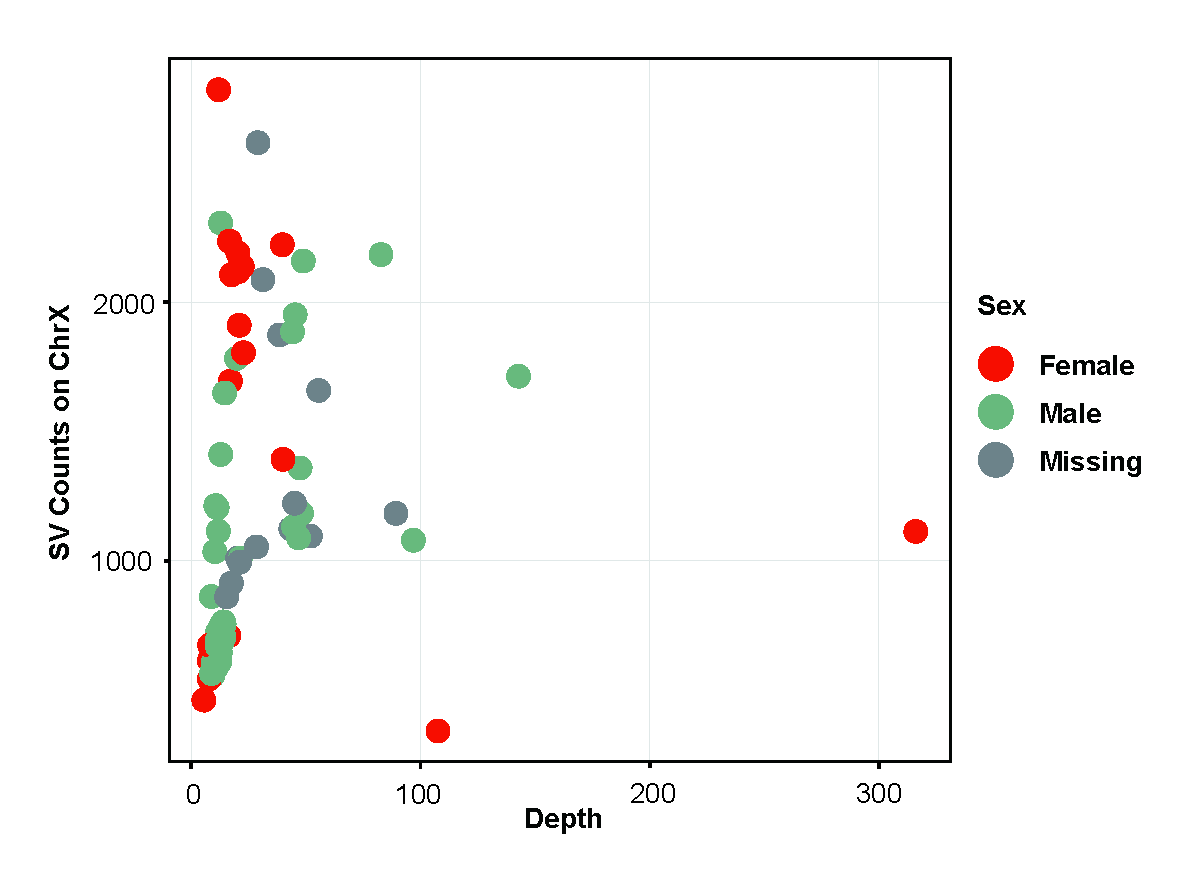


**Figure S1.**

The count of detected SV on X-chromosome for each sample with varied sequencing depth. X-axis denotes the total bases sequenced, while Y-axis denotes the counts of detected SVs on X-chromosome.

**
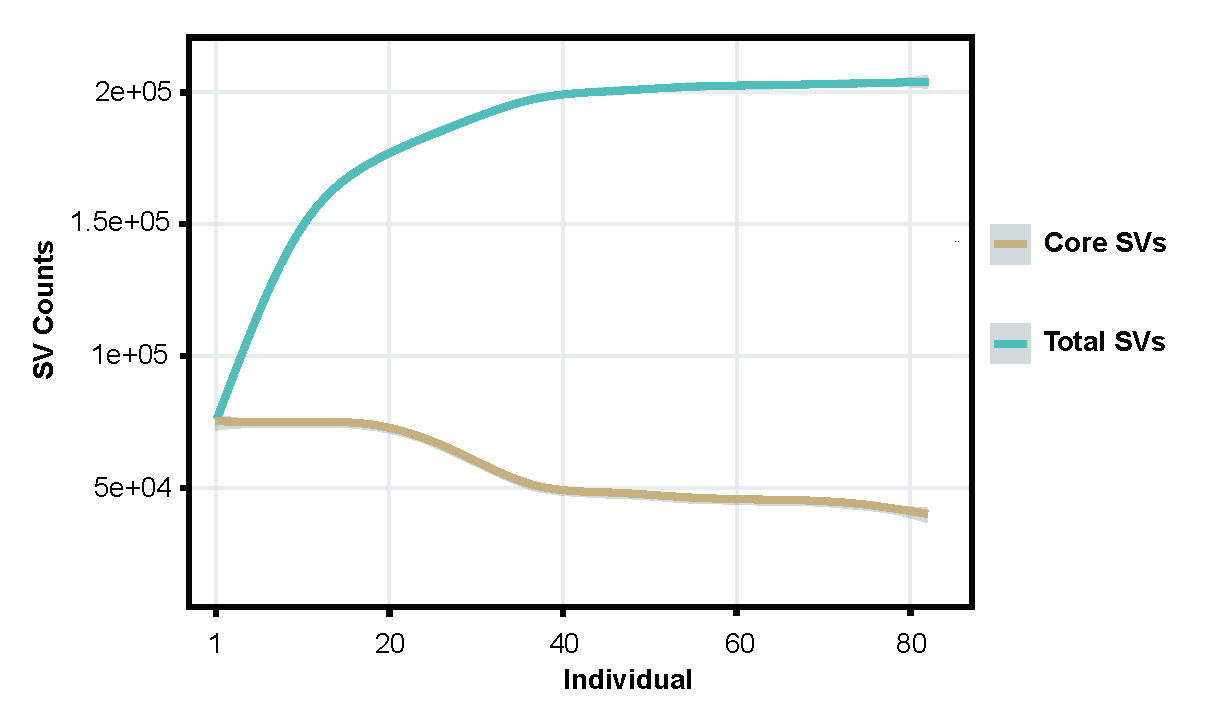
**

**Figure S2.**

Model describing the sizes of core and panSV. Simulations of the increase in pan-SV number and decrease in core-SV detected with the increase of bovine number.

**
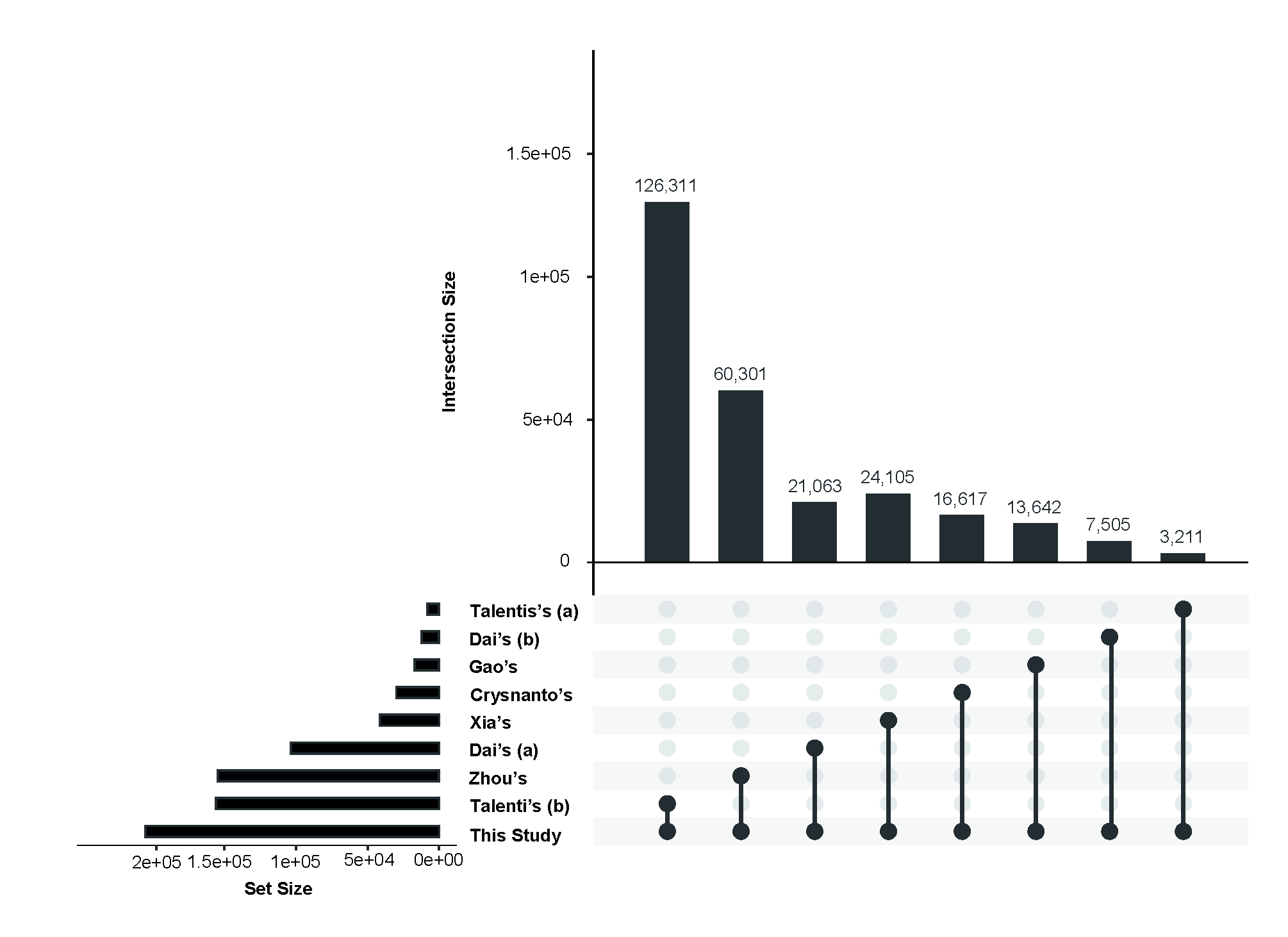
**

**Figure S3.**

Intersection of non-reference alleles detected across eight published studies. Talenti (a) used high-coverage (100×) Bionano optical mapping data from 18 cattle representing nine globally distributed breeds across three continents to characterize structural variation at the species level, while Talenti (b) generated de novo assemblies for the N’Dama and Ankole breeds and constructed a cattle graph pangenome by integrating these assemblies with three public reference genomes (Hereford, Angus, and Brahman), together with resequencing data from 294 individuals representing global cattle diversity. Dai (a) generated partially phased genomes assemblies from 10 Chinese indicine breeds sampled in southern China to define the landscape of non-Hereford sequences, while Dai (b) analyzed short-read whole-genome sequencing data from 2,409 individuals spanning 82 breeds to characterize the genetic architecture of global cattle populations based on a pangenome platform.

**
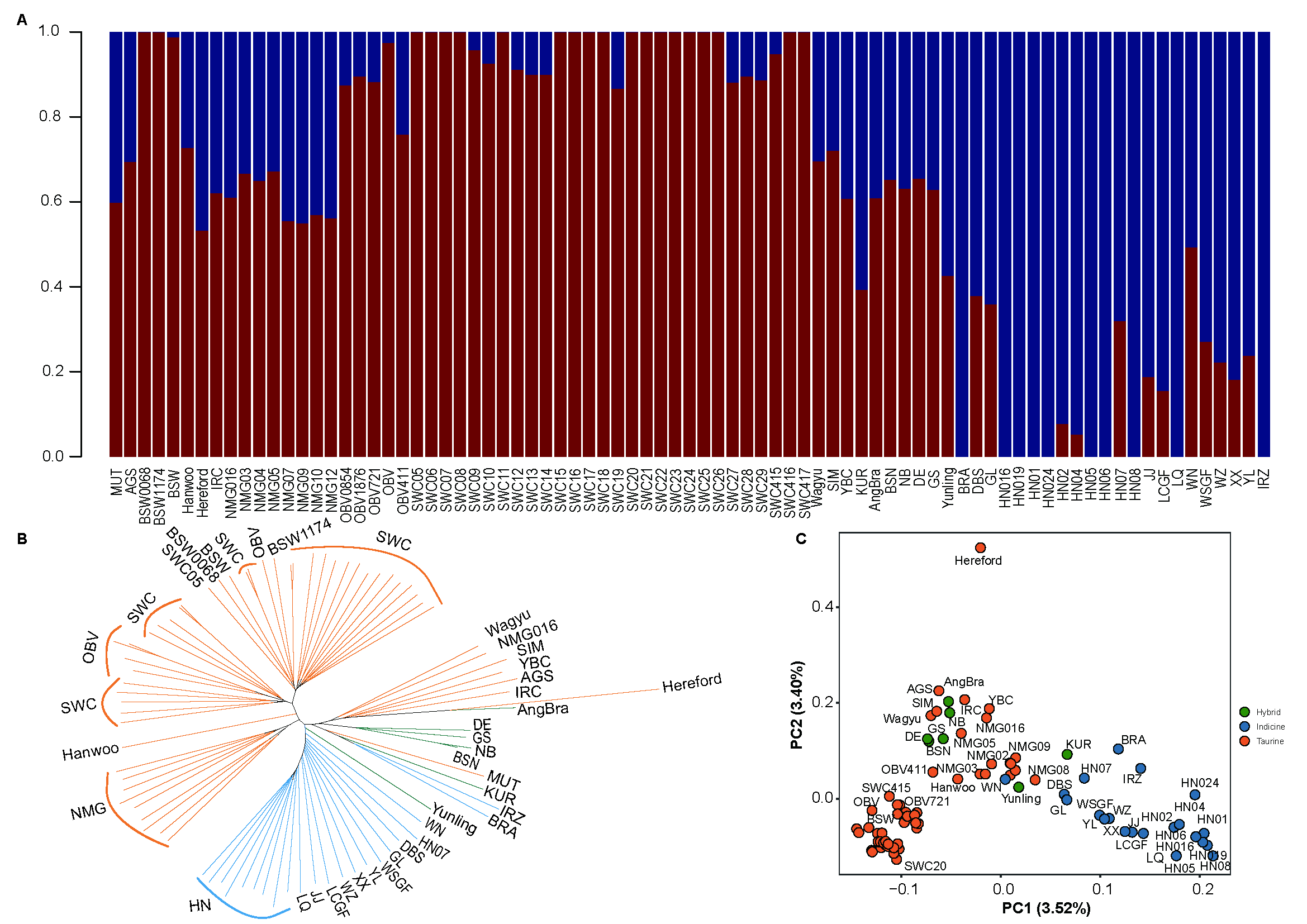
**

**Figure S4.**

Population structure and relationship of eight sampled bovine breeds. (**A**), Results of admixture analysis at K=2. (**B**), Neighbor joining tree constructed using SV data, with distinct branch colors representing different species. (**C**), Principal component analysis (PCA) results of 83 LRS samples. The sampled breeds are indicated in arrowhead.

**
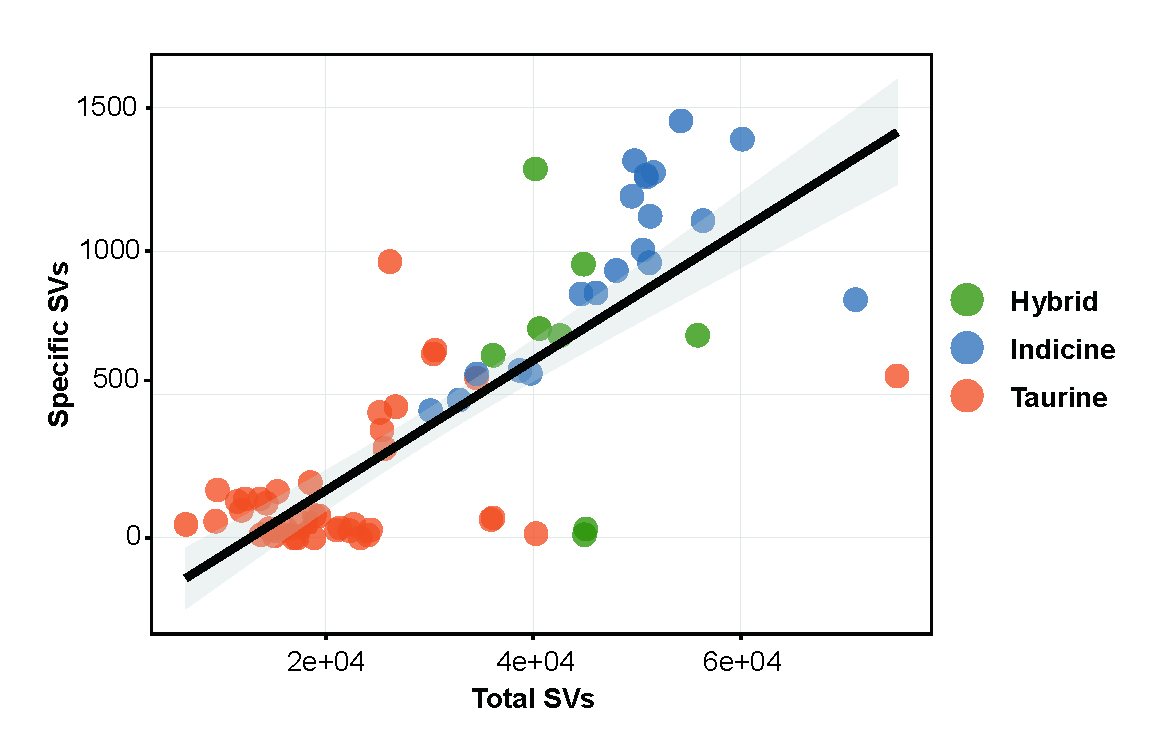
**

**Figure S5.**

Breed-Specific and total SVs across domestic cattle.

**
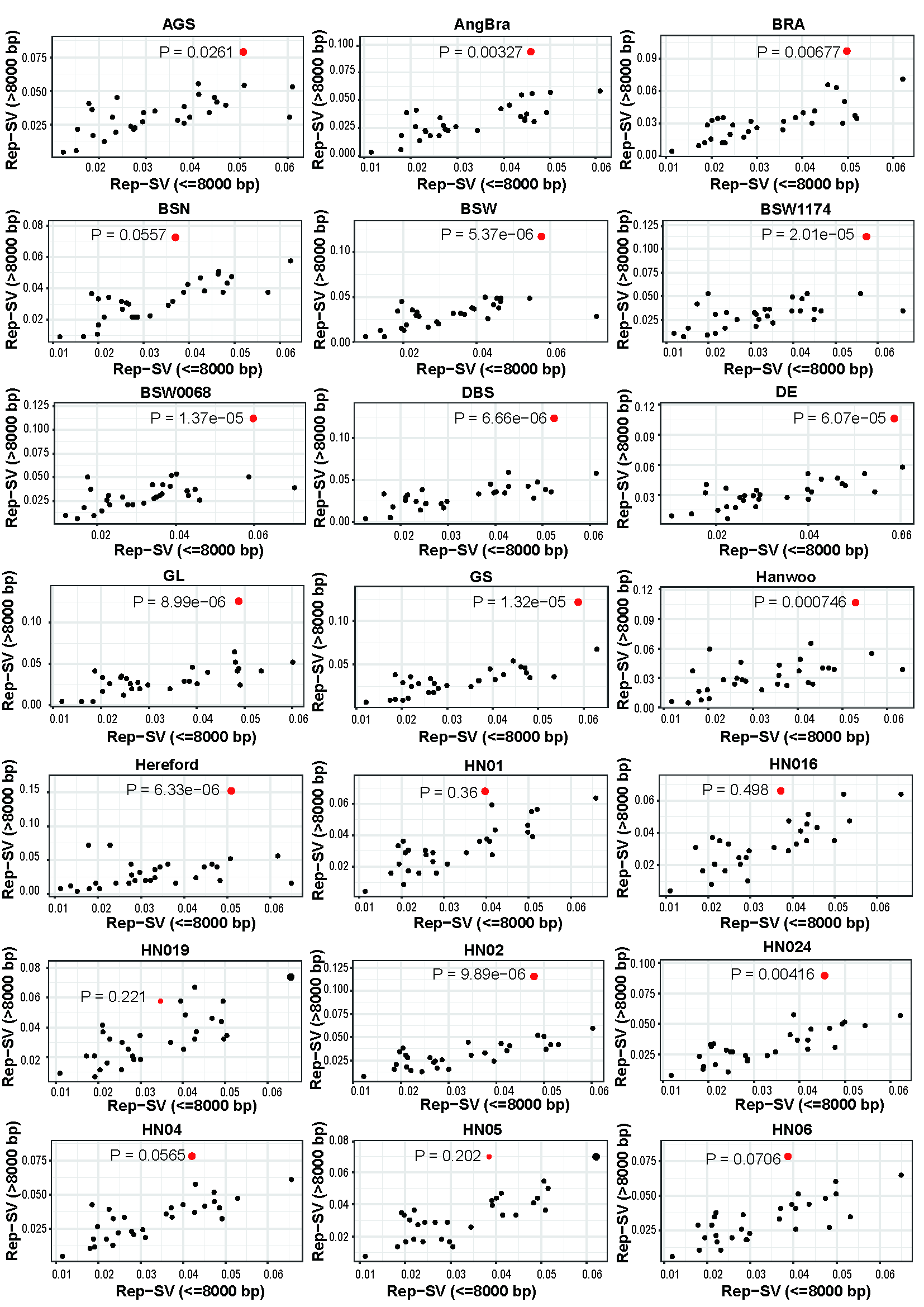
**

**
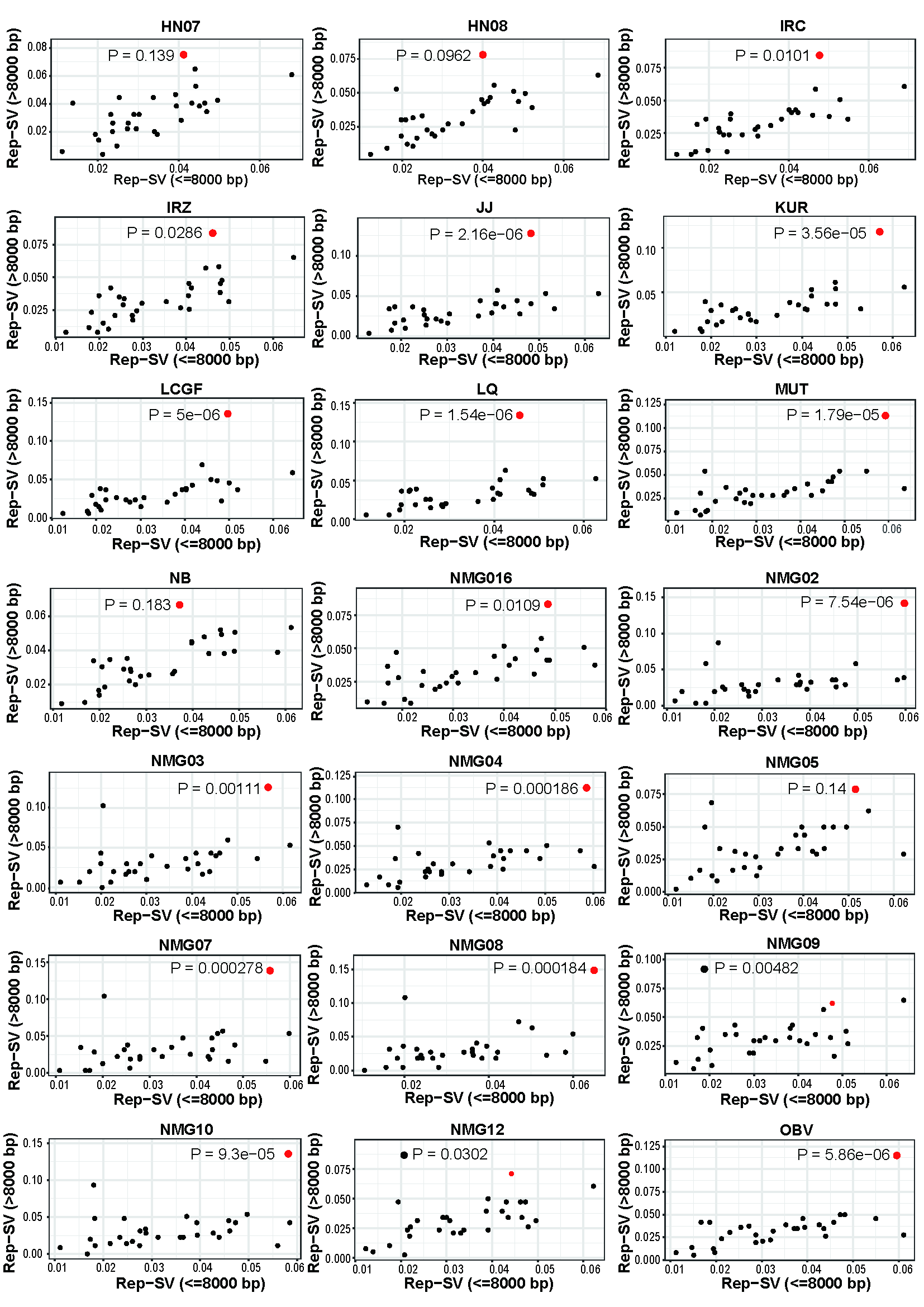
**

**
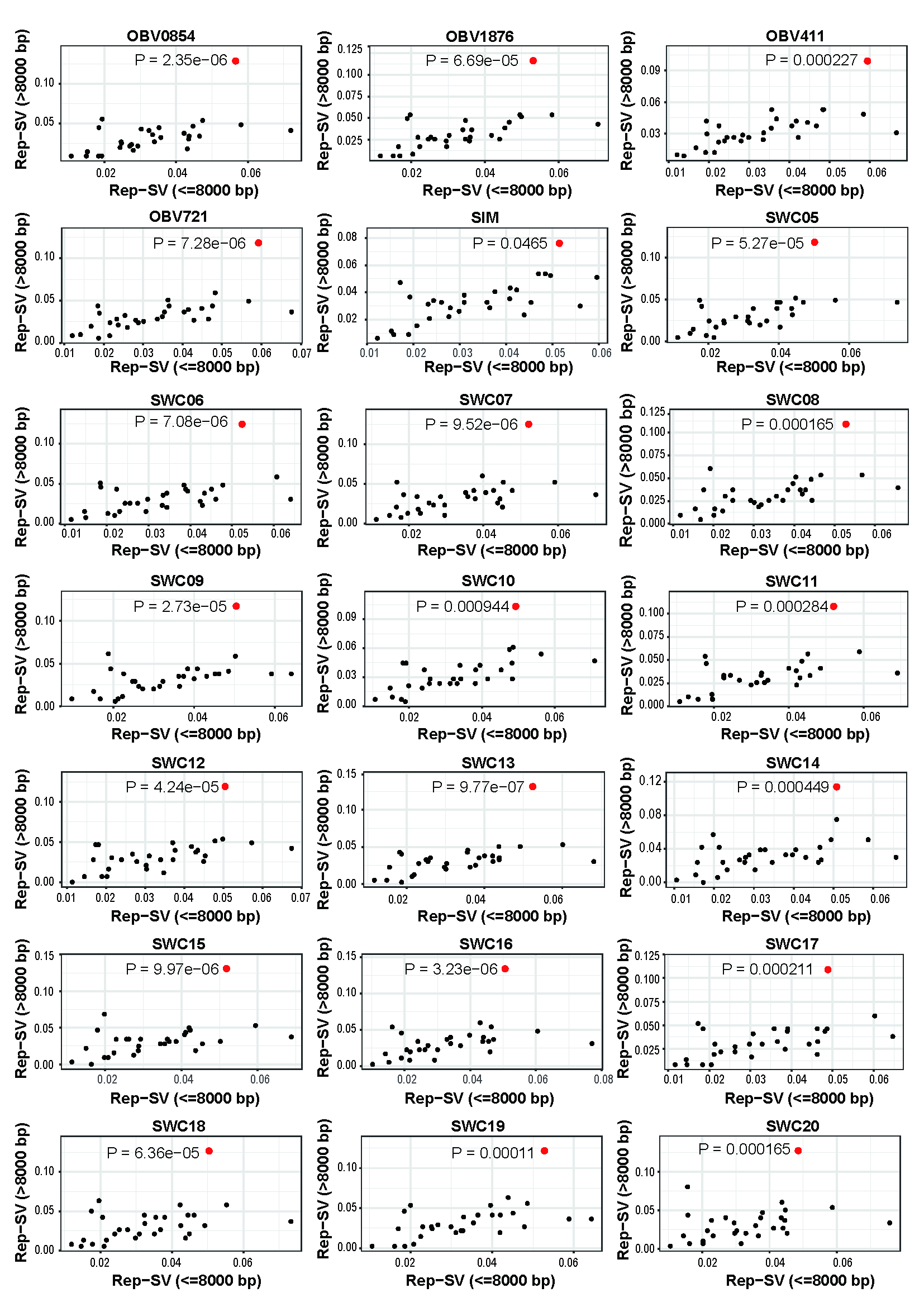
**

**
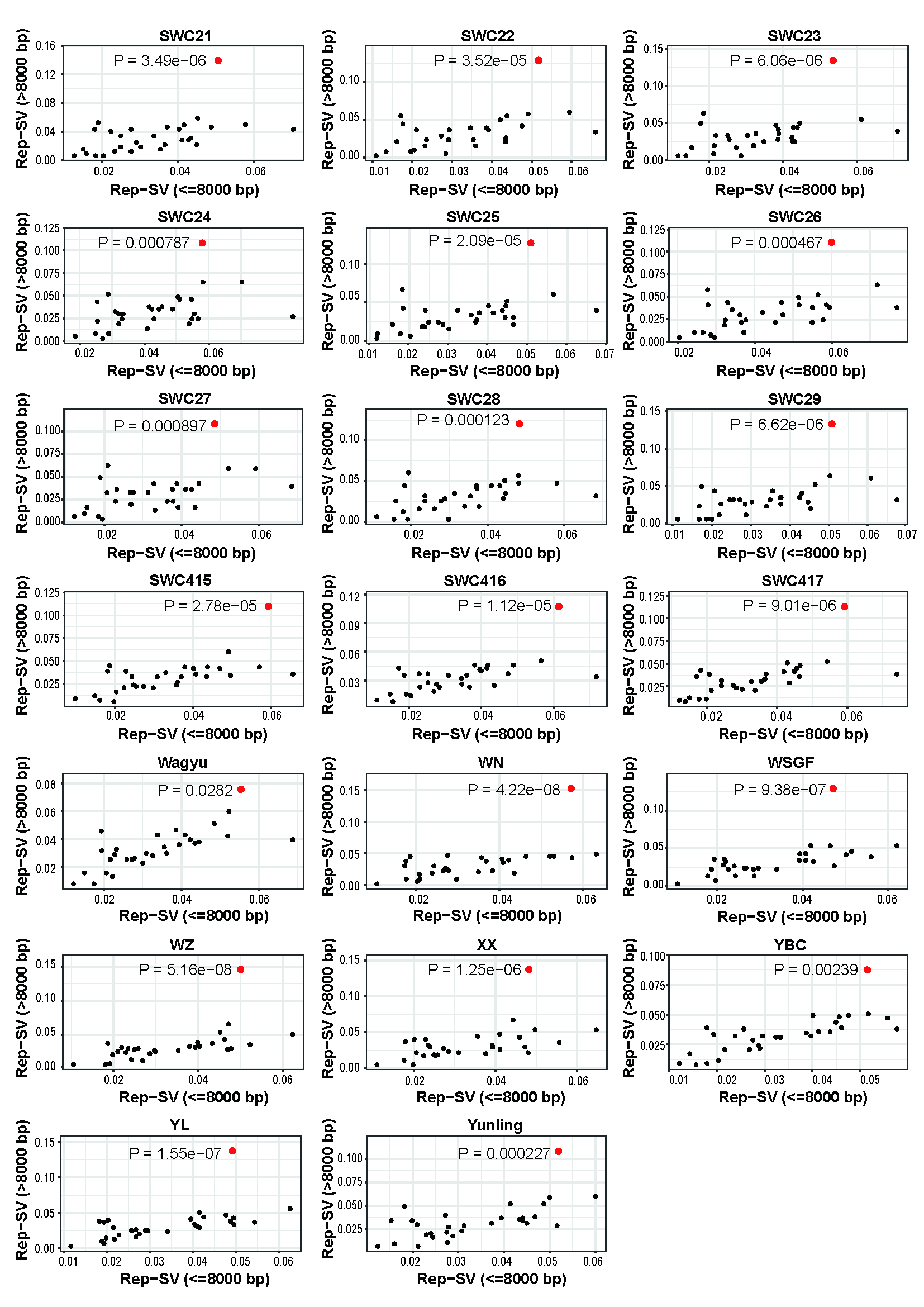
**

**Figure S6.**

The proportions of rep-SVs within each chromosome for each individual. X axis denotes rep-SVs over 8000 bp length. Y axis denotes rep-SVs under 8000 bp length. The black dots denote autosomes, while the red dots denote the X chromosome. The P values were calculated using Grubbs test.

**
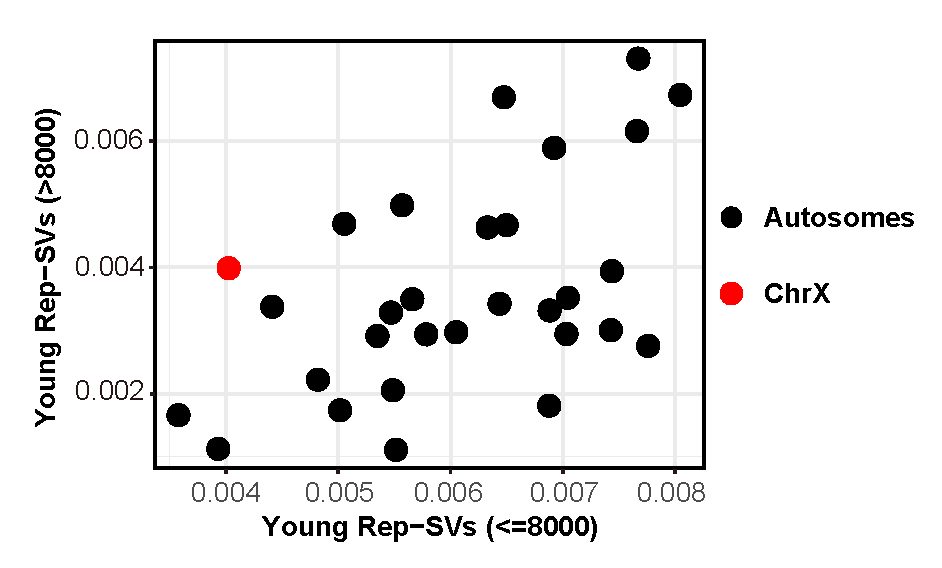
**

**Figure S7.**

The proportions of young rep-SVs within each chromosome. X axis denotes rep-SVs over 8000 bp length. Y axis denotes rep-SVs under 8000 bp length. The black dots denote autosomes, while the red dots denote the X chromosome.


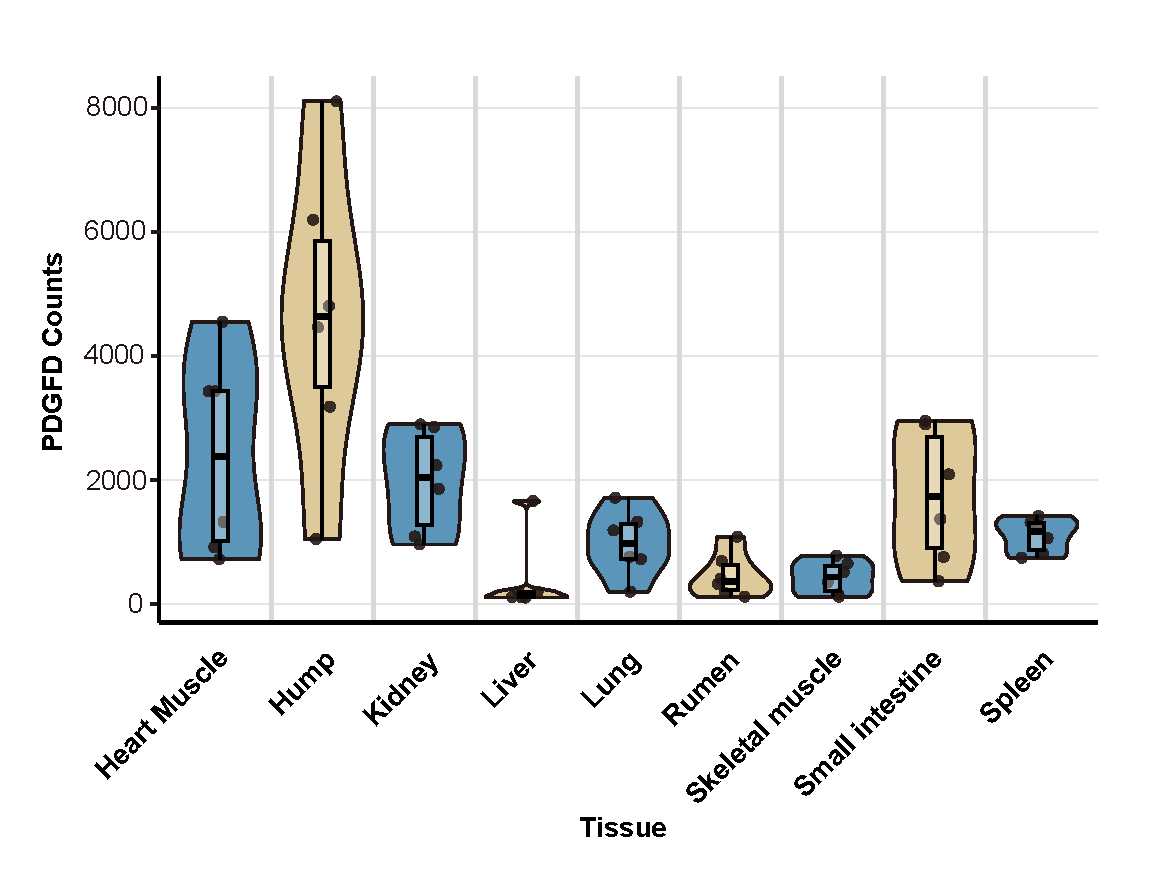


**Figure S8.**

PDGFD expression in nine tissues of Bactrian camel


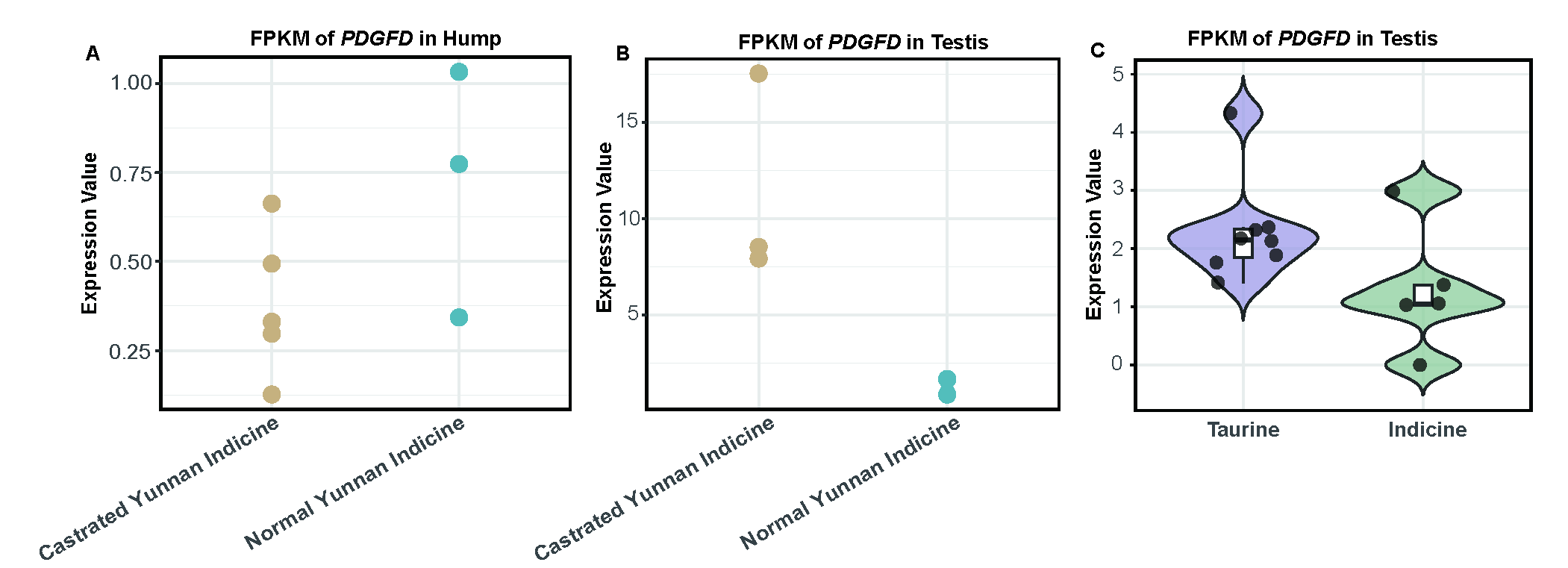


**Figure S9.**

*PDGFD* expression in the hump and testis. of castrated indicine and normal indicine. The *PDGFD* FPKM of hump **(A)** and testis **(B)** in castrated indicine with low hump and normal indicine with normal hump. **C**) The *PDGFD* FPKM of testis in FPKM of PDGFD in testis across Taurine and Indicine. Taurine includes 3 Simmental adults (PRJNA832033) and 5 taurus bulls (presumed to be Switzerland Braunvieh according to additional sample metadata from the same project: PRJEB46995); Indicine includes 5 zebu adults with unspecified breed informtion; PRJNA417062).


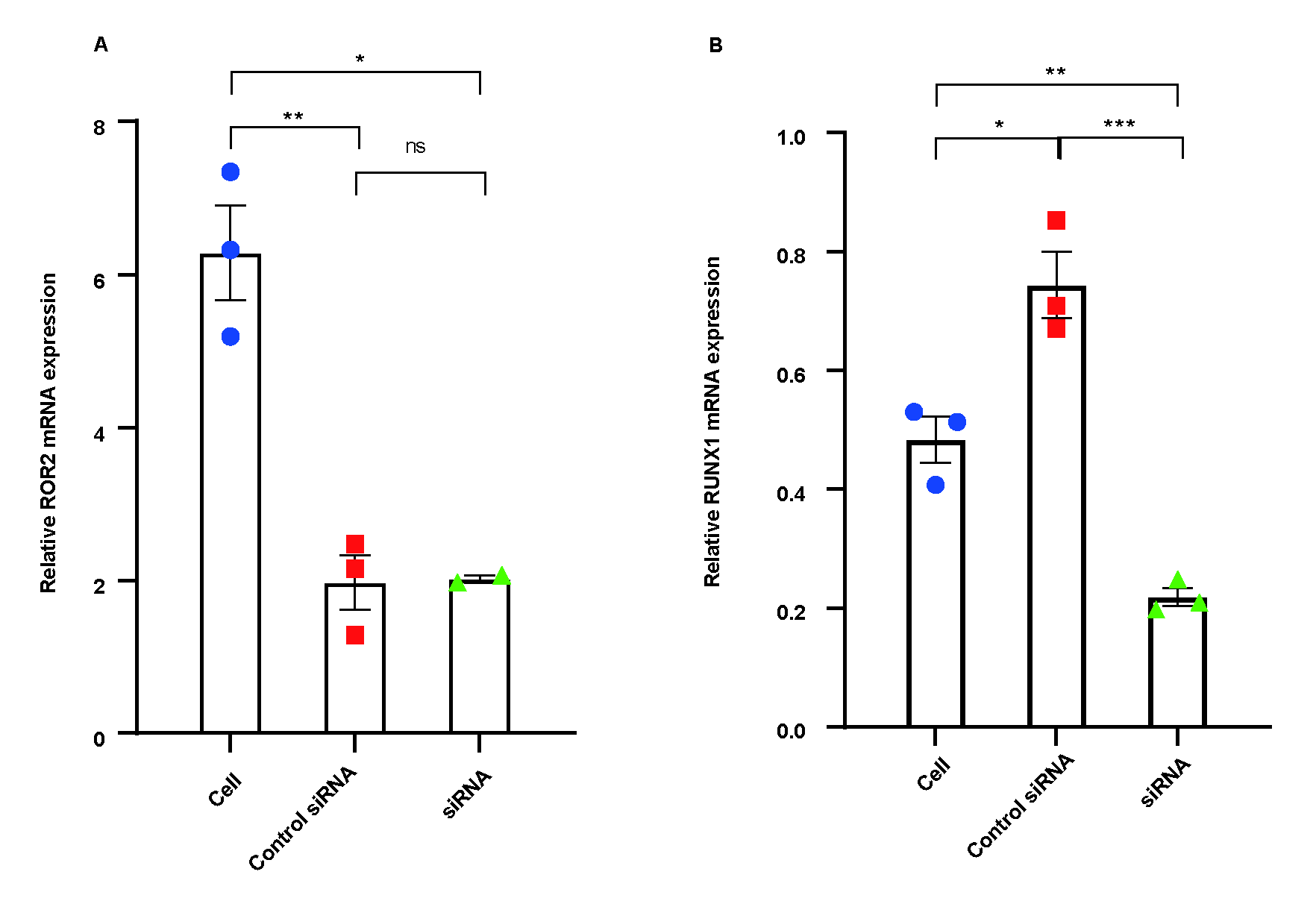


**Figure S10.**

The efficacy of siRNA interference on ROR2 (A) and RUNX1 (B).


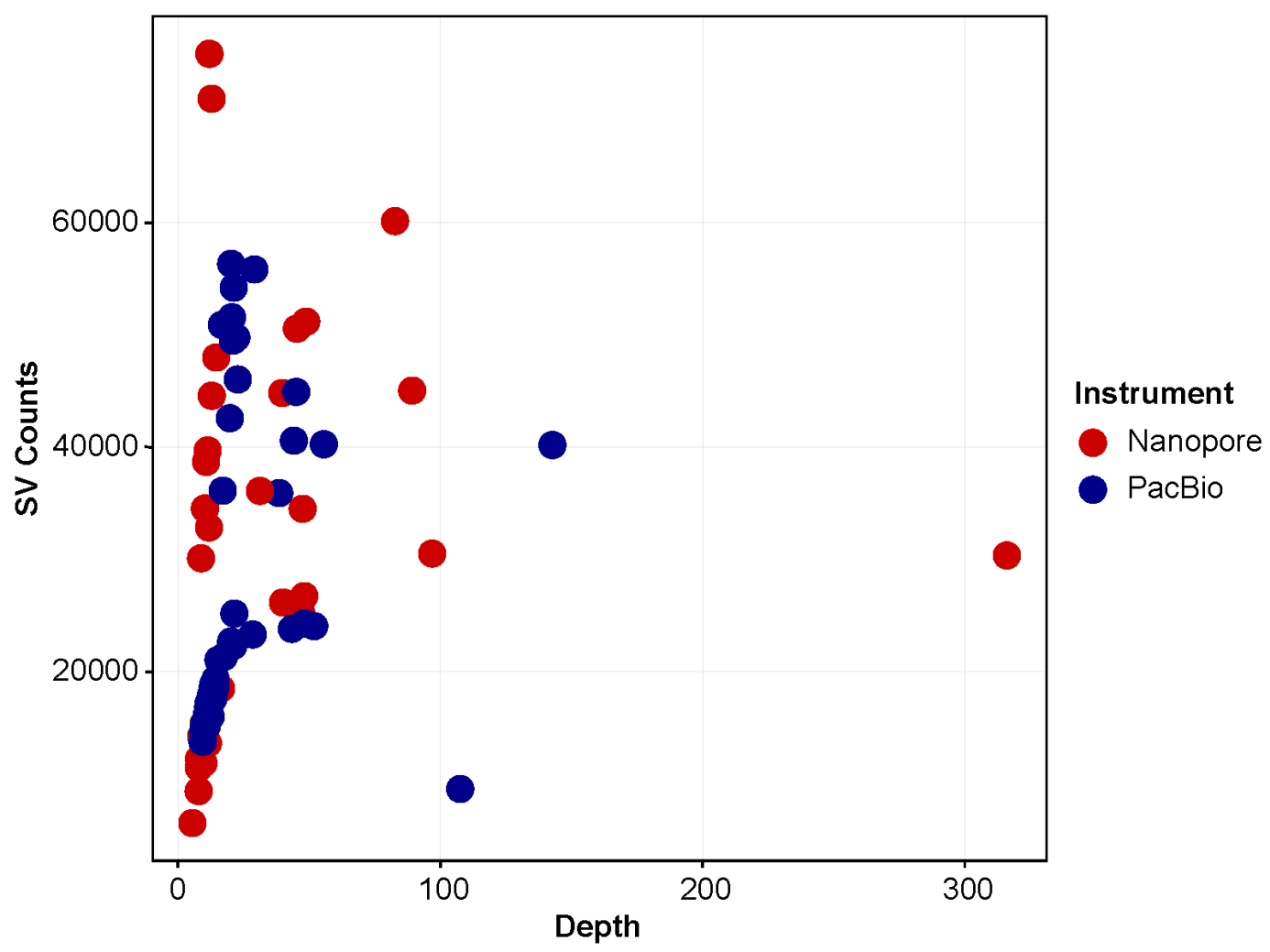


**Figure S11.**

SV detection rates according to two long-read sequencing platforms.

**Table S1.**

**Summary of samples collected in this study**

| **Sample ID** | **Breed** | **Species** | **Sex** | **Age** | **Tissue** | **Geographic location** | **Bioproject** | **Sequencing data type** |
| --- | --- | --- | --- | --- | --- | --- | --- | --- |
| AGS | Angus | Bos taurus | Male | Adult | blood | Europe | PRJCA052166 | Oxford Nanopore |
| SIM | Simmental | Bos taurus | Male | Adult | blood | Europe | PRJCA052166 | Oxford Nanopore |
| YBC | Yanbian cattle | Bos taurus | Male | Adult | blood | China | PRJCA052166 | Oxford Nanopore |
| IRC | Iran cattle | Bos taurus | Male | Adult | blood | Iran | PRJCA052166 | Oxford Nanopore |
| MUT | Muturu | Bos taurus | Female | Adult | blood | Africa | PRJCA052166 | Oxford Nanopore |
| KUR | Kuri | Bos taurus x Bos indicus | Female | Adult | blood | Africa | PRJCA052166 | Oxford Nanopore |
| IRZ | Iran cattle | Bos indicus | Male | Adult | blood | Iran | PRJCA052166 | Oxford Nanopore |
| BRA | Brahman | Bos indicus | Male | Adult | blood | Australia | PRJCA052166 | Oxford Nanopore |
| 1H | Castrated Yunnan Indicine | Bos indicus | Male | Adult | hump | China | PRJCA052166 | RNA-seq |
| 1T | Castrated Yunnan Indicine | Bos indicus | Male | Adult | testis | China | PRJCA052166 | RNA-seq |
| 2H | Castrated Yunnan Indicine | Bos indicus | Male | Adult | hump | China | PRJCA052166 | RNA-seq |
| 3H | Normal Yunnan Indicine | Bos indicus | Male | Adult | hump | China | PRJCA052166 | RNA-seq |
| 3T | Normal Yunnan Indicine | Bos indicus | Male | Adult | testis | China | PRJCA052166 | RNA-seq |
| 4H | Castrated Yunnan Indicine | Bos indicus | Male | Adult | hump | China | PRJCA052166 | RNA-seq |
| 4T | Castrated Yunnan Indicine | Bos indicus | Male | Adult | testis | China | PRJCA052166 | RNA-seq |
| 5H | Castrated Yunnan Indicine | Bos indicus | Male | Adult | hump | China | PRJCA052166 | RNA-seq |
| 5T | Castrated Yunnan Indicine | Bos indicus | Male | Adult | testis | China | PRJCA052166 | RNA-seq |
| 6H | Normal Yunnan Indicine | Bos indicus | Male | Adult | hump | China | PRJCA052166 | RNA-seq |
| 6T | Normal Yunnan Indicine | Bos indicus | Male | Adult | testis | China | PRJCA052166 | RNA-seq |
| 7H | Normal Yunnan Indicine | Bos indicus | Male | Adult | hump | China | PRJCA052166 | RNA-seq |
| 7T | Normal Yunnan Indicine | Bos indicus | Male | Adult | testis | China | PRJCA052166 | RNA-seq |
| 8H | Castrated Yunnan Indicine | Bos indicus | Female | Adult | hump | China | PRJCA052166 | RNA-seq |

**Table S2.**

**Base quality of samples sequenced in this study**

| **Sample ID** | **Base(bp)** | **Mean Depth** | **Lenth N50 (bp)** | **Mean Length (bp)** | **Max Length (bp)** |
| --- | --- | --- | --- | --- | --- |
| AGS | 150,064,300,420 | 46.83 | 33,002 | 26,615 | 247,509 |
| SIM | 149,959,463,430 | 44.61 | 21,669 | 18,798 | 200,526 |
| YBC | 154,648,302,208 | 48.13 | 28,618 | 22,740 | 1,445,358 |
| IRC | 152,304,363,177 | 47.53 | 29,991 | 24,037 | 269,289 |
| MUT | 126,418,190,906 | 40.04 | 20,975 | 18,109 | 769,556 |
| KUR | 123,942,305,843 | 39.75 | 26,677 | 22,431 | 890,807 |
| IRZ | 143,102,566,342 | 45.35 | 16,910 | 14,062 | 186,615 |
| BRA | 150,924,784,344 | 48.88 | 25,016 | 20,698 | 245,788 |

**Table S3.**

**Summary of long-reads sequencing data from NCBI**

| **Sample ID** | **Breed** | **Species** | **Country** | **Sex** | **Tissue** | **Sequencing Platform** | **Base** | **Mean Depth** | **Mean Length** | **Length N50** | **SRR Accession** | **Bioproject** |
| --- | --- | --- | --- | --- | --- | --- | --- | --- | --- | --- | --- | --- |
| HN024 | Hainan cattle | Bos indicus | China | Male | Blood | Nanopore (PromethION) | 285,056,572,955 | 82.77 | 19,385 | 24,376 | SRR18614805 | PRJNA823479 |
| HN019 | Hainan cattle | Bos indicus | China | Male | Blood | Nanopore (PromethION) | 27,300,006,387 | 8.79 | 15,377 | 24,706 | SRR18614806 | PRJNA823479 |
| HN016 | Hainan cattle | Bos indicus | China | Male | Blood | Nanopore (PromethION) | 31,950,915,840 | 10.28 | 17,509 | 24,610 | SRR18614807 | PRJNA823479 |
| HN08 | Hainan cattle | Bos indicus | China | Male | Blood | Nanopore (PromethION) | 35,091,129,727 | 11.33 | 25,066 | 27,814 | SRR18614808 | PRJNA823479 |
| HN07 | Hainan cattle | Bos indicus | China | Male | Blood | Nanopore (PromethION) | 36,606,858,838 | 11.84 | 9,667 | 17,470 | SRR18614809 | PRJNA823479 |
| HN06 | Hainan cattle | Bos indicus | China | Male | Blood | Nanopore (PromethION) | 39,729,021,339 | 12.88 | 15,114 | 25,831 | SRR18614810 | PRJNA823479 |
| HN05 | Hainan cattle | Bos indicus | China | Male | Blood | Nanopore (PromethION) | 33,486,984,718 | 10.73 | 28,380 | 33,273 | SRR18614811 | PRJNA823479 |
| HN04 | Hainan cattle | Bos indicus | China | Male | Blood | Nanopore (PromethION) | 45,227,318,521 | 14.61 | 26,361 | 31,469 | SRR18614812 | PRJNA823479 |
| NMG016 | Mongolian cattle | Bos taurus | China | Male | Heart | Nanopore (PromethION) | 335,338,674,377 | 96.92 | 21,025 | 26,893 | SRR18614813 | PRJNA823479 |
| NMG12 | Mongolian cattle | Bos taurus | China | Male | Heart | Nanopore (PromethION) | 29,004,620,093 | 8.82 | 24,406 | 29,194 | SRR18614814 | PRJNA823479 |
| NMG10 | Mongolian cattle | Bos taurus | China | Female | Heart | Nanopore (PromethION) | 25,910,461,972 | 8.05 | 22,911 | 27,399 | SRR18614815 | PRJNA823479 |
| NMG09 | Mongolian cattle | Bos taurus | China | Male | Heart | Nanopore (PromethION) | 30,597,448,575 | 9.72 | 24,126 | 28,388 | SRR18614816 | PRJNA823479 |
| NMG08 | Mongolian cattle | Bos taurus | China | Female | Heart | Nanopore (PromethION) | 17,319,103,757 | 5.49 | 22,716 | 26,771 | SRR18614817 | PRJNA823479 |
| NMG07 | Mongolian cattle | Bos taurus | China | Female | Heart | Nanopore (PromethION) | 24,883,406,767 | 7.94 | 23,696 | 27,905 | SRR18614818 | PRJNA823479 |
| NMG05 | Mongolian cattle | Bos taurus | China | Female | Heart | Nanopore (PromethION) | 50,437,651,711 | 16.42 | 13,183 | 21,082 | SRR18614819 | PRJNA823479 |
| NMG04 | Mongolian cattle | Bos taurus | China | Male | Heart | Nanopore (PromethION) | 34,582,304,716 | 11.42 | 12,041 | 22,873 | SRR18614820 | PRJNA823479 |
| NMG03 | Mongolian cattle | Bos taurus | China | Male | Heart | Nanopore (PromethION) | 30,142,577,161 | 9.71 | 11,804 | 19,144 | SRR18614821 | PRJNA823479 |
| NMG02 | Mongolian cattle | Bos taurus | China | Female | Heart | Nanopore (PromethION) | 24,217,691,320 | 7.88 | 23,982 | 28,340 | SRR18614822 | PRJNA823479 |
| HN02 | Hainan cattle | Bos indicus | China | Female | Blood | Nanopore (PromethION) | 36,992,775,423 | 11.98 | 28,271 | 32,480 | SRR18614823 | PRJNA823479 |
| HN01 | Hainan cattle | Bos indicus | China | Male | Blood | Nanopore (PromethION) | 39,704,982,927 | 12.84 | 15,402 | 25,147 | SRR18614824 | PRJNA823479 |
| YL | Yiling cattle | Bos indicus | China | Female | Blood | Pacbio (Sequel II) | 58,783,834,295 | 20.00 | 13,948 | 13,946 | SRR17257200 | PRJNA786777 |
| XX | Xiangxi cattle | Bos indicus | China | Female | Blood | Pacbio (Sequel II) | 51,157,258,507 | 17.58 | 12,812 | 12,858 | SRR17257201 | PRJNA786777 |
| WSGF | Wenshangaofeng cattle | Bos indicus | China | Female | Blood | Pacbio (Sequel II) | 60,557,161,585 | 20.94 | 13,001 | 13,058 | SRR17257202 | PRJNA786777 |
| WZ | Weizhou cattle | Bos indicus | China | Female | Blood | Pacbio (Sequel II) | 49,953,201,663 | 16.73 | 15,122 | 15,249 | SRR17257203 | PRJNA786777 |
| WN | Weining cattle | Bos indicus | China | Female | Blood | Pacbio (Sequel II) | 51,157,231,306 | 17.08 | 11,229 | 11,590 | SRR17257204 | PRJNA786777 |
| LCGF | Lincanggaofeng cattle | Bos indicus | China | Female | Blood | Pacbio (Sequel II) | 59,241,544,231 | 20.59 | 12,501 | 12,573 | SRR17257205 | PRJNA786777 |
| LQ | Leiqiong cattle | Bos indicus | China | Female | Blood | Pacbio (Sequel II) | 58,716,144,599 | 20.29 | 13,332 | 13,416 | SRR17257206 | PRJNA786777 |
| GL | Guanling cattle | Bos indicus | China | Female | Blood | Pacbio (Sequel II) | 66,631,915,231 | 22.81 | 13,459 | 13,434 | SRR17257208 | PRJNA786777 |
| DBS | Dabieshan cattle | Bos indicus | China | Female | Blood | Pacbio (Sequel II) | 64,188,730,603 | 22.27 | 16,009 | 15,682 | SRR17257209 | PRJNA786777 |
| JJ | Jinjiang cattle | Bos indicus | China | Female | Blood | Pacbio (Sequel II) | 63,065,608,716 | 21.24 | 14,987 | 15,034 | SRR17257207 | PRJNA786777 |
| BSW0068 | Brown Swiss | Bos taurus | Switzerland | Missing | Blood | Pacbio (Sequel) | 63,803,456,999 | 21.08 | 19,653 | 19,806 | ERR10166765 | PRJEB42335 |
| BSW1174 | Brown Swiss | Bos taurus | Switzerland | Missing | Blood | Pacbio (Sequel) | 55,170,824,774 | 17.53 | 19,791 | 20,063 | ERR10166766 | PRJEB42335 |
| OBV0854 | Original Braunvieh | Bos taurus | Switzerland | Missing | Blood | Pacbio (Sequel) | 62,779,438,653 | 20.25 | 16,430 | 16,629 | ERR10166767 | PRJEB42335 |
| OBV1876 | Original Braunvieh | Bos taurus | Switzerland | Missing | Blood | Pacbio (Sequel) | 47,154,991,463 | 15.43 | 15,872 | 16,121 | ERR10166768 | PRJEB42335 |
| BSW | Brown Swiss | Bos taurus | Switzerland | Missing | Blood | Pacbio (Sequel IIe) | 111,768,503,017 | 38.60 | 20,734 | 21,524 | ERR12522410 | PRJEB42335 |
| OBV411 | Original Braunvieh | Bos taurus | Switzerland | Missing | Testis | Pacbio (Revio) | 156,303,622,618 | 55.62 | 13,881 | 14,970 | ERR12522411 | PRJEB42335 |
| GS | Gir x Simmental | Bos indicus x Bos taurus | Switzerland | Missing | Blood | Pacbio (Sequel IIe) | 89,442,884,125 | 29.11 | 16,416 | 18,819 | ERR12522412 | PRJEB42335 |
| DE | Dwarf zebu x Evolener | Bos indicus x Bos taurus | Switzerland | Missing | Blood | Pacbio (Sequel IIe) | 139,143,231,928 | 44.19 | 20,423 | 21,362 | ERR12522413 | PRJEB42335 |
| SWC415 | Switzerland cattle | Bos taurus | Switzerland | Missing | Testis | Pacbio (Revio) | 135,439,622,276 | 48.10 | 12,978 | 13,700 | ERR12522415 | PRJEB42335 |
| SWC416 | Switzerland cattle | Bos taurus | Switzerland | Missing | Testis | Pacbio (Revio) | 122,537,357,527 | 43.43 | 12,996 | 13,895 | ERR12522416 | PRJEB42335 |
| SWC417 | Switzerland cattle | Bos taurus | Switzerland | Missing | Testis | Pacbio (Revio) | 144,613,840,838 | 51.94 | 13,723 | 14,722 | ERR12522417 | PRJEB42335 |
| OBV721 | Original Braunvieh | Bos taurus | Switzerland | Missing | Missing | Pacbio (Sequel II) | 86,877,101,902 | 28.47 | 19,339 | 19,621 | ERR5037721 | PRJEB42335 |
| BSN | Brown Swiss x Nelore | Bos indicus x Bos taurus | Switzerland | Missing | Missing | Pacbio (Sequel II) | 140,232,424,322 | 45.08 | 20,612 | 20,792 | ERR5043144 | PRJEB42335 |
| NB | Nelore x Brown Swiss | Bos indicus x Bos taurus | Switzerland | Missing | Missing | Nanopore (PromethION) | 277,704,422,221 | 89.29 | 30,629 | 44,830 | ERR7091271 | PRJEB42335 |
| OBV | Original Braunvieh | Bos taurus | Switzerland | Missing | Missing | Nanopore (PromethION) | 97,741,535,249 | 31.26 | 37,339 | 63,993 | ERR7091272 | PRJEB42335 |
| SWC05 | Switzerland cattle | Bos taurus | Switzerland | Male | Epididymis | Pacbio (Sequel IIe) | 37,348,451,248 | 13.74 | 14,436 | 15,233 | ERR11573705 | PRJEB46995 |
| SWC06 | Switzerland cattle | Bos taurus | Switzerland | Male | Testis | Pacbio (Sequel IIe) | 35,956,061,891 | 12.57 | 15,906 | 16,866 | ERR11573706 | PRJEB46995 |
| SWC07 | Switzerland cattle | Bos taurus | Switzerland | Male | Epididymis | Pacbio (Sequel IIe) | 38,302,338,927 | 13.92 | 14,582 | 15,210 | ERR11573707 | PRJEB46995 |
| SWC08 | Switzerland cattle | Bos taurus | Switzerland | Male | Testis | Pacbio (Sequel IIe) | 37,971,609,131 | 13.32 | 17,139 | 17,934 | ERR11573708 | PRJEB46995 |
| SWC09 | Switzerland cattle | Bos taurus | Switzerland | Male | Testis | Pacbio (Sequel IIe) | 33,843,484,153 | 11.69 | 13,058 | 13,722 | ERR11573709 | PRJEB46995 |
| SWC10 | Switzerland cattle | Bos taurus | Switzerland | Male | Testis | Pacbio (Sequel IIe) | 38,680,465,380 | 14.25 | 15,484 | 16,222 | ERR11573710 | PRJEB46995 |
| SWC11 | Switzerland cattle | Bos taurus | Switzerland | Male | Testis | Pacbio (Sequel IIe) | 35,538,386,655 | 12.92 | 15,898 | 17,073 | ERR11573711 | PRJEB46995 |
| SWC12 | Switzerland cattle | Bos taurus | Switzerland | Male | Testis | Pacbio (Sequel IIe) | 40,453,669,787 | 14.35 | 15,185 | 15,746 | ERR11573712 | PRJEB46995 |
| SWC13 | Switzerland cattle | Bos taurus | Switzerland | Male | Testis | Pacbio (Sequel IIe) | 38,245,426,793 | 13.18 | 15,400 | 16,016 | ERR11573713 | PRJEB46995 |
| SWC14 | Switzerland cattle | Bos taurus | Switzerland | Male | Testis | Pacbio (Sequel IIe) | 27,690,014,191 | 9.59 | 14,917 | 15,595 | ERR11573714 | PRJEB46995 |
| SWC15 | Switzerland cattle | Bos taurus | Switzerland | Male | Testis | Pacbio (Sequel IIe) | 33,693,420,893 | 11.67 | 13,414 | 14,232 | ERR11573715 | PRJEB46995 |
| SWC16 | Switzerland cattle | Bos taurus | Switzerland | Male | Testis | Pacbio (Sequel IIe) | 32,634,241,953 | 11.48 | 14,787 | 16,159 | ERR11573716 | PRJEB46995 |
| SWC17 | Switzerland cattle | Bos taurus | Switzerland | Male | Testis | Pacbio (Sequel IIe) | 34,614,097,855 | 12.71 | 14,024 | 14,528 | ERR11573717 | PRJEB46995 |
| SWC18 | Switzerland cattle | Bos taurus | Switzerland | Male | Testis | Pacbio (Sequel IIe) | 38,364,631,323 | 13.49 | 13,666 | 14,571 | ERR11573718 | PRJEB46995 |
| SWC19 | Switzerland cattle | Bos taurus | Switzerland | Male | Epididymis | Pacbio (Sequel IIe) | 39,341,167,707 | 14.15 | 15,305 | 16,240 | ERR11573719 | PRJEB46995 |
| SWC20 | Switzerland cattle | Bos taurus | Switzerland | Male | Testis | Pacbio (Sequel IIe) | 26,995,560,207 | 9.38 | 13,362 | 14,893 | ERR11573720 | PRJEB46995 |
| SWC21 | Switzerland cattle | Bos taurus | Switzerland | Male | Epididymis | Pacbio (Sequel IIe) | 31,046,619,800 | 10.02 | 12,671 | 14,268 | ERR11573721 | PRJEB46995 |
| SWC22 | Switzerland cattle | Bos taurus | Switzerland | Male | Epididymis | Pacbio (Sequel IIe) | 33,748,147,538 | 11.51 | 16,028 | 16,728 | ERR11573722 | PRJEB46995 |
| SWC23 | Switzerland cattle | Bos taurus | Switzerland | Male | Testis | Pacbio (Sequel IIe) | 33,886,184,244 | 11.54 | 15,858 | 17,584 | ERR11573723 | PRJEB46995 |
| SWC24 | Switzerland cattle | Bos taurus | Switzerland | Male | Testis | Pacbio (Sequel IIe) | 33,196,364,216 | 12.48 | 14,033 | 16,957 | ERR11573724 | PRJEB46995 |
| SWC25 | Switzerland cattle | Bos taurus | Switzerland | Male | Testis | Pacbio (Sequel IIe) | 29,514,585,965 | 9.82 | 12,484 | 14,945 | ERR11573725 | PRJEB46995 |
| SWC26 | Switzerland cattle | Bos taurus | Switzerland | Male | Testis | Pacbio (Sequel IIe) | 32,645,332,394 | 11.93 | 15,703 | 18,063 | ERR11573726 | PRJEB46995 |
| SWC27 | Switzerland cattle | Bos taurus | Switzerland | Male | Testis | Pacbio (Sequel IIe) | 31,551,607,983 | 10.98 | 13,215 | 13,941 | ERR11573727 | PRJEB46995 |
| SWC28 | Switzerland cattle | Bos taurus | Switzerland | Male | Testis | Pacbio (Sequel IIe) | 30,650,248,883 | 10.98 | 10,306 | 11,197 | ERR11573728 | PRJEB46995 |
| SWC29 | Switzerland cattle | Bos taurus | Switzerland | Male | Testis | Pacbio (Sequel IIe) | 36,482,230,912 | 13.45 | 13,069 | 13,566 | ERR11573729 | PRJEB46995 |
| AngBra | Angus x Brahman | Bos indicus x Bos taurus | America | Male | Lung | Pacbio (PacBio RS II) | 531,248,497,494 | 142.76 | 9,599 | 18,305 | SRR6691718-984 | PRJNA432857 |
| Hereford | Hereford | Bos taurus | West Europe | Female | Left Lung | Pacbio (PacBio RS II) | 497,962,231,171 | 107.63 | 7,587 | 16,242 | SRR5753378-721 | PRJNA391427 |
| Wagyu | Wagyu | Bos taurus | Australia | Female | Blood | Nanopore/Pacbio (PromethION/Sequel II) | 956,257,410,920 | 316.07 | 20,787 | 33,112 | SRR30860785-93 | PRJNA1117663 |
| Hanwoo | Hanwoo | Bos taurus | Korea | Male | Blood | Pacbio (Sequel II) | 67,520,132,790 | 21.49 | 19,180 | 20,224 | SRR23238456 | PRJNA927262 |
| Yunling | Yunling cattle | Bos indicus x Bos taurus | China | Male | Heart | Pacbio (Sequel II) | 61,809,420,968 | 19.78 | 17,838 | 18,597 | SRR24831389-90 | PRJNA978937 |

**Table S4**

**Summary of short-reads sequencing data from NCBI**

| **Sample ID** | **Breed** | **Species** | **Sex** | **Tissue** | **Country** | **Mean Depth** | **SRR Accession** | **Bioproject** |
| --- | --- | --- | --- | --- | --- | --- | --- | --- |
| AFA01 | Afar | Bos taurus x Bos indicus | Female | Blood | Africa | 11.87 | SRR12452211 | PRJNA574857 |
| AFA02 | Afar | Bos taurus x Bos indicus | Female | Blood | Africa | 10.74 | SRR12452210 | PRJNA574857 |
| AFA03 | Afar | Bos taurus x Bos indicus | Female | Blood | Africa | 11.13 | SRR12452209 | PRJNA574857 |
| AFA04 | Afar | Bos taurus x Bos indicus | Female | Blood | Africa | 10.68 | SRR12452208 | PRJNA574857 |
| AFA05 | Afar | Bos taurus x Bos indicus | Female | Blood | Africa | 11.23 | SRR12452207 | PRJNA574857 |
| AFA06 | Afar | Bos taurus x Bos indicus | Female | Blood | Africa | 10.24 | SRR12452205 | PRJNA574857 |
| AFA07 | Afar | Bos taurus x Bos indicus | Female | Blood | Africa | 10.95 | SRR12452204 | PRJNA574857 |
| AFA08 | Afar | Bos taurus x Bos indicus | Male | Blood | Africa | 10.34 | SRR12452203 | PRJNA574857 |
| AFA09 | Afar | Bos taurus x Bos indicus | Male | Blood | Africa | 10.91 | SRR12452202 | PRJNA574857 |
| AGS01 | Angus | Bos taurus | Male | Missing | United Kindom | 13.47 | SRR1343172 | PRJNA176557 |
| AGS02 | Angus | Bos taurus | Male | Missing | United Kindom | 13.36 | SRR1346376 | PRJNA176557 |
| AGS03 | Angus | Bos taurus | Male | Missing | United Kindom | 14.85 | SRR1355237 | PRJNA176557 |
| AGS04 | Angus | Bos taurus | Male | Missing | United Kindom | 15.15 | SRR1355239 | PRJNA176557 |
| AGS05 | Angus | Bos taurus | Male | Missing | United Kindom | 16.07 | SRR1365103 | PRJNA176557 |
| AGS06 | Angus | Bos taurus | Male | Missing | United Kindom | 16.46 | SRR1365113 | PRJNA176557 |
| AGS07 | Angus | Bos taurus | Male | Missing | United Kindom | 16.37 | SRR1365129 | PRJNA176557 |
| AGS08 | Angus | Bos taurus | Male | Missing | United Kindom | 17.59 | SRR1365144 | PRJNA176557 |
| AGS09 | Angus | Bos taurus | Male | Missing | United Kindom | 18.08 | SRR1425124 | PRJNA176557 |
| AGS10 | Angus | Bos taurus | Male | Missing | United Kindom | 13.94 | SRR1425145 | PRJNA176557 |
| AGS11 | Angus | Bos taurus | Male | Missing | United Kindom | 17.60 | SRR1425153 | PRJNA176557 |
| Ank01 | Ankole | Bos taurus x Bos indicus | Missing | Missing | Africa | 9.01 | SRR3225527 | PRJNA312138 |
| Ank109 | Ankole | Bos taurus x Bos indicus | Missing | Missing | Africa | 9.17 | SRR3656919 | PRJNA312138 |
| Ank111 | Ankole | Bos taurus x Bos indicus | Missing | Missing | Africa | 9.39 | SRR3656920 | PRJNA312138 |
| Ank123 | Ankole | Bos taurus x Bos indicus | Missing | Missing | Africa | 9.63 | SRR3656922 | PRJNA312138 |
| Ank135 | Ankole | Bos taurus x Bos indicus | Missing | Missing | Africa | 9.86 | SRR3656924 | PRJNA312138 |
| Ank15 | Ankole | Bos taurus x Bos indicus | Missing | Missing | Africa | 9.03 | SRR3656912 | PRJNA312138 |
| Ank50 | Ankole | Bos taurus x Bos indicus | Missing | Missing | Africa | 10.47 | SRR3656915 | PRJNA312138 |
| Ank73 | Ankole | Bos taurus x Bos indicus | Missing | Missing | Africa | 8.24 | SRR3656917 | PRJNA312138 |
| ARS01 | Arsi | Bos indicus | Female | Blood | Africa | 10.33 | SRR12452240 | PRJNA574857 |
| ARS02 | Arsi | Bos indicus | Female | Blood | Africa | 11.05 | SRR12452293 | PRJNA574857 |
| ARS03 | Arsi | Bos indicus | Female | Blood | Africa | 11.76 | SRR12452282 | PRJNA574857 |
| ARS04 | Arsi | Bos indicus | Female | Blood | Africa | 11.24 | SRR12452271 | PRJNA574857 |
| ARS05 | Arsi | Bos indicus | Female | Blood | Africa | 11.02 | SRR12452228 | PRJNA574857 |
| ARS06 | Arsi | Bos indicus | Female | Blood | Africa | 10.63 | SRR12452217 | PRJNA574857 |
| ARS07 | Arsi | Bos indicus | Male | Blood | Africa | 11.68 | SRR12452206 | PRJNA574857 |
| ARS08 | Arsi | Bos indicus | Male | Blood | Africa | 10.74 | SRR12452195 | PRJNA574857 |
| ARS09 | Arsi | Bos indicus | Male | Blood | Africa | 10.27 | SRR12452298 | PRJNA574857 |
| ARS10 | Arsi | Bos indicus | Male | Blood | Africa | 11.52 | SRR12452255 | PRJNA574857 |
| BAR01 | Barka | Bos indicus | Missing | Blood | Africa | 10.09 | SRR12452248 | PRJNA574857 |
| BAR02 | Barka | Bos indicus | Missing | Blood | Africa | 11.08 | SRR12452247 | PRJNA574857 |
| BAR03 | Barka | Bos indicus | Missing | Blood | Africa | 10.86 | SRR12452246 | PRJNA574857 |
| BAR04 | Barka | Bos indicus | Missing | Blood | Africa | 10.79 | SRR12452245 | PRJNA574857 |
| BAR05 | Barka | Bos indicus | Missing | Blood | Africa | 11.10 | SRR12452244 | PRJNA574857 |
| BAR06 | Barka | Bos indicus | Missing | Blood | Africa | 11.51 | SRR12452243 | PRJNA574857 |
| BAR07 | Barka | Bos indicus | Missing | Blood | Africa | 10.02 | SRR12452242 | PRJNA574857 |
| BAR08 | Barka | Bos indicus | Missing | Blood | Africa | 11.03 | SRR12452241 | PRJNA574857 |
| BAR09 | Barka | Bos indicus | Missing | Blood | Africa | 12.29 | SRR12452239 | PRJNA574857 |
| Bo_2612 | Boran | Bos indicus | Missing | Missing | Africa | 9.21 | SRR3546725 | PRJNA312138 |
| Bo_37506E | Boran | Bos indicus | Missing | Missing | Africa | 9.31 | SRR3546727 | PRJNA312138 |
| Bo_392205G | Boran | Bos indicus | Missing | Missing | Africa | 9.19 | SRR3546728 | PRJNA312138 |
| Bo_439 | Boran | Bos indicus | Missing | Missing | Africa | 9.31 | SRR3546729 | PRJNA312138 |
| Bo_467 | Boran | Bos indicus | Missing | Missing | Africa | 9.60 | SRR3546777 | PRJNA312138 |
| Bo_587 | Boran | Bos indicus | Missing | Missing | Africa | 10.37 | SRR3524756 | PRJNA312138 |
| Bo_672 | Boran | Bos indicus | Missing | Missing | Africa | 9.39 | SRR3508267 | PRJNA312138 |
| Bow_1304 | Boran | Bos indicus | Missing | Missing | Africa | 10.20 | SRR3524807 | PRJNA312138 |
| Bow_1688 | Boran | Bos indicus | Missing | Missing | Africa | 10.47 | SRR3524810 | PRJNA312138 |
| BRA01 | Brahman | Bos indicus | Male | Missing | Australia | 15.96 | SRR6650020 | PRJNA432125 |
| BRA02 | Brahman | Bos indicus | Male | Missing | Australia | 15.56 | SRR6650021 | PRJNA432125 |
| BRA03 | Brahman | Bos indicus | Male | Missing | Australia | 15.99 | SRR6650022 | PRJNA432125 |
| BRA04 | Brahman | Bos indicus | Male | Missing | Australia | 16.26 | SRR6650023 | PRJNA432125 |
| BRA05 | Brahman | Bos indicus | Male | Missing | Australia | 11.76 | SRR6650024 | PRJNA432125 |
| BRA06 | Brahman | Bos indicus | Male | Missing | Australia | 12.65 | SRR6650026 | PRJNA432125 |
| BRA07 | Brahman | Bos indicus | Male | Missing | Australia | 10.94 | SRR6650029 | PRJNA432125 |
| BRA08 | Brahman | Bos indicus | Male | Missing | Australia | 17.71 | SRR6650031 | PRJNA432125 |
| BRA09 | Brahman | Bos indicus | Male | Missing | Australia | 11.96 | SRR6650032 | PRJNA432125 |
| BRA10 | Brahman | Bos indicus | Male | Missing | Australia | 11.35 | SRR6649997 | PRJNA432125 |
| MD01 | Burma | Bos indicus | Male | Ear | Burma | 11.40 | SRR12560236 | PRJNA658727 |
| MD02 | Burma | Bos indicus | Male | Ear | Burma | 11.24 | SRR12560238 | PRJNA658727 |
| MD03 | Burma | Bos indicus | Male | Ear | Burma | 8.88 | SRR17381668 | PRJNA658727 |
| MD04 | Burma | Bos indicus | Male | Ear | Burma | 8.86 | SRR17381669 | PRJNA658727 |
| MD05 | Burma | Bos indicus | Male | Ear | Burma | 9.41 | SRR17381670 | PRJNA658727 |
| MD06 | Burma | Bos indicus | Male | Ear | Burma | 8.50 | SRR17381671 | PRJNA658727 |
| MD07 | Burma | Bos indicus | Male | Ear | Burma | 16.53 | SRR17381672 | PRJNA658727 |
| BUT01 | Butana | Bos indicus | Missing | Blood | Africa | 11.72 | SRR12452238 | PRJNA574857 |
| BUT02 | Butana | Bos indicus | Missing | Blood | Africa | 10.15 | SRR12452237 | PRJNA574857 |
| BUT03 | Butana | Bos indicus | Missing | Blood | Africa | 10.21 | SRR12452236 | PRJNA574857 |
| BUT04 | Butana | Bos indicus | Missing | Blood | Africa | 10.19 | SRR12452235 | PRJNA574857 |
| BUT05 | Butana | Bos indicus | Missing | Blood | Africa | 10.45 | SRR12452234 | PRJNA574857 |
| BUT06 | Butana | Bos indicus | Missing | Blood | Africa | 10.20 | SRR12452233 | PRJNA574857 |
| BUT07 | Butana | Bos indicus | Missing | Blood | Africa | 12.13 | SRR12452296 | PRJNA574857 |
| BUT08 | Butana | Bos indicus | Missing | Blood | Africa | 11.08 | SRR12452295 | PRJNA574857 |
| BUT09 | Butana | Bos indicus | Missing | Blood | Africa | 10.39 | SRR12452294 | PRJNA574857 |
| BUT10 | Butana | Bos indicus | Missing | Blood | Africa | 10.83 | SRR12452292 | PRJNA574857 |
| BUT12 | Butana | Bos indicus | Missing | Blood | Africa | 11.42 | SRR12452290 | PRJNA574857 |
| BUT14 | Butana | Bos indicus | Missing | Blood | Africa | 11.74 | SRR12452288 | PRJNA574857 |
| BUT15 | Butana | Bos indicus | Missing | Blood | Africa | 11.72 | SRR12452287 | PRJNA574857 |
| BUT16 | Butana | Bos indicus | Missing | Blood | Africa | 11.99 | SRR12452286 | PRJNA574857 |
| BUT17 | Butana | Bos indicus | Missing | Blood | Africa | 11.95 | SRR12452285 | PRJNA574857 |
| Charolais01 | Charolais | Bos taurus | Male | Missing | France | 17.96 | SRR1343167 | PRJNA176557 |
| Charolais02 | Charolais | Bos taurus | Male | Missing | France | 13.06 | SRR1343168 | PRJNA176557 |
| Charolais03 | Charolais | Bos taurus | Male | Missing | France | 11.00 | SRR1343169 | PRJNA176557 |
| Charolais04 | Charolais | Bos taurus | Male | Missing | France | 16.47 | SRR1348571 | PRJNA176557 |
| Charolais05 | Charolais | Bos taurus | Male | Missing | France | 18.26 | SRR1355258 | PRJNA176557 |
| Charolais06 | Charolais | Bos taurus | Male | Missing | France | 15.45 | SRR1365122 | PRJNA176557 |
| DC01 | Dengchuan | Bos taurus x Bos indicus | Male | Ear | China | 12.07 | SRR12809314 | PRJNA668333 |
| DC02 | Dengchuan | Bos taurus x Bos indicus | Male | Ear | China | 13.10 | SRR12809315 | PRJNA668333 |
| DC03 | Dengchuan | Bos taurus x Bos indicus | Male | Ear | China | 13.18 | SRR12809316 | PRJNA668333 |
| DC04 | Dengchuan | Bos taurus x Bos indicus | Male | Ear | China | 12.29 | SRR12809317 | PRJNA668333 |
| DC05 | Dengchuan | Bos taurus x Bos indicus | Male | Ear | China | 10.57 | SRR12809318 | PRJNA668333 |
| DC06 | Dengchuan | Bos taurus x Bos indicus | Male | Ear | China | 13.62 | SRR12809319 | PRJNA668333 |
| DC07 | Dengchuan | Bos taurus x Bos indicus | Male | Ear | China | 11.96 | SRR12809320 | PRJNA668333 |
| DC08 | Dengchuan | Bos taurus x Bos indicus | Male | Ear | China | 10.15 | SRR12809321 | PRJNA668333 |
| DC09 | Dengchuan | Bos taurus x Bos indicus | Male | Ear | China | 10.17 | SRR12809322 | PRJNA668333 |
| DC10 | Dengchuan | Bos taurus x Bos indicus | Male | Ear | China | 13.27 | SRR12809323 | PRJNA668333 |
| DC11 | Dengchuan | Bos taurus x Bos indicus | Male | Sperm | China | 13.26 | SRR6234766 | PRJNA396672 |
| DC12 | Dengchuan | Bos taurus x Bos indicus | Male | Sperm | China | 13.05 | SRR6234767 | PRJNA396672 |
| FIN01 | Finncattle | Bos taurus | Missing | Missing | Finland | 11.93 | ERR2734942 | PRJEB28185 |
| FIN02 | Finncattle | Bos taurus | Missing | Missing | Finland | 11.53 | ERR2734943 | PRJEB28185 |
| FIN03 | Finncattle | Bos taurus | Missing | Missing | Finland | 11.68 | ERR2734944 | PRJEB28185 |
| FIN04 | Finncattle | Bos taurus | Missing | Missing | Finland | 12.20 | ERR2734945 | PRJEB28185 |
| FIN05 | Finncattle | Bos taurus | Missing | Missing | Finland | 12.20 | ERR2734946 | PRJEB28185 |
| FIN06 | Finncattle | Bos taurus | Missing | Missing | Finland | 11.42 | ERR2734947 | PRJEB28185 |
| FIN07 | Finncattle | Bos taurus | Missing | Missing | Finland | 12.13 | ERR2734948 | PRJEB28185 |
| FIN08 | Finncattle | Bos taurus | Missing | Missing | Finland | 11.65 | ERR2734949 | PRJEB28185 |
| FIN09 | Finncattle | Bos taurus | Missing | Missing | Finland | 11.38 | ERR2734950 | PRJEB28185 |
| FIN10 | Finncattle | Bos taurus | Missing | Missing | Finland | 11.59 | ERR2734951 | PRJEB28185 |
| FOG01 | Fogera | Bos taurus x Bos indicus | Female | Blood | Africa | 10.98 | SRR12452188 | PRJNA574857 |
| FOG02 | Fogera | Bos taurus x Bos indicus | Female | Blood | Africa | 10.56 | SRR12452187 | PRJNA574857 |
| FOG03 | Fogera | Bos taurus x Bos indicus | Female | Blood | Africa | 10.16 | SRR12452186 | PRJNA574857 |
| FOG04 | Fogera | Bos taurus x Bos indicus | Female | Blood | Africa | 10.57 | SRR12452185 | PRJNA574857 |
| FOG05 | Fogera | Bos taurus x Bos indicus | Female | Blood | Africa | 11.74 | SRR12452297 | PRJNA574857 |
| FOG06 | Fogera | Bos taurus x Bos indicus | Female | Blood | Africa | 12.37 | SRR12452264 | PRJNA574857 |
| FOG07 | Fogera | Bos taurus x Bos indicus | Female | Blood | Africa | 10.80 | SRR12452263 | PRJNA574857 |
| FOG08 | Fogera | Bos taurus x Bos indicus | Female | Blood | Africa | 10.32 | SRR12452262 | PRJNA574857 |
| FOG09 | Fogera | Bos taurus x Bos indicus | Male | Blood | Africa | 11.03 | SRR12452261 | PRJNA574857 |
| GVH01 | Gelbvieh | Bos taurus | Male | Missing | Germany | 15.60 | SRR1365112 | PRJNA176557 |
| GVH02 | Gelbvieh | Bos taurus | Male | Missing | Germany | 13.80 | SRR1355236 | PRJNA176557 |
| GVH03 | Gelbvieh | Bos taurus | Male | Missing | Germany | 14.72 | SRR1355240 | PRJNA176557 |
| GVH04 | Gelbvieh | Bos taurus | Male | Missing | Germany | 17.34 | SRR1425154 | PRJNA176557 |
| GVH05 | Gelbvieh | Bos taurus | Male | Missing | Germany | 18.85 | SRR1355260 | PRJNA176557 |
| GVH06 | Gelbvieh | Bos taurus | Male | Missing | Germany | 12.21 | SRR1343161 | PRJNA176557 |
| GVH07 | Gelbvieh | Bos taurus | Male | Missing | Germany | 12.91 | SRR1343162 | PRJNA176557 |
| GVH08 | Gelbvieh | Bos taurus | Male | Missing | Germany | 15.61 | SRR1343164 | PRJNA176557 |
| GVH09 | Gelbvieh | Bos taurus | Male | Missing | Germany | 25.40 | SRR1343165 | PRJNA176557 |
| GVH10 | Gelbvieh | Bos taurus | Male | Missing | Germany | 10.07 | SRR1343166 | PRJNA176557 |
| GVH11 | Gelbvieh | Bos taurus | Male | Missing | Germany | 12.87 | SRR1348575 | PRJNA176557 |
| GVH12 | Gelbvieh | Bos taurus | Male | Missing | Germany | 11.62 | SRR1346388 | PRJNA176557 |
| Gir01 | Gir | Bos indicus | Female | Missing | India | 11.08 | SRS1705836 | PRJNA343262 |
| Gir02 | Gir | Bos indicus | Female | Missing | India | 10.26 | SRS1705837 | PRJNA343262 |
| Gir03 | Gir | Bos indicus | Male | Semen | America | 13.97 | SRR2016752 | PRJNA277147 |
| Gir04 | Gir | Bos indicus | Male | Semen | America | 11.01 | SRR2016754 | PRJNA277147 |
| NEL01 | Nelore | Bos indicus | Male | Semen | Brazil | 10.66 | SRR2016759 | PRJNA277147 |
| NEL02 | Nelore | Bos indicus | Male | Semen | Brazil | 14.66 | SRR2016762 | PRJNA277147 |
| GOF01 | Goffa | Bos indicus | Female | Blood | Africa | 11.15 | SRR12452269 | PRJNA574857 |
| GOF02 | Goffa | Bos indicus | Female | Blood | Africa | 11.42 | SRR12452268 | PRJNA574857 |
| GOF03 | Goffa | Bos indicus | Female | Blood | Africa | 11.04 | SRR12452267 | PRJNA574857 |
| GOF04 | Goffa | Bos indicus | Female | Blood | Africa | 9.10 | SRR12452266 | PRJNA574857 |
| GOF05 | Goffa | Bos indicus | Female | Blood | Africa | 10.27 | SRR12452265 | PRJNA574857 |
| GOF06 | Goffa | Bos indicus | Male | Blood | Africa | 10.60 | SRR12452232 | PRJNA574857 |
| GOF07 | Goffa | Bos indicus | Male | Blood | Africa | 11.56 | SRR12452231 | PRJNA574857 |
| GOF08 | Goffa | Bos indicus | Male | Blood | Africa | 10.81 | SRR12452230 | PRJNA574857 |
| GOF09 | Goffa | Bos indicus | Male | Blood | Africa | 8.45 | SRR12452229 | PRJNA574857 |
| GOF10 | Goffa | Bos indicus | Male | Blood | Africa | 9.71 | SRR12452227 | PRJNA574857 |
| Hanwoo01 | Hanwoo | Bos taurus | Female | Blood | Korea | 12.65 | SRR934415 | PRJNA210519 |
| Hanwoo02 | Hanwoo | Bos taurus | Female | Blood | Korea | 13.16 | SRR934416 | PRJNA210519 |
| Hanwoo03 | Hanwoo | Bos taurus | Female | Blood | Korea | 12.57 | SRR934417 | PRJNA210519 |
| Hanwoo04 | Hanwoo | Bos taurus | Female | Blood | Korea | 13.10 | SRR934418 | PRJNA210519 |
| Hanwoo05 | Hanwoo | Bos taurus | Female | Blood | Korea | 12.10 | SRR934419 | PRJNA210519 |
| Hanwoo06 | Hanwoo | Bos taurus | Female | Blood | Korea | 11.82 | SRR934433 | PRJNA210519 |
| Hanwoo07 | Hanwoo | Bos taurus | Female | Blood | Korea | 11.97 | SRR934434 | PRJNA210519 |
| Hanwoo08 | Hanwoo | Bos taurus | Female | Blood | Korea | 10.76 | SRR934435 | PRJNA210519 |
| Hanwoo09 | Hanwoo | Bos taurus | Female | Blood | Korea | 10.82 | SRR934436 | PRJNA210519 |
| Hanwoo10 | Hanwoo | Bos taurus | Female | Blood | Korea | 14.29 | SRR934432 | PRJNA210519 |
| HER01 | Hereford | Bos taurus | Male | Semen | West Europe | 10.92 | SRR7363678 | PRJNA474946 |
| HER02 | Hereford | Bos taurus | Male | Semen | West Europe | 9.78 | SRR7363666 | PRJNA474946 |
| HER03 | Hereford | Bos taurus | Male | Semen | West Europe | 9.60 | SRR7363668 | PRJNA474946 |
| HER04 | Hereford | Bos taurus | Male | Semen | West Europe | 10.37 | SRR7363667 | PRJNA474946 |
| HER05 | Hereford | Bos taurus | Male | Semen | West Europe | 14.15 | SRR7363665 | PRJNA474946 |
| HER06 | Hereford | Bos taurus | Male | Semen | West Europe | 13.06 | SRR7373661 | PRJNA474946 |
| HER07 | Hereford | Bos taurus | Male | Semen | West Europe | 13.61 | SRR7373660 | PRJNA474946 |
| HER08 | Hereford | Bos taurus | Male | Semen | West Europe | 8.18 | SRR7373658 | PRJNA474946 |
| HER09 | Hereford | Bos taurus | Male | Semen | West Europe | 12.08 | SRR7363653 | PRJNA474946 |
| HER10 | Hereford | Bos taurus | Male | Semen | West Europe | 14.34 | SRR7363649 | PRJNA474946 |
| HER11 | Hereford | Bos taurus | Male | Semen | West Europe | 10.15 | SRR7363650 | PRJNA474946 |
| HER12 | Hereford | Bos taurus | Male | Semen | West Europe | 12.70 | SRR7363664 | PRJNA474946 |
| HOL01 | Holstein | Bos taurus | Female | Hair | West Europe | 32.09 | SRR16937306 | PRJNA780021 |
| HOL02 | Holstein | Bos taurus | Female | Hair | West Europe | 27.07 | SRR16937307 | PRJNA780021 |
| HOL03 | Holstein | Bos taurus | Female | Hair | West Europe | 48.04 | SRR16937308 | PRJNA780021 |
| HOL04 | Holstein | Bos taurus | Female | Hair | West Europe | 30.05 | SRR16937309 | PRJNA780021 |
| HOL05 | Holstein | Bos taurus | Female | Hair | West Europe | 36.21 | SRR16937310 | PRJNA780021 |
| HOL06 | Holstein | Bos taurus | Female | Hair | West Europe | 69.78 | SRR16937311 | PRJNA780021 |
| HOL07 | Holstein | Bos taurus | Female | Missing | Netherlands | 12.81 | SRR1346386 | PRJNA176557 |
| HOL08 | Holstein | Bos taurus | Female | Missing | Netherlands | 12.11 | SRR1346389 | PRJNA176557 |
| HOL09 | Holstein | Bos taurus | Female | Missing | Netherlands | 10.54 | SRR1346392 | PRJNA176557 |
| HOL10 | Holstein | Bos taurus | Female | Missing | Netherlands | 24.46 | SRR1348583 | PRJNA176557 |
| HOR01 | Horro | Bos taurus x Bos indicus | Female | Blood | Africa | 8.39 | SRR12452260 | PRJNA574857 |
| HOR02 | Horro | Bos taurus x Bos indicus | Female | Blood | Africa | 10.25 | SRR12452259 | PRJNA574857 |
| HOR03 | Horro | Bos taurus x Bos indicus | Female | Blood | Africa | 11.22 | SRR12452258 | PRJNA574857 |
| HOR04 | Horro | Bos taurus x Bos indicus | Female | Blood | Africa | 9.07 | SRR12452257 | PRJNA574857 |
| HOR05 | Horro | Bos taurus x Bos indicus | Female | Blood | Africa | 9.88 | SRR12452256 | PRJNA574857 |
| HOR06 | Horro | Bos taurus x Bos indicus | Female | Blood | Africa | 8.97 | SRR12452254 | PRJNA574857 |
| HOR07 | Horro | Bos taurus x Bos indicus | Female | Blood | Africa | 10.47 | SRR12452253 | PRJNA574857 |
| HOR08 | Horro | Bos taurus x Bos indicus | Female | Blood | Africa | 10.20 | SRR12452252 | PRJNA574857 |
| HOR10 | Horro | Bos taurus x Bos indicus | Male | Blood | Africa | 10.64 | SRR12452250 | PRJNA574857 |
| HOR11 | Horro | Bos taurus x Bos indicus | Male | Blood | Africa | 10.68 | SRR12452249 | PRJNA574857 |
| Rashoki01 | Iran taurus | Bos taurus | Female | Missing | Iran | 13.26 | ERR454987 | PRJEB5462 |
| Rashoki02 | Iran taurus | Bos taurus | Female | Missing | Iran | 11.71 | ERR454988 | PRJEB5463 |
| Rashoki03 | Iran taurus | Bos taurus | Female | Missing | Iran | 13.37 | ERR454989 | PRJEB5464 |
| Rashoki04 | Iran taurus | Bos taurus | Female | Missing | Iran | 12.62 | ERR454990 | PRJEB5465 |
| Rashoki05 | Iran taurus | Bos taurus | Female | Missing | Iran | 15.93 | ERR454991 | PRJEB5466 |
| Rashoki06 | Iran taurus | Bos taurus | Female | Missing | Iran | 13.84 | ERR454992 | PRJEB5467 |
| Rashoki07 | Iran taurus | Bos taurus | Female | Missing | Iran | 11.52 | ERR454993 | PRJEB5468 |
| Rashoki08 | Iran taurus | Bos taurus | Female | Missing | Iran | 13.06 | ERR454994 | PRJEB5469 |
| JER01 | Jersey | Bos taurus | Missing | Missing | England | 16.20 | SRR3497161 | PRJNA318089 |
| JER02 | Jersey | Bos taurus | Missing | Missing | England | 18.17 | SRR3497162 | PRJNA318089 |
| JER03 | Jersey | Bos taurus | Missing | Missing | England | 15.29 | SRR3497451 | PRJNA318089 |
| JER04 | Jersey | Bos taurus | Missing | Missing | England | 14.97 | SRR3497462 | PRJNA318089 |
| JER05 | Jersey | Bos taurus | Missing | Missing | England | 14.36 | SRR3497464 | PRJNA318089 |
| JER06 | Jersey | Bos taurus | Missing | Missing | England | 16.59 | SRR3497465 | PRJNA318089 |
| JER07 | Jersey | Bos taurus | Missing | Missing | England | 16.57 | SRR3497466 | PRJNA318089 |
| JER08 | Jersey | Bos taurus | Missing | Missing | England | 17.63 | SRR3497467 | PRJNA318089 |
| JER09 | Jersey | Bos taurus | Missing | Missing | England | 18.84 | SRR3497611 | PRJNA318089 |
| JER10 | Jersey | Bos taurus | Missing | Missing | England | 16.74 | SRR3497613 | PRJNA318089 |
| KENANA_11 | Kenana | Bos indicus | Missing | Missing | Africa | 10.37 | SRR3694653 | PRJNA312138 |
| KENANA_13 | Kenana | Bos indicus | Missing | Missing | Africa | 9.92 | SRR3694654 | PRJNA312138 |
| KENANA_14 | Kenana | Bos indicus | Missing | Missing | Africa | 10.02 | SRR3694655 | PRJNA312138 |
| KENANA_16 | Kenana | Bos indicus | Missing | Missing | Africa | 10.16 | SRR3694656 | PRJNA312138 |
| KENANA_18 | Kenana | Bos indicus | Missing | Missing | Africa | 10.20 | SRR3694658 | PRJNA312138 |
| KENANA_2 | Kenana | Bos indicus | Missing | Missing | Africa | 10.31 | SRR3694651 | PRJNA312138 |
| KENANA_20 | Kenana | Bos indicus | Missing | Missing | Africa | 10.30 | SRR3694659 | PRJNA312138 |
| KENANA_4 | Kenana | Bos indicus | Missing | Missing | Africa | 10.46 | SRR3694652 | PRJNA312138 |
| KENANA_7 | Kenana | Bos indicus | Missing | Missing | Africa | 10.20 | SRR3694657 | PRJNA312138 |
| LC01 | Lincang | Bos indicus | Female | Ear | China | 11.81 | SRR17009033 | PRJNA781760 |
| LC02 | Lincang | Bos indicus | Female | Ear | China | 15.41 | SRR17009034 | PRJNA781760 |
| LC03 | Lincang | Bos indicus | Female | Ear | China | 12.01 | SRR17009035 | PRJNA781760 |
| LC04 | Lincang | Bos indicus | Male | Ear | China | 11.62 | SRR17009036 | PRJNA781760 |
| LC05 | Lincang | Bos indicus | Male | Ear | China | 12.91 | SRR17009037 | PRJNA781760 |
| LC06 | Lincang | Bos indicus | Male | Ear | China | 11.57 | SRR17009038 | PRJNA781760 |
| LC07 | Lincang | Bos indicus | Male | Ear | China | 12.65 | SRR17009039 | PRJNA781760 |
| LC08 | Lincang | Bos indicus | Female | Ear | China | 11.82 | SRR17009040 | PRJNA781760 |
| LC09 | Lincang | Bos indicus | Male | Ear | China | 11.84 | SRR17009041 | PRJNA781760 |
| LC10 | Lincang | Bos indicus | Male | Ear | China | 11.50 | SRR17009042 | PRJNA781760 |
| LC11 | Lincang | Bos indicus | Female | Ear | China | 13.69 | SRR17009043 | PRJNA781760 |
| LC12 | Lincang | Bos indicus | Female | Ear | China | 15.87 | SRR17009044 | PRJNA781760 |
| LC13 | Lincang | Bos indicus | Male | Ear | China | 12.57 | SRR17009045 | PRJNA781760 |
| LC14 | Lincang | Bos indicus | Female | Ear | China | 12.05 | SRR17009046 | PRJNA781760 |
| LC15 | Lincang | Bos indicus | Male | Ear | China | 11.47 | SRR17009047 | PRJNA781760 |
| LC16 | Lincang | Bos indicus | Male | Ear | China | 11.24 | SRR17009048 | PRJNA781760 |
| LC17 | Lincang | Bos indicus | Male | Ear | China | 11.44 | SRR17009049 | PRJNA781760 |
| LC18 | Lincang | Bos indicus | Male | Ear | China | 11.46 | SRR17009050 | PRJNA781760 |
| LC19 | Lincang | Bos indicus | Female | Ear | China | 11.57 | SRR17009051 | PRJNA781760 |
| LC20 | Lincang | Bos indicus | Female | Ear | China | 11.57 | SRR17009052 | PRJNA781760 |
| LC21 | Lincang | Bos indicus | Female | Ear | China | 12.61 | SRR17009053 | PRJNA781760 |
| LC22 | Lincang | Bos indicus | Male | Ear | China | 11.52 | SRR17009054 | PRJNA781760 |
| Luxi01 | Luxi | Bos taurus x Bos indicus | Male | Blood | China | 11.99 | SRR5507242 | PRJNA379859 |
| Luxi02 | Luxi | Bos taurus x Bos indicus | Male | Blood | China | 10.98 | SRR5507241 | PRJNA379859 |
| Luxi03 | Luxi | Bos taurus x Bos indicus | Male | Blood | China | 9.58 | SRR5507240 | PRJNA379859 |
| Luxi04 | Luxi | Bos taurus x Bos indicus | Male | Blood | China | 8.98 | SRR5507239 | PRJNA379859 |
| Luxi05 | Luxi | Bos taurus x Bos indicus | Male | Blood | China | 11.00 | SRR5507238 | PRJNA379859 |
| MA01 | Manie Anjou | Bos taurus | Male | Missing | France | 13.51 | SRR1355238 | PRJNA176557 |
| MA02 | Manie Anjou | Bos taurus | Male | Missing | France | 14.99 | SRR1355245 | PRJNA176557 |
| MA03 | Manie Anjou | Bos taurus | Male | Missing | France | 17.93 | SRR1355259 | PRJNA176557 |
| MA04 | Manie Anjou | Bos taurus | Male | Missing | France | 15.47 | SRR1365125 | PRJNA176557 |
| MA05 | Manie Anjou | Bos taurus | Male | Missing | France | 16.48 | SRR1365127 | PRJNA176557 |
| MA06 | Manie Anjou | Bos taurus | Male | Missing | France | 17.09 | SRR1365130 | PRJNA176557 |
| Mishima01 | Mishima | Bos taurus | Male | Missing | Japan | 11.89 | DRR001771,78,81 | PRJDB2660 |
| Mishima02 | Mishima | Bos taurus | Male | Missing | Japan | 11.67 | DRR001768,75,82 | PRJDB2660 |
| Mishima03 | Mishima | Bos taurus | Male | Missing | Japan | 15.44 | DRR001763,65 | PRJDB2660 |
| Mishima04 | Mishima | Bos taurus | Male | Missing | Japan | 12.85 | DRR001770,77,80 | PRJDB2660 |
| Mishima05 | Mishima | Bos taurus | Male | Missing | Japan | 17.51 | DRR001762,72,74 | PRJDB2660 |
| Mishima06 | Mishima | Bos taurus | Male | Missing | Japan | 14.04 | DRR001769,76,79 | PRJDB2660 |
| Mishima07 | Mishima | Bos taurus | Male | Missing | Japan | 14.63 | DRR001764,66 | PRJDB2660 |
| MG01 | Mongolian | Bos taurus | Male | Ear | China | 13.51 | SRR5507265 | PRJNA379859 |
| MG02 | Mongolian | Bos taurus | Male | Ear | China | 12.38 | SRR5507264 | PRJNA379859 |
| MG03 | Mongolian | Bos taurus | Male | Ear | China | 12.99 | SRR5507263 | PRJNA379859 |
| MG04 | Mongolian | Bos taurus | Male | Ear | China | 13.14 | SRR5507262 | PRJNA379859 |
| MG05 | Mongolian | Bos taurus | Male | Ear | China | 11.47 | SRR5507261 | PRJNA379859 |
| MG06 | Mongolian | Bos taurus | Male | Ear | China | 12.51 | SRR5507266 | PRJNA379859 |
| MG07 | Mongolian | Bos taurus | Male | Ear | China | 12.65 | SRR5507267 | PRJNA379859 |
| NMG12 | Mongolian | Bos taurus | Male | Ear | China | 35.54 | SRR18682940 | PRJNA823481 |
| NMG16 | Mongolian | Bos taurus | Male | Ear | China | 35.38 | SRR18682952 | PRJNA823479 |
| NMG9 | Mongolian | Bos taurus | Male | Ear | China | 31.25 | SRR18682951 | PRJNA823480 |
| MUR01 | Mursi | Bos indicus | Female | Blood | Africa | 10.89 | SRR12452222 | PRJNA574857 |
| MUR02 | Mursi | Bos indicus | Female | Blood | Africa | 10.16 | SRR12452221 | PRJNA574857 |
| MUR03 | Mursi | Bos indicus | Female | Blood | Africa | 10.89 | SRR12452220 | PRJNA574857 |
| MUR04 | Mursi | Bos indicus | Female | Blood | Africa | 12.04 | SRR12452219 | PRJNA574857 |
| MUR05 | Mursi | Bos indicus | Female | Blood | Africa | 10.00 | SRR12452218 | PRJNA574857 |
| MUR06 | Mursi | Bos indicus | Female | Blood | Africa | 9.93 | SRR12452216 | PRJNA574857 |
| MUR07 | Mursi | Bos indicus | Female | Blood | Africa | 9.37 | SRR12452215 | PRJNA574857 |
| MUR09 | Mursi | Bos indicus | Male | Blood | Africa | 10.40 | SRR12452213 | PRJNA574857 |
| MUR10 | Mursi | Bos indicus | Male | Blood | Africa | 10.41 | SRR12452212 | PRJNA574857 |
| MUT01 | Mururu | Bos taurus | Missing | Missing | Africa | 9.07 | SRR5630651 | PRJNA312138 |
| MUT02 | Mururu | Bos taurus | Missing | Missing | Africa | 10.75 | SRR5630650 | PRJNA312138 |
| MUT03 | Mururu | Bos taurus | Missing | Missing | Africa | 11.56 | SRR5630649 | PRJNA312138 |
| MUT04 | Mururu | Bos taurus | Missing | Missing | Africa | 11.04 | SRR5630648 | PRJNA312138 |
| MUT05 | Mururu | Bos taurus | Missing | Missing | Africa | 10.52 | SRR5630647 | PRJNA312138 |
| MUT06 | Mururu | Bos taurus | Missing | Missing | Africa | 9.47 | SRR5630646 | PRJNA312138 |
| MUT08 | Mururu | Bos taurus | Missing | Missing | Africa | 8.36 | SRR5630644 | PRJNA312138 |
| MUT09 | Mururu | Bos taurus | Missing | Missing | Africa | 9.99 | SRR5630653 | PRJNA312138 |
| MUT10 | Mururu | Bos taurus | Missing | Missing | Africa | 9.71 | SRR5630652 | PRJNA312138 |
| Nanyang01 | Nanyang | Bos taurus x Bos indicus | Female | Blood | China | 10.15 | SRR5507237 | PRJNA379859 |
| Nanyang02 | Nanyang | Bos taurus x Bos indicus | Male | Blood | China | 9.13 | SRR5507236 | PRJNA379859 |
| Nanyang03 | Nanyang | Bos taurus x Bos indicus | Male | Blood | China | 12.21 | SRR5507235 | PRJNA379859 |
| Nanyang04 | Nanyang | Bos taurus x Bos indicus | Male | Blood | China | 11.21 | SRR5507234 | PRJNA379859 |
| Nanyang05 | Nanyang | Bos taurus x Bos indicus | Male | Blood | China | 9.91 | SRR5507233 | PRJNA379859 |
| ND064 | N'Dama | Bos taurus | Missing | Missing | Africa | 8.31 | SRR3693229 | PRJNA312138 |
| ND118 | N'Dama | Bos taurus | Missing | Missing | Africa | 9.60 | SRR3693373 | PRJNA312138 |
| ND131 | N'Dama | Bos taurus | Missing | Missing | Africa | 8.63 | SRR3693375 | PRJNA312138 |
| ND148 | N'Dama | Bos taurus | Missing | Missing | Africa | 9.43 | SRR3693376 | PRJNA312138 |
| ND158 | N'Dama | Bos taurus | Missing | Missing | Africa | 8.77 | SRR3693378 | PRJNA312138 |
| ND166 | N'Dama | Bos taurus | Missing | Missing | Africa | 9.09 | SRR3693379 | PRJNA312138 |
| ND169 | N'Dama | Bos taurus | Missing | Missing | Africa | 9.09 | SRR3693419 | PRJNA312138 |
| ND183 | N'Dama | Bos taurus | Missing | Missing | Africa | 10.16 | SRR3693420 | PRJNA312138 |
| ND719 | N'Dama | Bos taurus | Missing | Missing | Africa | 10.47 | SRR3694478 | PRJNA312138 |
| ND730 | N'Dama | Bos taurus | Missing | Missing | Africa | 10.16 | SRR3694578 | PRJNA312138 |
| NEL03 | Nelore | Bos indicus | Missing | Missing | India | 8.92 | SRS1705847 | PRJNA343262 |
| OgD1 | Ogaden | Bos indicus | Missing | Missing | Africa | 9.67 | SRR3234542 | PRJNA312138 |
| OgD2 | Ogaden | Bos indicus | Missing | Missing | Africa | 10.16 | SRR3491019 | PRJNA312138 |
| OgD4 | Ogaden | Bos indicus | Missing | Missing | Africa | 9.90 | SRR3592091 | PRJNA312138 |
| OgD5 | Ogaden | Bos indicus | Missing | Missing | Africa | 9.64 | SRR3219049 | PRJNA312138 |
| OgD6 | Ogaden | Bos indicus | Missing | Missing | Africa | 10.44 | SRR3225526 | PRJNA312138 |
| OgD7 | Ogaden | Bos indicus | Missing | Missing | Africa | 9.90 | SRR3490689 | PRJNA312138 |
| OgD8 | Ogaden | Bos indicus | Missing | Missing | Africa | 10.36 | SRR3508266 | PRJNA312138 |
| OgS1 | Ogaden | Bos indicus | Missing | Missing | Africa | 8.95 | SRR3592093 | PRJNA312138 |
| OgS2 | Ogaden | Bos indicus | Missing | Missing | Africa | 10.14 | SRR3592092 | PRJNA312138 |
| RS01 | Red Sindhi | Bos indicus | Male | Ear | Pakisitan | 10.00 | SRR13114531 | PRJNA658727 |
| RS02 | Red Sindhi | Bos indicus | Male | Ear | Pakisitan | 10.06 | SRR13114532 | PRJNA658727 |
| RS03 | Red Sindhi | Bos indicus | Male | Ear | Pakisitan | 9.39 | SRR13114533 | PRJNA658727 |
| RS04 | Red Sindhi | Bos indicus | Male | Ear | Pakisitan | 10.06 | SRR13114534 | PRJNA658727 |
| RS05 | Red Sindhi | Bos indicus | Female | Ear | Pakisitan | 11.24 | SRR12632082 | PRJNA658727 |
| RS06 | Red Sindhi | Bos indicus | Female | Ear | Pakisitan | 12.45 | SRR12632086 | PRJNA658727 |
| RS07 | Red Sindhi | Bos indicus | Female | Ear | Pakisitan | 11.42 | SRR12632093 | PRJNA658727 |
| RS08 | Red Sindhi | Bos indicus | Female | Ear | Pakisitan | 13.41 | SRR12632104 | PRJNA658727 |
| RS09 | Red Sindhi | Bos indicus | Female | Ear | Pakisitan | 12.33 | SRR12632115 | PRJNA658727 |
| SHE01 | Sheko | Bos taurus x Bos indicus | Female | Blood | Africa | 10.66 | SRR12452198 | PRJNA574857 |
| SHE02 | Sheko | Bos taurus x Bos indicus | Female | Blood | Africa | 12.36 | SRR12452197 | PRJNA574857 |
| SHE03 | Sheko | Bos taurus x Bos indicus | Female | Blood | Africa | 11.65 | SRR12452196 | PRJNA574857 |
| SHE04 | Sheko | Bos taurus x Bos indicus | Female | Blood | Africa | 10.63 | SRR12452194 | PRJNA574857 |
| SHE05 | Sheko | Bos taurus x Bos indicus | Female | Blood | Africa | 11.17 | SRR12452193 | PRJNA574857 |
| SHE06 | Sheko | Bos taurus x Bos indicus | Female | Blood | Africa | 11.24 | SRR12452192 | PRJNA574857 |
| SHE07 | Sheko | Bos taurus x Bos indicus | Female | Blood | Africa | 11.12 | SRR12452191 | PRJNA574857 |
| SHE08 | Sheko | Bos taurus x Bos indicus | Female | Blood | Africa | 10.34 | SRR12452190 | PRJNA574857 |
| SHE09 | Sheko | Bos taurus x Bos indicus | Female | Blood | Africa | 11.18 | SRR12452189 | PRJNA574857 |
| SIM01 | Simmental | Bos taurus | Male | Missing | Switzland | 14.68 | SRR1525617 | PRJNA256210 |
| SIM02 | Simmental | Bos taurus | Male | Missing | Switzland | 12.81 | SRR1525618 | PRJNA256210 |
| SIM03 | Simmental | Bos taurus | Male | Missing | Switzland | 14.09 | SRR1525619 | PRJNA256210 |
| SIM04 | Simmental | Bos taurus | Male | Missing | Switzland | 14.37 | SRR1525620 | PRJNA256210 |
| SIM05 | Simmental | Bos taurus | Male | Missing | Switzland | 12.86 | SRR1525621 | PRJNA256210 |
| SIM06 | Simmental | Bos taurus | Male | Missing | Switzland | 14.15 | SRR1525700 | PRJNA256210 |
| SIM07 | Simmental | Bos taurus | Male | Missing | Switzland | 14.60 | SRR1525701 | PRJNA256210 |
| SIM08 | Simmental | Bos taurus | Male | Missing | Switzland | 14.71 | SRR1525702 | PRJNA256210 |
| SIM09 | Simmental | Bos taurus | Male | Missing | Switzland | 12.45 | SRR1525704 | PRJNA256210 |
| SIM10 | Simmental | Bos taurus | Male | Missing | Switzland | 14.97 | SRR1525705 | PRJNA256210 |
| Thawalam01 | Thawalam | Bos indicus | Male | Ear | Sri Lanka | 12.06 | SRR12578331 | PRJNA658727 |
| Thawalam02 | Thawalam | Bos indicus | Male | Ear | Sri Lanka | 12.28 | SRR12578344 | PRJNA658727 |
| Thawalam03 | Thawalam | Bos indicus | Male | Ear | Sri Lanka | 12.42 | SRR12578345 | PRJNA658727 |
| Thawalam04 | Thawalam | Bos indicus | Male | Ear | Sri Lanka | 14.50 | SRR12578347 | PRJNA658727 |
| Thawalam05 | Thawalam | Bos indicus | Male | Ear | Sri Lanka | 12.72 | SRR12578350 | PRJNA658727 |
| Thawalam06 | Thawalam | Bos indicus | Male | Ear | Sri Lanka | 11.53 | SRR12578351 | PRJNA658727 |
| Thawalam07 | Thawalam | Bos indicus | Female | Ear | Sri Lanka | 11.74 | SRR12578359 | PRJNA658727 |
| TB01 | Tibetan | Bos taurus | Male | Ear | China | 14.69 | SRR5507243 | PRJNA379859 |
| TB02 | Tibetan | Bos taurus | Male | Ear | China | 12.17 | SRR5507244 | PRJNA379859 |
| TB03 | Tibetan | Bos taurus | Male | Ear | China | 11.28 | SRR5507245 | PRJNA379859 |
| TB04 | Tibetan | Bos taurus | Male | Ear | China | 9.67 | SRR5507246 | PRJNA379859 |
| TB05 | Tibetan | Bos taurus | Male | Ear | China | 11.81 | SRR5507247 | PRJNA379859 |
| TB06 | Tibetan | Bos taurus | Male | Ear | China | 13.80 | SRR5507248 | PRJNA379859 |
| TB07 | Tibetan | Bos taurus | Male | Ear | China | 11.30 | SRR5507249 | PRJNA379859 |
| TB08 | Tibetan | Bos taurus | Male | Ear | China | 10.67 | SRR5507250 | PRJNA379859 |
| TB09 | Tibetan | Bos taurus | Male | Ear | China | 10.86 | SRR5507251 | PRJNA379859 |
| WS01 | Wenshan | Bos indicus | Male | Ear | China | 10.46 | SRR6024561 | PRJNA379859 |
| WS02 | Wenshan | Bos indicus | Male | Ear | China | 11.36 | SRR6024562 | PRJNA379859 |
| WS03 | Wenshan | Bos indicus | Male | Ear | China | 11.44 | SRR6024569 | PRJNA379859 |
| WS04 | Wenshan | Bos indicus | Male | Ear | China | 12.21 | SRR6024570 | PRJNA379859 |
| WS05 | Wenshan | Bos indicus | Male | Ear | China | 11.64 | SRR6024575 | PRJNA379859 |
| WS06 | Wenshan | Bos indicus | Male | Ear | China | 12.19 | SRR6024577 | PRJNA379859 |
| YKT01 | Yakutian | Bos taurus | Male | Blood | Northeast Asia | 13.69 | SRR12095766 | PRJNA642008 |
| YKT02 | Yakutian | Bos taurus | Female | Blood | Northeast Asia | 13.70 | SRR12095767 | PRJNA642008 |
| YKT03 | Yakutian | Bos taurus | Female | Blood | Northeast Asia | 13.25 | SRR12095768 | PRJNA642008 |
| YKT04 | Yakutian | Bos taurus | Female | Blood | Northeast Asia | 13.36 | SRR12095769 | PRJNA642008 |
| YKT05 | Yakutian | Bos taurus | Female | Blood | Northeast Asia | 13.67 | SRR12095770 | PRJNA642008 |
| YKT06 | Yakutian | Bos taurus | Female | Blood | Northeast Asia | 11.66 | SRR12095771 | PRJNA642008 |
| YKT07 | Yakutian | Bos taurus | Female | Blood | Northeast Asia | 11.58 | SRR12095772 | PRJNA642008 |
| YKT08 | Yakutian | Bos taurus | Female | Blood | Northeast Asia | 13.36 | SRR12095773 | PRJNA642008 |
| YKT09 | Yakutian | Bos taurus | Female | Blood | Northeast Asia | 11.89 | SRR12095774 | PRJNA642008 |
| YKT10 | Yakutian | Bos taurus | Female | Blood | Northeast Asia | 13.56 | SRR12095775 | PRJNA642008 |
| YKT11 | Yakutian | Bos taurus | Missing | Blood | Northeast Asia | 11.91 | ERR2734952 | PRJEB28185 |
| YKT12 | Yakutian | Bos taurus | Missing | Blood | Northeast Asia | 11.46 | ERR2734953 | PRJEB28185 |
| YKT13 | Yakutian | Bos taurus | Missing | Blood | Northeast Asia | 11.34 | ERR2734954 | PRJEB28185 |
| YKT14 | Yakutian | Bos taurus | Missing | Blood | Northeast Asia | 11.50 | ERR2734955 | PRJEB28185 |
| YBC01 | Yanbian | Bos taurus | Female | Blood | China | 10.40 | SRR5507273 | PRJNA379859 |
| YBC02 | Yanbian | Bos taurus | Male | Blood | China | 31.21 | SRR14879658 | PRJNA737584 |
| YBC03 | Yanbian | Bos taurus | Male | Blood | China | 34.28 | SRR14879660 | PRJNA737584 |
| YBC04 | Yanbian | Bos taurus | Male | Blood | China | 32.64 | SRR14879661 | PRJNA737584 |
| YBC05 | Yanbian | Bos taurus | Male | Blood | China | 46.22 | SRR14879662 | PRJNA737584 |
| YBC06 | Yanbian | Bos taurus | Male | Blood | China | 34.12 | SRR14879663 | PRJNA737584 |
| YBC07 | Yanbian | Bos taurus | Male | Blood | China | 26.95 | SRR14879664 | PRJNA737584 |
| YBC08 | Yanbian | Bos taurus | Male | Blood | China | 29.79 | SRR14879665 | PRJNA737584 |
| YBC09 | Yanbian | Bos taurus | Male | Blood | China | 30.29 | SRR14879666 | PRJNA737584 |
| YBC10 | Yanbian | Bos taurus | Male | Blood | China | 12.84 | SRR6234774 | PRJNA396672 |
| YBC11 | Yanbian | Bos taurus | Male | Blood | China | 12.68 | SRR6234775 | PRJNA396672 |
| HN01 | Hainan | Bos indicus | Male | Blood | China | 31.52 | SRR14879647 | PRJNA737584 |
| HN02 | Hainan | Bos indicus | Male | Blood | China | 31.47 | SRR14879648 | PRJNA737584 |
| HN03 | Hainan | Bos indicus | Male | Blood | China | 28.12 | SRR14879649 | PRJNA737584 |
| HN04 | Hainan | Bos indicus | Male | Blood | China | 30.18 | SRR14879650 | PRJNA737584 |
| HN05 | Hainan | Bos indicus | Male | Blood | China | 38.08 | SRR14879659 | PRJNA737584 |
| HN06 | Hainan | Bos indicus | Male | Blood | China | 31.39 | SRR14879670 | PRJNA737584 |
| HN07 | Hainan | Bos indicus | Male | Blood | China | 31.86 | SRR14879671 | PRJNA737584 |

**Table S5**

**Statics of SV detected detected by integrated software for 83 long-reads sequencing individuals**

| **Sample ID** | **Species** | **Insertion** | **Deletion** | **Duplication** | **Inversion** | **Total** | **Specific SVs** |
| --- | --- | --- | --- | --- | --- | --- | --- |
| panSV | - | 117,671 | 87,276 | 2,730 | 1,355 | 209,032 | 31,809 |
| AGS | Bos taurus | 11,997 | 13,287 | 18 | 108 | 25,410 | 376 |
| SIM | Bos taurus | 12,157 | 13,392 | 27 | 109 | 25,685 | 312 |
| YBC | Bos taurus | 13,374 | 13,217 | 34 | 121 | 26,746 | 458 |
| IRC | Bos taurus | 16,489 | 17,856 | 35 | 142 | 34,522 | 558 |
| MUT | Bos taurus | 13,236 | 12,774 | 35 | 128 | 26,173 | 964 |
| KUR | Bos taurus x Bos indicus | 22,656 | 21,976 | 32 | 172 | 44,836 | 955 |
| IRZ | Bos indicus | 24,440 | 25,953 | 27 | 152 | 50,572 | 1,004 |
| BRA | Bos indicus | 24,925 | 26,035 | 30 | 209 | 51,199 | 960 |
| HN024 | Bos indicus | 30,184 | 29,707 | 24 | 235 | 60,150 | 1,391 |
| HN019 | Bos indicus | 13,896 | 16,166 | 6 | 40 | 30,108 | 444 |
| HN016 | Bos indicus | 15,965 | 18,521 | 9 | 48 | 34,543 | 572 |
| HN08 | Bos indicus | 19,071 | 20,556 | 8 | 80 | 39,715 | 574 |
| HN07 | Bos indicus | 15,230 | 17,563 | 12 | 47 | 32,852 | 481 |
| HN06 | Bos indicus | 21,442 | 23,051 | 15 | 82 | 44,590 | 851 |
| HN05 | Bos indicus | 18,569 | 20,061 | 10 | 59 | 38,699 | 584 |
| HN04 | Bos indicus | 23,537 | 24,339 | 19 | 109 | 48,004 | 933 |
| NMG016 | Bos taurus | 15,392 | 14,963 | 36 | 138 | 30,529 | 655 |
| NMG12 | Bos taurus | 6,647 | 7,569 | 10 | 26 | 14,252 | 121 |
| NMG10 | Bos taurus | 5,634 | 6,577 | 4 | 19 | 12,234 | 136 |
| NMG09 | Bos taurus | 7,240 | 8,063 | 7 | 23 | 15,333 | 162 |
| NMG08 | Bos taurus | 2,893 | 3,610 | 4 | 8 | 6,515 | 47 |
| NMG07 | Bos taurus | 5,279 | 6,166 | 5 | 18 | 11,468 | 127 |
| NMG05 | Bos taurus | 8,835 | 9,610 | 12 | 46 | 18,503 | 193 |
| NMG04 | Bos taurus | 6,282 | 7,315 | 10 | 23 | 13,630 | 136 |
| NMG03 | Bos taurus | 5,478 | 6,356 | 3 | 26 | 11,863 | 96 |
| NMG02 | Bos taurus | 4,315 | 5,029 | 7 | 18 | 9,369 | 58 |
| HN02 | Bos indicus | 38,023 | 36,600 | 122 | 314 | 75,059 | 565 |
| HN01 | Bos indicus | 35,257 | 35,492 | 114 | 199 | 71,062 | 831 |
| YL | Bos indicus | 25,460 | 25,620 | 31 | 155 | 51,266 | 1,122 |
| XX | Bos indicus | 25,141 | 25,548 | 30 | 129 | 50,848 | 1,265 |
| WSGF | Bos indicus | 24,559 | 24,780 | 36 | 127 | 49,502 | 1,192 |
| WZ | Bos indicus | 25,276 | 25,477 | 31 | 123 | 50,907 | 1,259 |
| WN | Bos indicus | 17,721 | 18,296 | 22 | 93 | 36,132 | 637 |
| LCGF | Bos indicus | 25,677 | 25,703 | 42 | 164 | 51,586 | 1,275 |
| LQ | Bos indicus | 28,066 | 28,095 | 41 | 149 | 56,351 | 1,107 |
| GL | Bos indicus | 22,981 | 22,876 | 37 | 152 | 46,046 | 855 |
| DBS | Bos indicus | 24,906 | 24,654 | 31 | 170 | 49,761 | 1,316 |
| JJ | Bos indicus | 27,133 | 26,883 | 39 | 164 | 54,219 | 1,455 |
| BSW0068 | Bos taurus | 11,237 | 10,960 | 33 | 73 | 22,303 | 25 |
| BSW1174 | Bos taurus | 10,624 | 10,618 | 26 | 66 | 21,334 | 35 |
| OBV0854 | Bos taurus | 11,279 | 11,318 | 24 | 76 | 22,697 | 47 |
| OBV1876 | Bos taurus | 10,338 | 10,630 | 15 | 64 | 21,047 | 27 |
| BSW | Bos taurus | 18,250 | 16,962 | 390 | 315 | 35,917 | 64 |
| OBV411 | Bos taurus | 20,422 | 18,999 | 477 | 369 | 40,267 | 15 |
| GS | Bos taurus x Bos indicus | 27,883 | 27,459 | 190 | 313 | 55,845 | 707 |
| DE | Bos taurus x Bos indicus | 20,755 | 19,604 | 66 | 164 | 40,589 | 730 |
| SWC415 | Bos taurus | 12,252 | 11,897 | 46 | 115 | 24,310 | 28 |
| SWC416 | Bos taurus | 11,920 | 11,744 | 47 | 107 | 23,818 | 5 |
| SWC417 | Bos taurus | 12,034 | 11,865 | 45 | 121 | 24,065 | 10 |
| OBV721 | Bos taurus | 11,679 | 11,512 | 36 | 93 | 23,320 | 0 |
| BSN | Bos taurus | 22,822 | 21,873 | 53 | 185 | 44,933 | 11 |
| NB | Bos taurus x Bos indicus | 22,942 | 21,880 | 38 | 195 | 45,055 | 31 |
| OBV | Bos taurus | 18,316 | 17,344 | 180 | 274 | 36,114 | 70 |
| SWC05 | Bos taurus | 8,919 | 9,165 | 20 | 41 | 18,145 | 39 |
| SWC06 | Bos taurus | 8,885 | 8,910 | 14 | 42 | 17,851 | 17 |
| SWC07 | Bos taurus | 9,109 | 9,319 | 15 | 51 | 18,494 | 38 |
| SWC08 | Bos taurus | 9,229 | 9,287 | 9 | 46 | 18,571 | 36 |
| SWC09 | Bos taurus | 8,489 | 8,681 | 12 | 37 | 17,219 | 0 |
| SWC10 | Bos taurus | 9,286 | 9,260 | 16 | 53 | 18,615 | 69 |
| SWC11 | Bos taurus | 8,776 | 8,743 | 13 | 47 | 17,579 | 18 |
| SWC12 | Bos taurus | 9,565 | 9,720 | 14 | 48 | 19,347 | 76 |
| SWC13 | Bos taurus | 9,129 | 9,304 | 8 | 46 | 18,487 | 56 |
| SWC14 | Bos taurus | 7,078 | 7,404 | 10 | 32 | 14,524 | 32 |
| SWC15 | Bos taurus | 8,311 | 8,556 | 15 | 41 | 16,923 | 0 |
| SWC16 | Bos taurus | 8,066 | 8,358 | 18 | 35 | 16,477 | 28 |
| SWC17 | Bos taurus | 8,505 | 8,794 | 15 | 37 | 17,351 | 25 |
| SWC18 | Bos taurus | 9,225 | 9,575 | 19 | 49 | 18,868 | 0 |
| SWC19 | Bos taurus | 9,349 | 9,477 | 17 | 49 | 18,892 | 79 |
| SWC20 | Bos taurus | 6,690 | 7,032 | 10 | 22 | 13,754 | 11 |
| SWC21 | Bos taurus | 7,509 | 7,797 | 14 | 36 | 15,356 | 17 |
| SWC22 | Bos taurus | 8,346 | 8,486 | 10 | 37 | 16,879 | 26 |
| SWC23 | Bos taurus | 8,235 | 8,551 | 16 | 36 | 16,838 | 15 |
| SWC24 | Bos taurus | 7,857 | 8,091 | 18 | 43 | 16,009 | 11 |
| SWC25 | Bos taurus | 7,320 | 7,664 | 10 | 33 | 15,027 | 7 |
| SWC26 | Bos taurus | 8,113 | 8,232 | 12 | 41 | 16,398 | 25 |
| SWC27 | Bos taurus | 7,868 | 8,161 | 13 | 45 | 16,087 | 34 |
| SWC28 | Bos taurus | 7,335 | 7,806 | 16 | 28 | 15,185 | 25 |
| SWC29 | Bos taurus | 8,644 | 8,916 | 13 | 41 | 17,614 | 53 |
| AngBra | Bos taurus x Bos indicus | 20,560 | 19,492 | 26 | 122 | 40,200 | 1,287 |
| Hereford | Bos taurus | 5,002 | 4,514 | 8 | 30 | 9,554 | 167 |
| Wagyu | Bos taurus | 15,394 | 14,822 | 17 | 134 | 30,367 | 642 |
| Hanwoo | Bos taurus | 12,516 | 12,541 | 38 | 94 | 25,189 | 438 |
| Yunling | Bos taurus x Bos indicus | 21,456 | 20,984 | 24 | 129 | 42,593 | 706 |

**Table S6.**

**Summary of Functional Forms of Models**

| **Type** | **Model** | **Equation** |
| --- | --- | --- |
| Linear Model | Linear | y=β0+β1x |
|  | Inverse | y=β0+β1(1/x) |
|  | Logarithmic | y=β0+β1log(x) |
|  | Quadratic | y=β0+β1x+β2x2 |
|  | Inverse and Linear | y=β0+β1x+β2(1/x) |
|  | Constant and Logarithmic | y=β0+β1log(x) |
|  | Inverse and Constan | y=β0+β1(1/x) |
| Non-linear Model | Exponential | y=aexp(bx) |
|  | Power | y=axb |
